# Asking those who know their needs best: A framework for active engagement and involvement of childhood cancer survivors and parents in the process of psychosocial research—A workshop report

**DOI:** 10.1002/cnr2.2071

**Published:** 2024-05-20

**Authors:** Liesa J. Weiler‐Wichtl, Carina Schneider, Hannah Gsell, Anna‐Maria Maletzky, Anita Kienesberger, Claas Röhl, Albina Bocolli, Johannes Gojo, Rita Hansl, Anna Zettl, Maximilian Hopfgartner, Ulrike Leiss

**Affiliations:** ^1^ Department of Pediatrics and Adolescent Medicine, Comprehensive Center for Pediatrics and Comprehensive Cancer Center Medical University of Vienna Vienna Austria; ^2^ KOKON – Psychosocial and Mental Health in Pediatrics Lab Rohrbach‐Berg Upper Austria Austria; ^3^ Childhood Cancer International – Europe (CCI‐E) Vienna Austria; ^4^ Survivors Austria Vienna Austria; ^5^ NF Kinder Vienna Austria; ^6^ Department of Cognition, Emotion, and Methods in Psychology, Faculty of Psychology University of Vienna Vienna Austria

**Keywords:** interdisciplinary health teams, neuropsychology, patient and public involvement and engagement, pediatric cancer, survival, workshop

## Abstract

**Background:**

Patient and public involvement and engagement (PPIE) in healthcare research is crucial for effectively addressing patients' needs and setting appropriate research priorities. However, there is a lack of awareness and adequate methods for practicing PPIE, especially for vulnerable groups like childhood cancer survivors.

**Aims:**

This project aimed to develop and evaluate engagement methods to actively involve pediatric oncological patients, survivors, and their caregivers in developing relevant research questions and practical study designs.

**Methods and Results:**

An interdisciplinary working group recruited *n* = 16 childhood cancer survivors and their caregivers to work through the entire process of developing a research question and a practicable study design. A systematic literature review was conducted to gather adequate PPIE methods which were then applied and evaluated in a series of three workshop modules, each lasting 1.5 days. The applied methods were continuously evaluated, while a *monitoring group* oversaw the project and continuously developed and adapted additional methods. The participants rated the different methods with varying scores. Over the workshop series, the participants successfully developed a research question, devised an intervention, and designed a study to evaluate their project. They also reported increased expertise in PPIE and research knowledge compared to the baseline. The project resulted in a practical toolbox for future research, encompassing the final workshop structure, evaluated methods and materials, guiding principles, and general recommendations.

**Conclusion:**

These findings demonstrate that with a diverse set of effective methods and flexible support, actively involving patients, survivors, and caregivers can uncover patients' unmet disease‐related needs and generate practical solutions apt for scientific evaluation. The resulting toolbox, filled with evaluated and adaptable methods (workbook, Supplement 1 and 2), equips future scientists with the necessary resources to successfully perform PPIE in the development of health care research projects that effectively integrate patients' perspectives and address actual cancer‐related needs. This integration of PPIE practices has the potential to enhance the quality and relevance of health research and care, as well as to increase patient empowerment leading to sustainable improvements in patients' quality of life.

## 1 INTRODUCTION

Childhood cancer is one of the most severe diseases with complex medical and psychosocial effects on the lives of the affected patients and their families. Among the most frequently discussed acute effects, patients and caregivers report physical pain and depletion, emotional instability and distress, social isolation as well as a considerable economic and administrative burden.^1^ Numerous guidelines and psychosocial interventions have been developed to ensure that pediatric patients and their families receive adequate support during these incomparably burdening times.^2^ However, childhood cancer can also lead to numerous long‐term consequences such as chronic fatigue and pain, reduced academic and occupational prospectives.^3^, ^4^ While these effects considerably impact the everyday quality of life of childhood cancer survivors, they are less obvious to the psychosocial care services and often overseen in the process of transferring from pediatric to adult medical care.^5^, ^6^, ^7^

To ensure that the affected population is not overwhelmed but their experience‐based expertise is integrated in the development of psychosocial care services, the concept of Public and Patient Involvement and Engagement (PPIE) in research has been developed.^8^, ^9^ The core idea of PPIE and related concepts (e.g., community‐based participatory research,^10^ user‐involvement^11^) is to involve childhood cancer patients, survivors and their caregivers into the design and development of health care and psychosocial research to help professionals in better understanding and addressing patients' needs.^12^ Among the benefits associated with PPIE in pediatric oncology, researchers name the increased relevance of research, higher patient satisfaction, more effective recruitment of participants and better acceptance and feasibility of research findings.^13^, ^14^, ^15^ However, despite a considerable growth in scientific interest in the topic as well as its inclusion into research and care standards, there is still a lack of general awareness for the topic and deficient practical implementation, especially in the most defining early research phases.^8^, ^15^, ^16^, ^17^, ^18^

Besides lacking awareness, heterogenous terminology and insufficient reporting of results, one major barrier to the sustainable establishment of PPIE is the lack of effective methodologies for its implementation, especially for vulnerable groups such as pediatric oncological patients.^8^, ^18^, ^19^, ^20^ In principle, the National Institute of Health Research (NIHR) distinguishes between passive *participation* (e.g., as study participants); active *involvement* into the research process; and interactive *engagement* of the patient community, disseminating information about research (e.g., lay summaries, scientific festival, workshops, and focus groups).^9^ Judging from the current state of literature, the majority of PPIE projects in pediatric oncology are based only on patient *participation* with patients testing the interventions designed for them.^21^, ^22^, ^23^ In contrast, only few studies actively *engage* with or *involve* the patient group in the research process especially in the most defining, early stages of research. While research tend to argue childhood cancer patients are simply too hard to involve into research, initiatives such as the BRIGHTLIGHT project have shown that, given adequate support, young cancer survivors can make an invaluable contribution to ongoing research.^24^ Since existing efforts to standardize PPIE practices^18^, ^25^, ^26^, ^27^, ^28^, ^29^ hardly consider the specific needs and conditions of childhood cancer patients and survivors, concrete guidelines and protocols are necessary to facilitate effective implementation of PPIE practices for the researchers.

One common and effective method of practicing *patient engagement* are workshops and focus groups, where theoretical knowledge is not only transmitted to the participants in an adequate and interactive manner, but also created as a result of communication and collaboration.^10^ Preceding research has provided excellent examples for such PPIE workshops and focus groups, where the *engagement* was not only used to educate the patient group but to generate recommendations on the allocation of resources and priority setting,^30^, ^31^ to investigate unmet patient needs and to improve care,^32^, ^33^ to research the establishment of PPIE,^16^ to construct new research paradigms and frameworks,^34^ to discuss and establish new methodologies for risk stratification^35^ or to investigate the establishment of PPIE.^8^ Thereby a variety of concrete methods was used, ranging from interview guides,^32^ full or break‐out group discussions and deliberation,^31^ and traditional lectures and educative videos,^35^ to creative novel activities. These include expert and patient advisory panels,^36^, ^37^ role play and perspective taking^31^, ^35^ and visual and spatial preparation of information.^38^ To evaluate the *engagement* and the concrete methods, questionnaires,^22^, ^23^, ^32^, ^39^, ^40^ transcript analysis,^31^, ^37^, ^41^ thematic analysis of recorded content and feedback,^35^, ^42^ visual mapping of results,^43^ and directed content analysis have been used.^38^ Many of the encountered studies were designed and led by multidisciplinary steering groups with authors highlighting first‐hand insights by the affected population and the synthesis of diverse perspectives as a major benefit of *engagement*.^16^, ^41^, ^44^ Taken together, active PPIE research has brought forth numerous methods to effectively engage with the patient community and to harness their expertise in the development of psychosocial care for pediatric oncology. However, concrete recommendations and practicable guidelines as well as the awareness for the benefits of PPIE are lacking to facilitate the implementation and active practice of PPIE in childhood cancer research.^8^

Based on the results of an online survey and a pilot workshop of the authors' preceding project (for details see Reference 8), the present study aims to integrate existing evidence‐based methodologies for *patient engagement* into a practicable workshop protocol as a best practice example for the development of research projects including PPIE practices. In addition to a replicable workshop design, a PPIE workbook including concrete engagement methods, easy to use workshop materials and experience‐based, practice‐oriented recommendations shall be developed. To this end, an interdisciplinary research team of health care professionals (HCPs) and patient experts will design a workshop with multiple modules in which childhood cancer patients, survivors and caregivers are asked to go through all stages of the scientific research process for the development of a psychosocial tool addressing a need of their choice. In an iterative process, the chosen methods shall be evaluated by the participants and the workshop content of the consecutive modules adapted by the research team. The present report aims to present the final workshop structure as well as the evaluated methods and the resulting recommendations and guidelines summarized in the publicly available workbook.^45^, ^46^, ^47^

## 2 METHODS

### 2.1 Research team

Throughout the study procedure, various groups of experts in different fields are involved in the different steps. While the *working group* designed and conducted the study, the *monitoring group* consisting of external stakeholders assisted in evaluating the proceedings and methods to continuously adapt the workshop structure and content to the experiences and evaluations. This follows the EUPATI recommendations for PPIE in health research.^48^ The term *research academy* refers to the workshop participants who evaluated the methods, gave feedback on the workshop and in the realm of the workshop developed a research proposal to address a disease‐related psychosocial need of their choice. Table 1 provides an overview of the different groups of people, their composition, the tasks they are involved in as well as the project stages they accompanied.

**TABLE 1:** Relevant groups contributing to the construction, conduct and evaluation of the study.

Group name	Description and tasks	Composition
Working group	Systematic literature review to identify adequate methods for the workshop modules, recruit participants for research academy, conduct and moderate workshops, integrate expert opinions and evaluations of monitoring group in upcoming workshop plan, deduct recommendations for future workshops, draft scientific publication and workbook with recommendations	min: 3 psychologists, 1 patient expert (module specific alterations with additional experts)
Monitoring group	Responsible for the study design, quality control, continuous evaluation and adjustment of workshop content and proceedings^48^	4 psychologists, 1 medical doctor, 3 patient experts, 2 patient advocates (*n* = 10)
Research academy (Workshop participants)	Recruited by the working group, the members of the research academy participate in the workshops, work through an exemplary research process, develop study design and evaluate the applied methods	*n* = 13 survivors (thereof *n* = 3 underaged) and *n* = 3 parents of former patients

### 2.2 Study procedure

The *monitoring group* started in a first *kick‐off meeting*, creating a provisional project design. In a first step, the *working group* conducted a *systematic literature review* of scientific data bases to derive evidence‐based ideas and models for the design of a PPIE workshop in the field of pediatric oncology. The applied search strategy as well as the resulting list of relevant publications can be found in Table S1 and Figure S2. Based on the expert opinion, experience in the preceding project^8^ and encountered resources, the tentative number of workshop participants was set to 10 people who were recruited via an online call, disseminated via social media as well as mailing lists and contacts within the local hospital. The *monitoring group* continuously supervised and evaluated to process and workshop proceedings, and based upon the feedback, the working group repeatedly adjusted the workshop design. Upon final evaluation by all three groups, the resulting workshop structure, methods, and general recommendations were integrated into a workbook to provide future researchers with a toolbox for effective PPIE including concrete instructions and materials.

**FIGURE 1:**
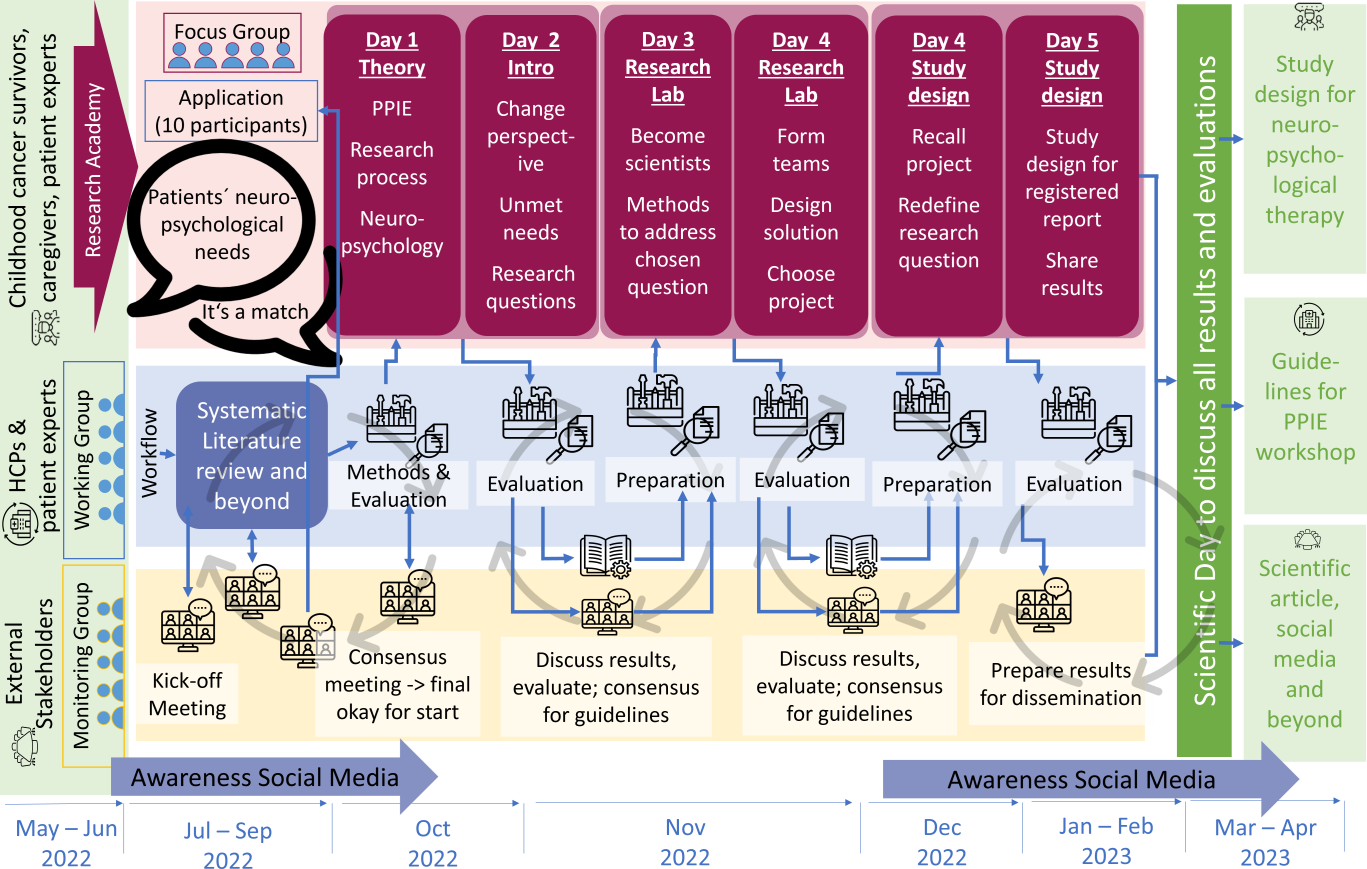
Project workflow including the preparation, execution, evaluation, and publication of the workshop.

Figure 1 visualizes the final project trajectory containing the tasks fulfilled within the three layers of *research academy*, the *working group*, and the *monitoring group*.

### 2.3 Workshop design

Based on the experience of the pilot workshop in the preceding project (see Reference 8) the workshop series was designed to consist of three modules (one weekend each), the contents of which shall resemble the consecutive steps in scientific research.^9^ The working group conducted a systematic literature review to identify and preselect evaluated PPIE methods, tools and activities for each workshop module. Following the Plan‐Do‐Study‐Act model by Schurman et al.^49^ the concrete content for each module was continuously adjusted to the participants' input during the workshop and modified based on the evaluation and reflection by the *monitoring group* after each module.

### 2.4 Workshop methods and evaluation

The final selection of workshop methods was drawn from a systematic literature review (Table S1 and Figure S2) and the professional experience of working group members. The evaluation of the methods was based on a 10‐point Likert scale. Recurring questions were posed in a consistent manner throughout the consecutive modules to allow for longitudinal analysis. Additionally, the working group prepared self‐assessment questions for each workshop, to assess the participants knowledge on the presented topics, their needs within steps in scientific research, as well as their attitude towards raised questions. While knowledge was assessed through closed‐ended questions, needs and attitudes were identified through brainstorming and free association. Due to its facilitation of repeated assessments and the diverse interactive evaluation options, the online survey tool *menti* was used to administer all evaluations and questionnaires.^50^ Due to the small sample size and the longitudinal nature of the data, Cramer's *V* test for dependence was used to compare the self‐rated knowledge at the different time points.

## 3 RESULTS

### 3.1 Workshop participants

The final sample of workshop participants was constituted by a total of *n* = 16 participants who were recruited predominantly from the hosting university clinic by their treating personnel and via the connections built in the preceding PPIE project.^8^ The group encompassed *n* = 13 survivors (thereof *n* = 3 underaged) and *n* = 3 parents of former patients. In their application *n* = 4 defined themselves as complete novices regarding the topic of PPIE, *n* = 6 rated themselves as beginners, *n* = 5 called themselves advanced and *n* = 1 already felt like an expert.

### 3.2 Workshop structure

**FIGURE 2:**
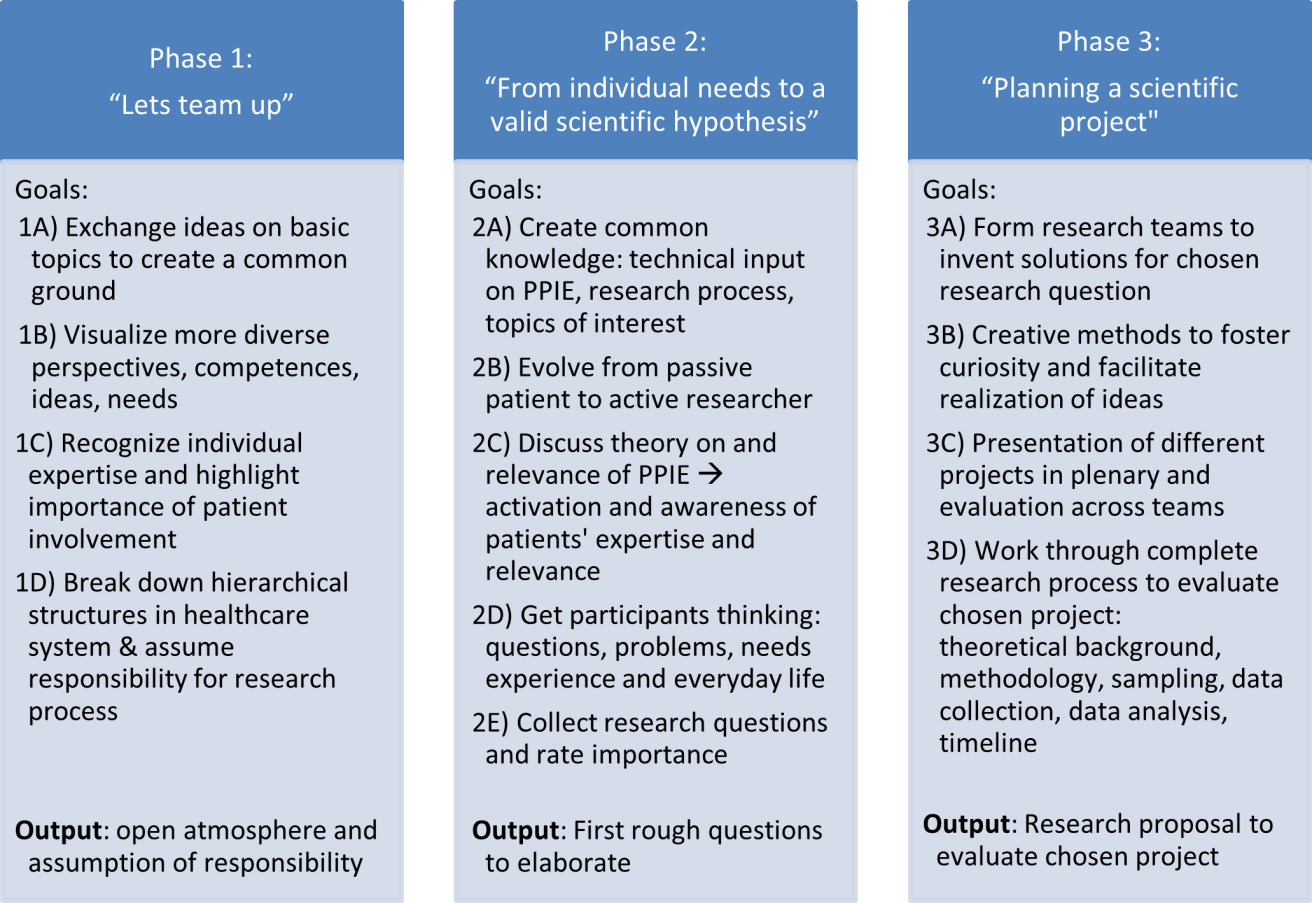
Key workshop phases including the main objectives and output.

Since the workshop structure was developed in an iterative process of continuous evaluation, discussion and adaptation via the *working* and *monitoring group* and in the interaction with the patient group, it can be considered the primary result of this study. The final structure, as envisaged in the original workshop concept, consisted of a series of three modules, each lasting 2 days (Friday: noon–evening, and Saturday: morning–evening). The final workshop content can be divided into three core phases depicted in Figure 2.

The aim of the first phase, entitled “Let's team up,” is to give participants the space to move from being passive patients to active researchers. This was realized by first providing theoretical input on the core concepts discussed in the workshop (PPIE, neuropsychology, and research process). During and after the presentations and informative videos, the participants are given the space to exchange common experiences and their ideas on the basic themes of the workshop in order to create a common ground and a shared knowledge space. Moreover, the expertise of each stakeholder (HCP, patient, family, etc.) is discussed to create awareness for the different perspectives and their relevance and to break down the hierarchical structures of the health system. Finally, research groups are formed to enforce the team spirit and provide the basis for the active elaboration of the core topics in the consecutive phases. The question the participants of the present workshop decided on was “How can I find my way back into everyday life after a neuro‐oncological illness?”

The second phase is called “From individual needs to a valid scientific hypothesis” and aims to identify the core topics proposed by the participants. This active discussion of relevant issues builds upon the theoretical foundation and the content discussed in the first phase. The final goal of openly discussing the patients' everyday challenges and disease‐related needs is to define a relevant research question that can be elaborated into a research project in phase 3. In the present project, the participants needed more time than anticipated to identify themselves as researchers and to move from the role of being a passive patient to actively deciding on a topic. Moreover, additional methods (visualization of topics, grouping by theme, and ratings) had to be inserted into the workshop to condense the large number of proposed issues and to converge on one unifying research question.

**FIGURE 3:**
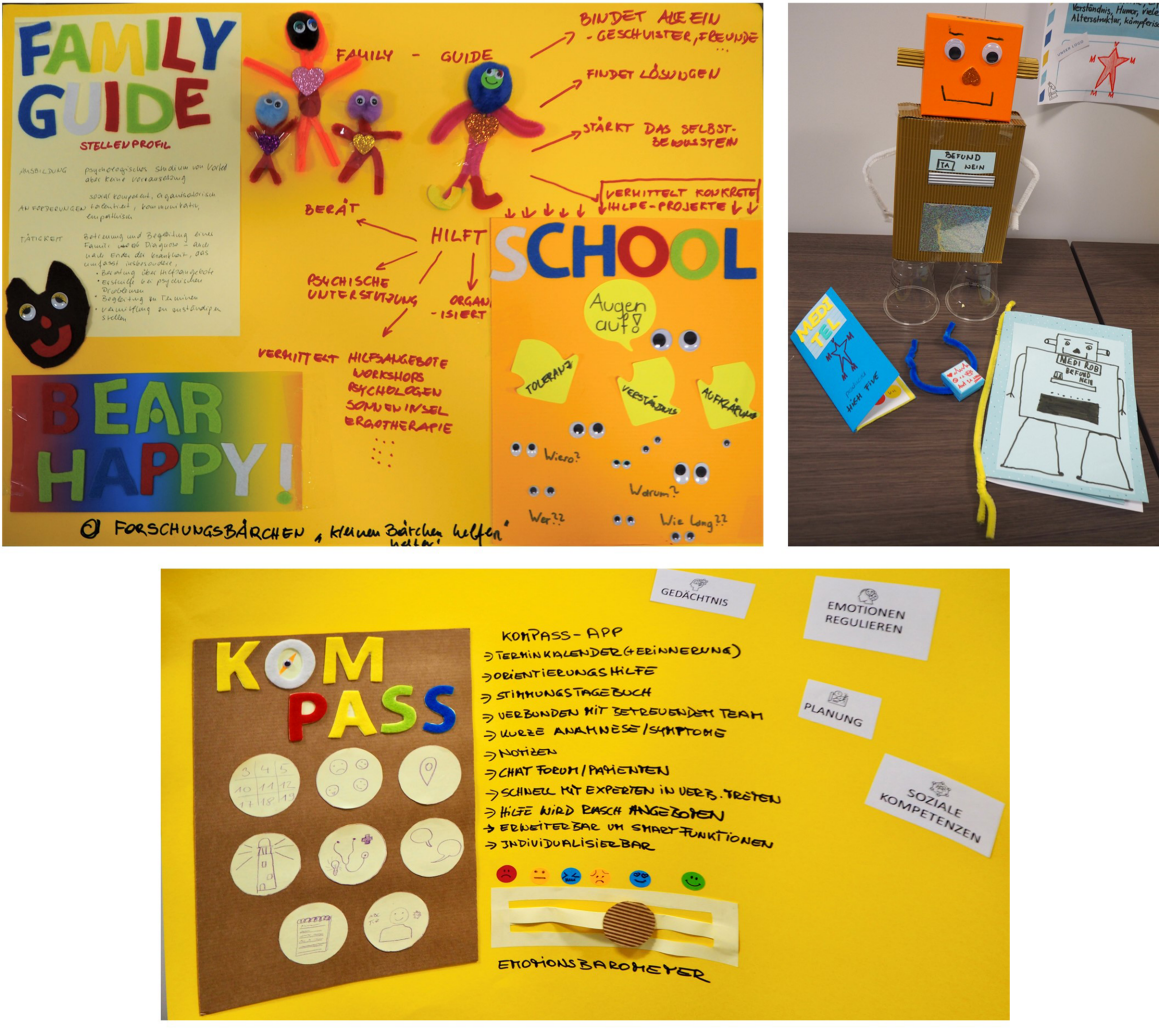
Photos of the interventions developed by the three subgroups within the workshop.

In the final phase called “Planning a scientific project,” participants work in research groups to find creative solutions to the needs discussed earlier. In the present project, a total of three groups was formed, each developing an independent project idea. Once the core ideas were finished, each group presented their work. The posters for the project presentations are depicted in Figure 3. After the presentations, the whole group voted for their favorite project proposal which in this case was the “Family guide” (FG). This tool shall provide patients, survivors and their caregivers with information, help to organize themselves (e.g., legal matters, cooperation with school during the treatment, and transition from care to after‐care), support them in school and inform the environment about potential special needs.

In the course of *phase 3*, the initial research question was further specified leading to the final research question: “Does the Family Guide change the subjectively perceived quality of care in pediatric hematology/oncology in Austria?” Together with the whole group, the winning project was embedded in a scientific process through the development of a study design and a research proposal for the evaluation of the developed instrument. The framework used was the structure of a registered report in an adequate journal, including topics that shall be discussed in the introduction, a methodology for evaluating the designed intervention, and a proposed timeline for the conduct of the tentative study. Table 2 provides an overview of the key information on the exemplary project developed in the present workshop.

**TABLE 2:** Study design for evaluating the designed intervention in form of a registered report.

Introduction
Need for shared decision making during oncological illness Importance of a bottom‐up approach for patients with cancer (Care Service Science concept) Evidence that parental mental health is closely related to children's mental health Need to educate physicians about patients' individual needs Positive impact of psychosocial measures on late effects and costs in the health care system Supportive care concept (“Bezugspflegekonzept”) to relieve affected patients Recommended support via organizational and social measures for families “Shared Care”—linking pediatric oncologists with primary care physicians Challenges in transition as need for Family Guide Various apps already exist => emphasize importance of orientation (connection to database) Database with information on patients which Family Guide should also have access to Family Guide (FG) necessary to support in coping with the disease
Methodology
*Study sample*: Patients (≥5 years of age) (inclusion criteria‐entry question: “Have you been diagnosed with a pediatric oncologic disease?” Siblings (≥5 years). Both parents (differentiation based on initial question in FB, who is involved to what extent in care, etc.) All families treated in Austria (regardless of main residence; different psychosocial care systems between the provinces).
*Experimental procedures*: Literature review Group comparison/cross over model Focus group interview on the development of questionnaire Pre‐survey via FG (patients, parent, sibling—own FB depending on population). Pre‐collection of objective data (e.g., duration between diagnosis and 1st psychology contact, etc.) Post‐survey via FG Post‐survey based on objective data Comparison of objective data with subjective opinions
Project timeline
Duration: 2–5 years Start: survey control group already during FG training. survey before implementation of the FG during treatment after completion of treatment Either 30 families and FG for 6 months or 15 families and FG for 1 year (normal distribution within the groups endangered due to *n* < 30)

### 3.3 Workshop methods and evaluation

Each workshop day was filled with diverse interactive activities, evidence‐based methods, and creative tools to transmit the intended content as well as to realize all steps towards the final aim of a concrete research proposal tackling a specific need. The concrete methods employed in the present workshop series can be found in Table 3.

**TABLE 3:** Overview of workshop structure, addressed aims as defined in Figure 2, applied methods, and evaluation.

Module	Section	Aim	Methods	Evaluation [0;10]			
				Mean	Median	25%/75%	Min/max
Module 1—Day 1	PPIE	1A 1B 1C	Educative movie^51^ Free association Quiz	8.6	9	[8;10]	[5;10]
	Research process	1B 1C 1D	*Marketplace*: Designing a film reel about the steps involved in the scientific process Quiz	5.6	5.5	[5;7]	[0;10]
	Neuropsychological therapy	1A 1B	Marketplace Quiz				
Module 1—Day 2	Team building	1B 1C 1D	*Worksheets for designing personal avatar* ^52^ *Presentation* in plenary	6.9	7	[5.5;7.5]	[5;10]
				6.6	8	[4;9]	[2;10]
	Change in perspective	1A 1B 1C	*Worksheets for designing professional avatar* (researcher/HCP)^53^ *Brainstorming*/collecting ideas	9.1	10	[8;10]	[7;10]
	Unmet needs and first research questions	1A 1C 1D	*Island hopping*: stations for different contexts in pediatric neuro‐oncology^38^ Collection of raised issues in plenary *Voting for most relevant issue*	7.5	7.5	[5.5;9.75]	[4;10]
				8.0	8.5	[6.25;10]	[4;10]
Module 2—Day 1	Introduction and repetition	2A	Summary by one member of the working group				
	Neuropsychological domains and their relevance	2B 2C 2D 2E	*Workstations*: Defining issues, wishes, and possible solutions regarding np domains regarding four research questions (write on sticky notes)^43^ Collect notes on separate posters for problem and solution in each domain	9.3	10	[10;10]	[6;10]
	Assessment of relevance	2B 1D	*Rating* with sticky notes (the more, the more relevant)	6.9	8	[5;9.5]	[0;10]
Module 2—Day 2	Group identity	2B 1D 3A	*Worksheet*: group name, symbol, motto, aim	6.9	7	[5.5;7.5]	[5;10]
	Gaining an understanding of a scientific process	2A 2D	*Books*: Stories about renown researchers^54^ Plenary exchange and discussion	3.7	3	[1;6]	[0;9]
	Invent solution to chosen neuropsychological issue	2B 3B	*Break out groups*: encouraged to use handicraft materials				
	Presentation and assessment of relevance	2B 2E 3C	*Elevator pitches* in plenary Rating favorite project using stickers	6.6	8	[4;9]	[2;10]
Module 3—Day 1	Reconstruction of last module	3A	Summary by one member of the working group				
	Further narrowing of the chosen neuropsychological issue	3D	*Work sheet*: Miracle Question^55^	6.8	7	[6;7]	[5;10]
	Presentation of resulting opinions and ideas	3D	*Chart Board Discussion* ^42^	3.3	2	[1;4]	[0;10]
Module 3—Day 2	Defining an ultimate research question	3C 3D	Plenary discussion	n.a.			
	Development of a study design	3D	Operationalization: free association in plenary Individual digital literature research Consideration of further questions on methodology, sampling and timetable using a worksheet in plenary	n.a.			
	Preparation of an abstract draft	3D	Writing up the study design in plenary	n.a.			

After each activity, the appeal of the applied methods was evaluated by the participants using an online questionnaire. In the course of the workshop, the rating processes became an important ritual, providing structure and reassurance since the same format was maintained, while all other methods kept changing. The scores (range: [0;10]) for each evaluated method are displayed in Table 3. To evaluate the effectiveness of the workshop in educating the participants, they were additionally asked to assess their own knowledge about the core topics of the workshop (PPIE, scientific research, and neuropsychological therapy) at the beginning and at the end of each workshop module. Concerning their knowledge about PPIE, participants reported significantly higher knowledge at the end of module 1 compared to its beginning (*V* = 36, *p* = .010). However, this knowledge did not significantly change further in module 3 (*V* = 10, *p* = .089). Regarding their knowledge about scientific work, participants reported significantly increased knowledge after module 1 (*V* = 75, *p* = .038) and module 3 (*V* = 34, *p* = .031). Finally, participants reported a significant increase in knowledge about neuropsychological therapy during module 1 (*V* = 51, *p* = .015), but not module 3 (*V* = 10, *p* = .098).

### 3.4 General feedback and learnings

Throughout the project, the continuous evaluation and adaptation of the workshop content and structure via the feedback of participants as well as the discussion with the *monitoring group* allowed for important learnings and recommendations for future workshops. The final, adjusted workshop concept including the major findings, practical recommendations as well as the designed material was summarized into a publicly available workbook^45^, ^46^, ^47^ which can be found in supplement 1 and 2.

Major practical takeaways from the participants feedback were the relevance of short sessions to maintain the energy, the need for continuous availability of food and drinks, relevance of appropriate lighting and accessibility as well as accommodation for special neuropsychological needs and support (e.g., visual impairment, reading assistance, epilepsy, migraine, and movement constraints) to prevent a systematic exclusion of perspectives. In addition, the content needed to be as concrete and practical as possible to facilitate communication and avoid confusion with abstract constructs. This was achieved by using worksheets based on the reality of the participants' lives. In addition, the worksheets encouraged small group work and visualized each step of the process. At the beginning of the modules, some of the previous content was summarized using the worksheets to reintroduce the participants to the topic. To conclude, if patients are to be genuinely involved, it should be ensured that their needs are taken into account at the workshop stage, so that these are not considered as a barrier or disadvantage to their participation in the group and being represented. This includes cognitive impairments, such as poor impulse control, reduced skills in delaying needs, reduced attention and/or memory, and more.

## 4 DISCUSSION

Overall, the present PPIE project represents a promising, replicable framework for actively engaging patients, survivors, and their caregivers in the design of novel research to effectively tackle their actual needs. The flexible workshop structure and the creative and openminded exploration (thinking out of the box without any limits) were crucial for fruitful outcomes with the potential to facilitate patient empowerment.^56^, ^57^ The first phase (“Let's team up”) was essential to deliberately provide participants with sufficient space for personal interaction and exchange of common experience. Moreover, the emphasis on the relevance of diverse perspectives and experience‐based expertise allowed for the breakdown of hierarchical structures to facilitate effective open communication and collaboration. Despite a subjective gain in knowledge about PPIE, the participants took more time than anticipated to identify as researchers during the second phase (“From individual needs to a valid scientific hypothesis”) Moreover, the richness and number of ideas on relevant everyday life issues surpassed the expected amount, necessitating flexible adaptation and provision of additional time and space. However, given sufficient time and space, the participants converged on one issue which was effectively translated into a concrete research question. In the final phase (“Planning a scientific project”) participants successfully developed their chosen research question into a feasible project proposal addressing their most pressing disease‐related neuropsychological issue. In line with preceding PPIE research,^14^, ^15^, ^28^ the resulting study design as a final outcome of the workshop series highlights that the active involvement and engagement of childhood cancer survivors is not only possible but invaluable when trying to establish meaningful psychosocial research that follows patient priorities.

Beyond serving as a best practice example to show that PPIE in pediatric oncology is generally possible, feasible and beneficial, the present project aimed at providing future research with a rich and practicable toolbox to facilitate more effective patient involvement. A systematic literature search as well as professional experience by the working group yielded a large number of diverse methods and materials used during the workshop to guide the participants during their engagement with the entire scientific research process.^8^, ^16^, ^38^, ^42^ The results of the continuous patient evaluation of the methods showed that certain methods were preferred over others, indicating the need for flexible adaptation of the workshop structure and content to the individual needs of the specific sample.^58^ Among the most favored methods were open space activities allowing for active interaction as well as materials facilitating creative exploration of the discussed topics (e.g., References 38, 43, 51). In contrast, the more lecture‐like and information‐dense activities were appreciated less by the participants although the modules including such content led to stronger increase in the patients' self‐reported knowledge about the topic. Overall, the variety in activities as well as the continuous evaluation and adaptation thereof allowed the *working group* to identify and develop methods that would most effectively support the patients in understanding and realizing their expertise. The final selection of methods and developed materials is publicly available^45^, ^46^, ^47^ and provided in the supplemental material (supplement 1 and 2). With the provision of concrete guidelines and a practicable framework, the present project aims at lowering the threshold for future research to apply PPIE in the development of health care research and care projects.

The continuous feedback by the participants as well as the discussion and reflection of the *working group* with a *monitoring group* including various external stakeholders (as suggested by Reference 48) allowed for the development of meta‐level recommendations and guidelines for patient engagement in pediatric oncology. Among the most practically relevant findings are the need for inclusive accessibility, sufficient support by qualified staff, enough breaks and short sessions as well as the comprehensive consideration of potential disease‐related needs.^3^, ^59^ Hereby visual impairments and language barriers for terms in non‐native language had to be accommodated for. However, following previous recommendations,^24^ the repeated application of consistent methods and sufficient time and personal support, increased the participants' proficiency in using the tools (e.g., online survey with digital device) and allowed for disease‐related obstacles to be overcome. The organization and workshop moderation via a qualified, interdisciplinary team of HCPs and patient experts was necessary to mediate the interaction between different stakeholders and allow for unprecedented insights into patients', survivors', and caregivers' everyday struggles to be recorded. The developed workbook provides future researchers with a comprehensive list of clear recommendations. While these recommendations do reflect the complexity of specific needs of the affected population, it also shows that with adequate adaptation and support it is possible and feasible to address and overcome these barriers and ultimately achieve the goal of effectively involve of childhood cancer patients, survivors and their families into health care research and development.

Although the iterative process of continuous evaluation, adaptation, and development^60^ increased the complexity and resource intensity of the present project, it yielded a clear and feasible research proposal as well as a replicable workshop framework, a workbook full of practicable tools and concrete guidelines and recommendations for future PPIE workshops. However, in addition to the availability of tools and methods, more awareness for the benefits of PPIE are necessary to foster openness to new methods and unexpected findings as well as self‐reflection among researchers.^61^ Moreover, binding research standards including PPIE might be necessary to counter the believe that the involvement of childhood cancer patients into research is not feasible, too research intensive or too complex.^16^ However, the outcome of a feasible and scientifically sound research proposal creatively addressing patients' actual needs highlights the relevance and benefits of patient and public involvement, especially in early research stages which should be considered worth the extra effort.

To conclude, the resulting workshop structure and the developed intervention show that psychosocial care and in pediatric oncology are complex processes, necessitating openness to new research approaches and communication on eye‐level as well as a variety of effective and individually adaptable methods.^16^, ^48^, ^58^ To facilitate the implementation of similar workshops for future research, the developed methods and materials as well as guiding principles and general recommendations are integrated into a workbook. This collection of materials and advice shall serve health care professionals and researchers as a practical toolbox and concrete guideline for realizing their own, individually adapted workshop design. By making the results and methods publicly available,^45^, ^46^, ^47^ we hope to contribute to the sustainable establishment of PPIE as an integral part of scientific research in the health care sector to ensure that patients' needs are identified effectively and addressed adequately. Given patients' and researchers' openness to these new, creative methods and the willingness to perform critical self‐reflection, a sustainable shift towards a more patient‐centered research paradigm can be achieved.

## 5 CONCLUSION

Patient and public involvement and engagement in health care research is an invaluable way of increasing the quality and relevance of research, and patient empowerment and satisfaction, yet it lacks effective implementation and estimation. In the presented study, the active involvement of oncological patients, survivors, and caregivers into the design of feasible research projects allowed for the definition of relevant research questions and the development of creative solutions. Moreover, the systematic assessment of survivors' perspectives highlighted that careful task formulation, and the sincere consideration of disease consequences are necessary for effective communication and collaboration between professionals and the patient group. By fulfilling the aim of developing PPIE workshop guidelines and a set of evaluated methodologies, the present project provides future researchers with all the necessary resources to actively integrate the affected population in their research. This shall lay the basis for higher feasibility and accessibility of effective PPIE, to better address the diverse and evolving needs of pediatric oncology patients and their caregivers.

## AUTHOR CONTRIBUTIONS

**Liesa J. Weiler‐Wichtl:** Conceptualization (equal); formal analysis (equal); funding acquisition (equal); investigation (equal); methodology (equal); project administration (equal); resources (equal); supervision (equal); visualization (equal); writing – original draft (equal); writing – review and editing (equal). **Carina Schneider:** Conceptualization (equal); funding acquisition (equal); investigation (equal); methodology (equal); supervision (equal); writing – review and editing (equal). **Hannah Gsell:** Conceptualization (equal); investigation (equal); methodology (equal); writing – original draft (equal); writing – review and editing (equal). **Anna‐Maria Maletzky:** Conceptualization (equal); investigation (equal); methodology (equal); writing – original draft (equal); writing – review and editing (equal). **Anita Kienesberger:** Conceptualization (equal); methodology (equal); supervision (equal); writing – review and editing (equal). **Claas Röhl:** Conceptualization (equal); supervision (equal). **Albina Bocolli:** Project administration (equal). **Johannes Gojo:** Conceptualization (equal); methodology (equal); supervision (equal); writing – review and editing (equal). **Rita Hansl:** Formal analysis (equal); visualization (equal); writing – original draft (equal); writing – review and editing (equal). **Anna Zettl:** Methodology (equal); writing – review and editing (equal). **Maximilian Hopfgartner:** Data curation (equal); formal analysis (equal); visualization (equal); writing – original draft (equal); writing – review and editing (equal). **Ulrike Leiss:** Conceptualization (equal); investigation (equal); methodology (equal); resources (equal); supervision (equal); writing – review and editing (equal).

## FUNDING INFORMATION

This work was fully funded by the Ludwig Boltzmann Institute (PPIE Call 2021).

## CONFLICT OF INTEREST STATEMENT

The authors have stated explicitly that there are no conflicts of interest in connection with this article.

## ETHICS STATEMENT

Not applicable.

## CONSENT TO PARTICIPATE

Written informed consent was obtained from all individual participants included in the study or their parents in case of children.

## CONSENT TO PUBLISH

The authors affirm that human research participants provided informed consent for publication of the images in Figure 3 as well as the content of Table 2.

## Supporting information


**Supplementary Table 1** Overview of articles resulting from the systematic literature review on PPIE tools in pediatric oncology.

**Supplementary Figure 1** Flowchart of systematic literature review on PPIE tools in pediatric oncology.

**Supplementary Material 1:** PPIE workshop guidelines.

**Supplementary Material 2:** PPIE Lab Book.

## Data Availability

The datasets generated during and/or analyzed during the current study are available from the corresponding author on reasonable request.
