# Supplementary figures and images for: Asking those who know their needs best: A framework for active engagement and involvement of childhood cancer survivors and parents in the process of psychosocial research—A workshop report
Source: Cancer Rep (Hoboken). 2024 May 20;7(5):e2071. doi: 10.1002/cnr2.2071 (PMC11104286; doi:10.1002/cnr2.2071)

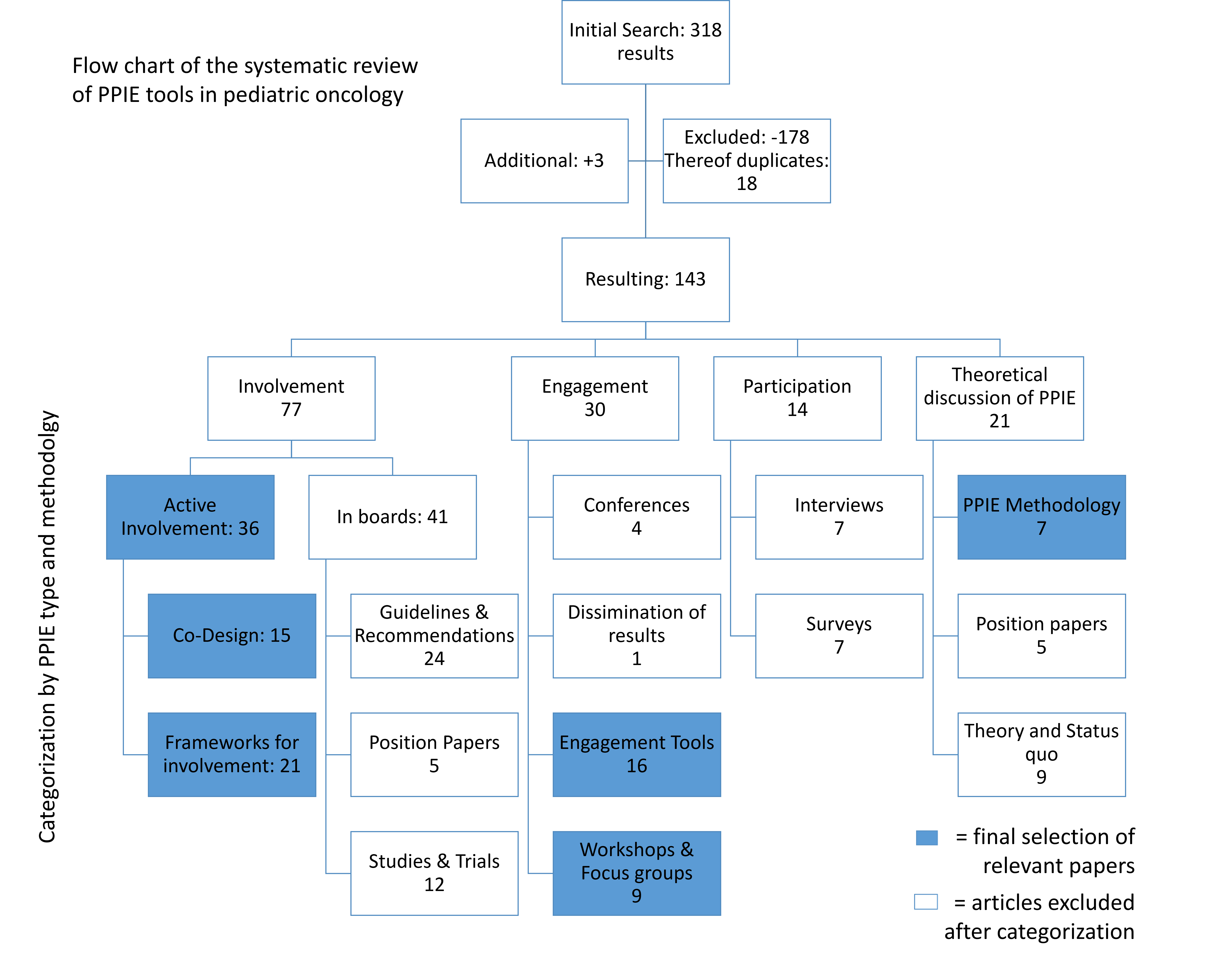

Supplement: Supplementary file 2 — Supplementary Figure 1 Flowchart of systematic literature review on PPIE tools in pediatric oncology. [file CNR2-7-e2071-s001.zip › Supp_2_flow_chart.png]

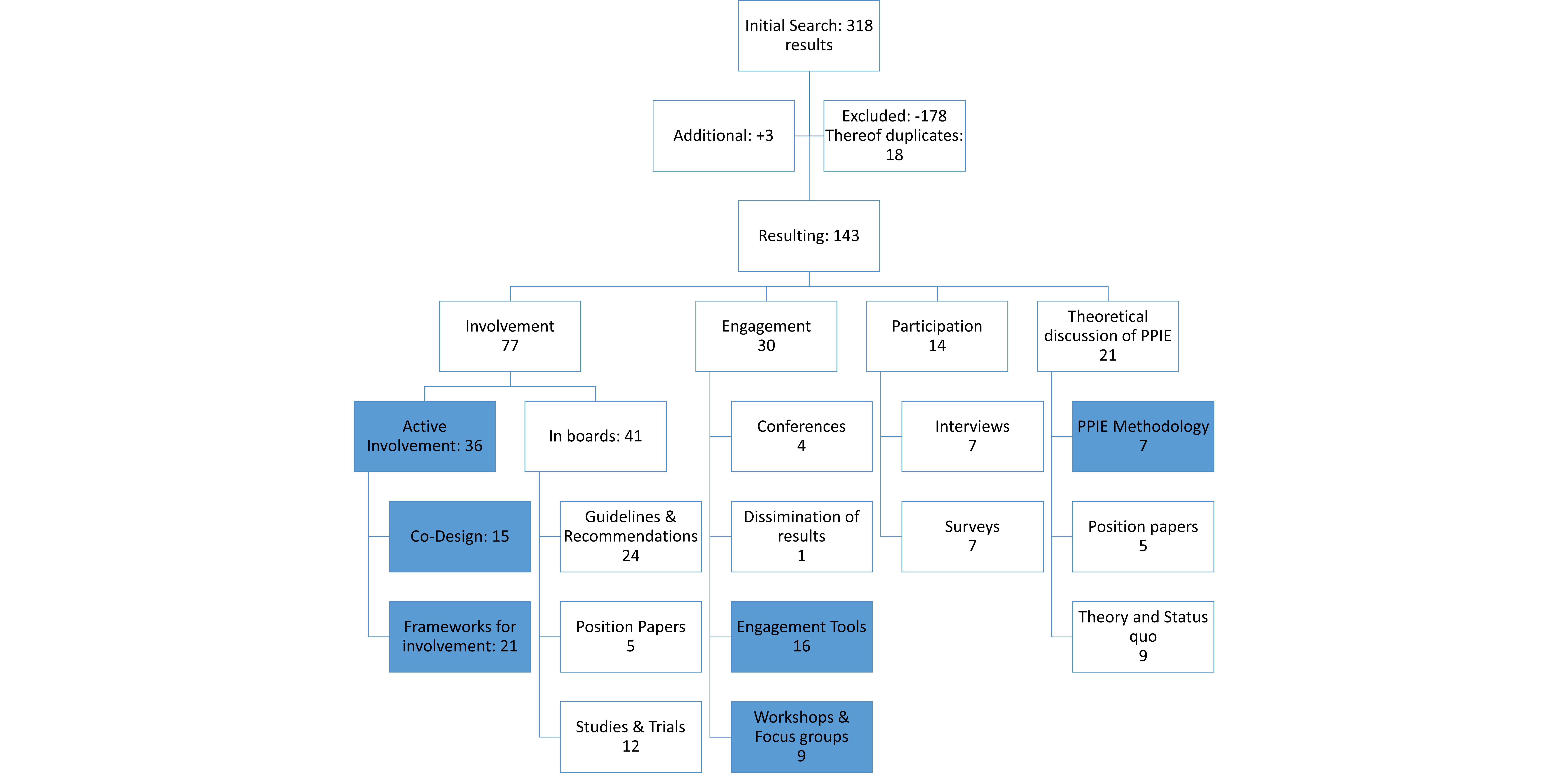

Supplement: Supplementary file 2 — Supplementary Figure 1 Flowchart of systematic literature review on PPIE tools in pediatric oncology. [file CNR2-7-e2071-s001.zip › Supplement 2_flow chart.png]
