# Supplementary material for: Asking those who know their needs best: A framework for active engagement and involvement of childhood cancer survivors and parents in the process of psychosocial research—A workshop report
Source: Cancer Rep (Hoboken). 2024 May 20;7(5):e2071. doi: 10.1002/cnr2.2071 (PMC11104286; doi:10.1002/cnr2.2071)
Supplement: Supplementary file 3 — Supplementary Material 1: PPIE workshop guidelines. [file CNR2-7-e2071-s004.pdf]

BRIDGING THE GAP

GEMEINSAM FORSCHEN

Workshop für eine ernsthafte Patient:inneneinbindung in Forschung und Entwicklung

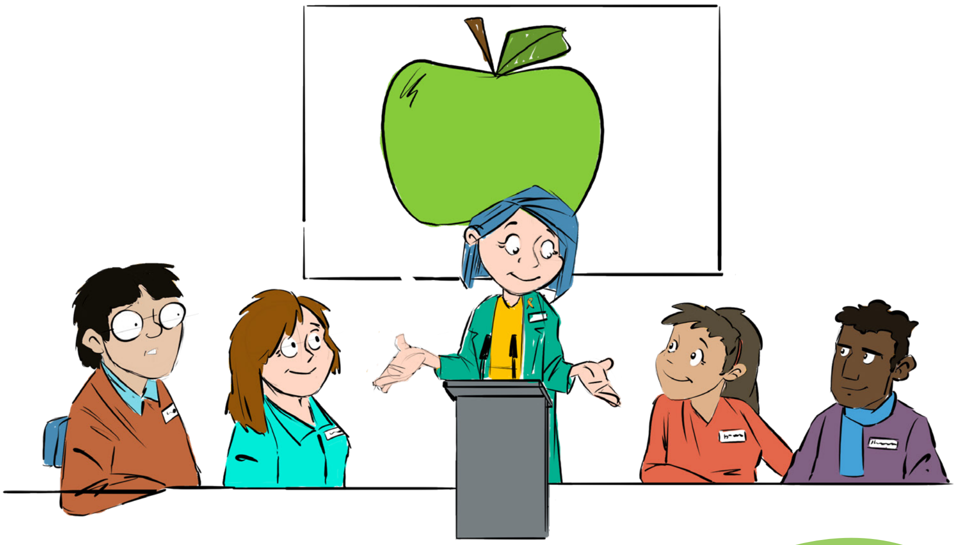

Moderator:innen-Edition

Vorsicht: Praktische Anleitung zum freudvollen Forschen voraus!

Finanziert durch:

LUDWIG BOLTZMANN GESELLSCHAFT
Open Innovation in Science Center

Umgesetzt von:

MEDIZINISCHE UNIVERSITÄT WIEN

COPE GROUP

Childhood Cancer International EUROPE

KURVENKRATZER

Vorwort

Hallo!

Mein Name ist Claire und ich habe ein Anliegen.
Ich möchte, dass besser verstanden wird, wie es ist, in einen **sauren Apfel** zu beißen. Jetzt wundern sich wahrscheinlich viele Leser:innen:
Worauf will sie hinaus?

Nun, der Apfel ist meine Metapher für Krebs. Ich habe ihn überlebt.
Und mein Pfad zur Genesung hat mich zu einer echten **Expertin** gemacht:
Ich weiß alles über meinen besonders sauren Apfel.
Vor allem, wie er schmeckt.

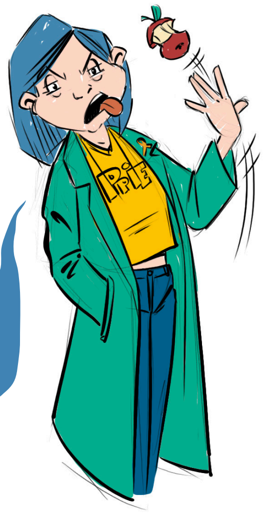

Das führt mich zur **Vision**, die mein Forschungsteam und ich haben:
Die Öffentlichkeit und Patient:innen in die Forschung einzubinden.
Und diese dadurch zu verbessern, dass Forschungsfragen zielgenau gestellt werden. Damit jene Menschen im Mittelpunkt stehen, um die es geht: Die Öffentlichkeit und Patient:innen wie ich.

Was das mit dem Apfel zu tun hat? Nun, es gibt ja bereits umfangreiche Methoden, einen Apfel zu vermessen, zu analysieren und zu behandeln. Und es gibt Expert:innen, die mit sehr viel **Know-how und Enthusiasmus** daran arbeiten. Manche ahnen es schon: Gemeint sind Forschende – etwa aus der Medizin, der Psychologie, der Pflege, der sozialen Arbeit und vielen weiteren Feldern.

Jetzt kommen wir zum Kern der Sache (und zum Zweck dieses Dokuments):
Um **wirksame Diagnostikmethoden und Therapien** zu entwickeln, braucht es neben dem Vermessen, Analysieren und Behandeln eben auch den Erfahrungswert, den Patient:innen mitbringen. Denn nur sie können wirklich beschreiben, wie der Apfel schmeckt. Es geht also um die Einbindung von Patient:innen in die Forschung, von Anfang an. Darum dreht sich der **Workshop**, zu dem du eingeladen wurdest. Er dient als Werkzeug, das Patient:innen und Forschungsgruppen ermöglicht, gemeinsam **Forschungsfragen mit hoher Relevanz zu entwickeln**. Wissenschaftliche Fragen, die uns Patient:innen und unsere Zugehörigen von Anfang an einbinden und unsere Expertise und Bedürfnisse ernst nehmen. Und davon ausgehend **Projektpläne**, um die Forschung gemeinsam umzusetzen.

Das gilt für alle Bereiche, in denen Forschung passiert! Wie bei einem Puzzle müssen wir alle Expertisen, die den Körper, die Psyche und das Soziale betreffen, gleichermaßen berücksichtigen, um am Ende ein Gesamtbild zu erhalten und allen Bedürfnissen gerecht zu werden. Eine ganzheitliche, interdisziplinäre Grundhaltung nach dem biopsychosozialen Modell ist daher nötig – auch bei der Auswahl der Expert:innen.

In diesem Workshop behandeln wir das konkrete Thema:

„Denkt daran, den Teilnehmer:innen Infos zum Workshopthema zur Verfügung zu stellen. Sei es durch Videos, Literatur oder Webseiten. Am besten schickt ihr diese Infos zusammen mit dem Lab-Book per E-Mail.“

Vorwort

Mit diesem Dokument klappt das bestimmt!
Es handelt sich um eine **Schritt-für-Schritt-Anleitung** für die Moderator:innen des Workshops und enthält zahlreiche praktische Infos: Von der Vorbereitung über die Durchführung bis hin zur Evaluierung und Dokumentation. Ich selbst tauche auch immer wieder auf und gebe nützliche Tipps aus meinem Erfahrungsschatz in der partizipativen Forschung.

Eine Grundvoraussetzung für eine gute Zusammenarbeit ist, dass alle Teilnehmer:innen trotz ihres diversen Hintergrunds **gleichberechtigt** mitmachen können. Nur so kann das Ziel erreicht werden: Bridging the Gap! Also **eine Brücke schlagen** zwischen Patient:innen und Forschenden. Denn wir arbeiten alle auf dasselbe hin: Wir wollen den Apfel in seinen Facetten verstehen. Eben auch, wie er schmeckt. Denn wir alle wollen die **Forschung und Versorgung verbessern**.

Viel Spaß beim Workshop!

Claire,
Patient Expert

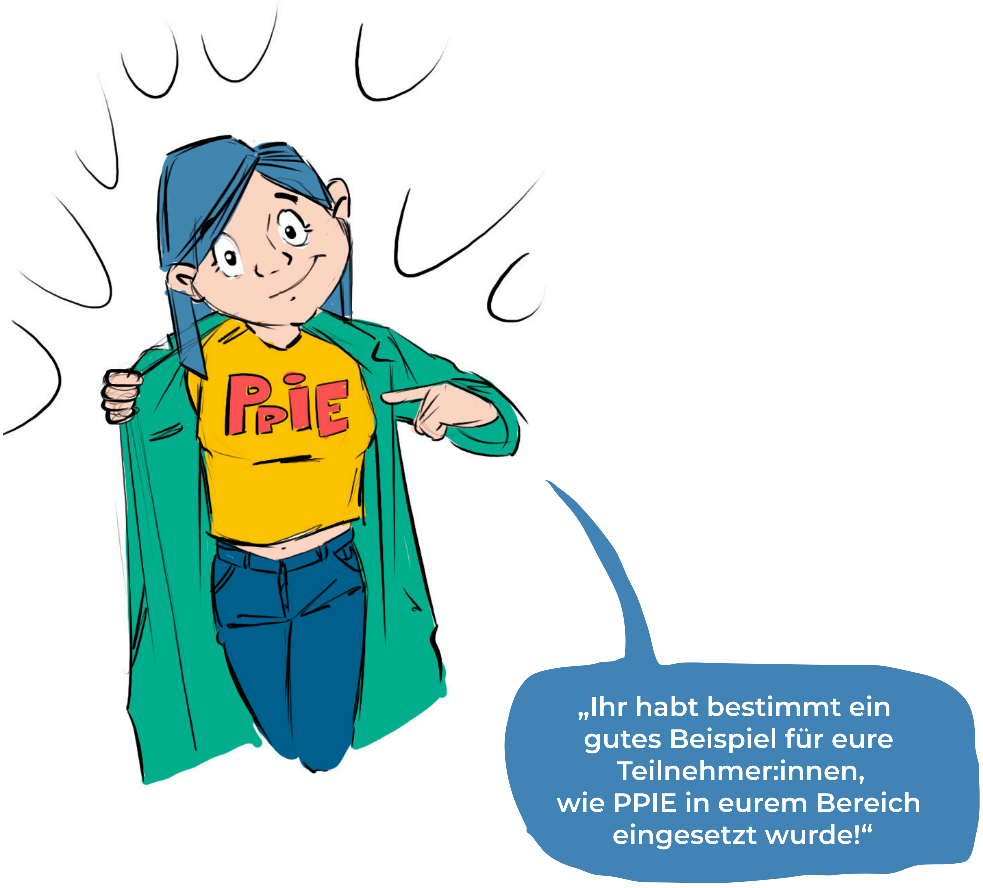

Inhalt

INTRO 7

Warum Patient and Public Involvement & Engagement (PPIE)? 7

SO GELINGT DIE OPTIMALE WORKSHOP-VORBEREITUNG 9

- Moderation organisieren 9
- Raum wählen & Logistik prüfen 9
- Material & Unterlagen zusammenstellen 10
- Die geeignete Gruppe finden 11
- Berücksichtigung besonderer Bedürfnisse 12
- Spielregeln definieren 13
- Vorbereitung der Teilnehmer:innen 14

DER WORKSHOP 15

- Roadmap zu den Modulen 1- 4 15

MODUL 1: DER EINSTIEG 17

- Vorbereitung und Ziele 17
- Methoden 1-8 18-24

MODUL 2: FORSCHUNGSFRAGEN FORMULIEREN 25

- Vorbereitung und Ziele 25
- Methoden 9-15 26-31

MODUL 3: KREATIVE LÖSUNGEN FINDEN 33

- Vorbereitung und Ziele 33
- Methoden 16-18 34-36

MODUL 4: STUDIENDESIGN ENTWICKELN 37

- Vorbereitung und Ziele 37
- Methoden 19-25 38-43

WAS ES NACH DEM WORKSHOP BRAUCHT 45

- Evaluierung durchführen 45
- Dokumentation abwickeln 45
- Wie geht es weiter? 45

ZUM SCHLUSS 47

Weiterführende Literatur **49**
Impressum **51**
Anhang:
1. „Lab-Book“ zum Versenden an die Teilnehmer:innen:
   Intro, Erklärung PPIE, Spielregeln, PPIE-Wörterbuch, Arbeitsblätter, Gedankenparkplatz
2. Präsentationen: PPIE & Forschungsprozess, Studiendesign-Wegweiser

GEDANKENPARKPLATZ

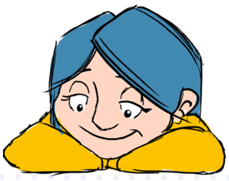

In diesem Workshop entstehen viele Eindrücke und neue Gedanken, die in diesem Gedankenparkplatz bestens aufgehoben sind. Viel Spaß beim Befüllen!

Intro

Warum Patient and Public Involvement & Engagement (PPIE)?

Forschung ohne Patient:innen ist vergleichbar mit einem Puzzle, bei dem ein Teilchen fehlt: Man kann zwar viele Einzelteile betrachten und analysieren, aber ohne das fehlende Stück bleibt das Gesamtbild unvollständig - es fehlt die Gewissheit, ob alle Zusammenhänge verstanden wurden. Es ist daher eine Verbesserung und ermutigend, dass die **Einbindung von Patient:innen** in der klinischen Forschung zunehmend an Bedeutung gewinnt.

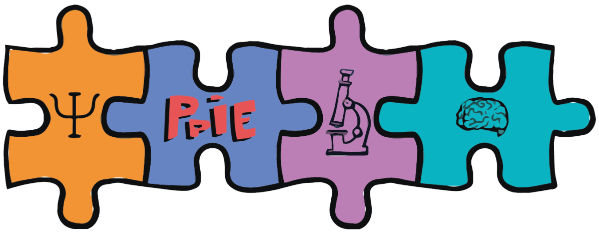

Direkt Betroffene bringen einen realen Erfahrungsschatz mit Behandlungsmethoden sowie ihren Aus- und Nebenwirkungen mit. Oder, um bei der Metapher des Apfels zu bleiben: Sie wissen nicht nur, wie der Apfel aussieht, sondern auch, wie er schmeckt. Zu lange wurde dieses Wissen aus der Praxis wenig berücksichtigt, was nicht nur zu Frustration führt, sondern auch die **Aussagekraft von Studien** schmälert. Und damit die Qualität der erforschten Behandlungsansätze.

Die Antwort auf diese Diskrepanz heißt **"Patient and Public Involvement & Engagement"**, kurz PPIE. Klingt sperrig, ist aber eine kleine Revolution: Das bedeutet, dass Forschung und Entwicklung im Gesundheitsbereich gemeinsam MIT oder sogar VON Patient:innen und Vertreter:innen der Öffentlichkeit durchgeführt werden, nicht ÜBER oder FÜR sie. PPIE steht also für eine Mitsprache und aktive Beteiligung in Forschung & Entwicklung.

- **PPIE** geht über die bisher gängigsten Formen der Partizipation, die hauptsächlich auf Fragebögen, Fokusgruppen und Interviews beruhen, hinaus.
- Bei PPIE werden Patient:innen direkt und von Anfang an **aktiv in Forschungsprozesse involviert:** Von der Formulierung der Forschungsfrage über die Antragstellung für die Finanzierung bis hin zum Sammeln und Auswerten der Daten.
- Die Forschung und die entwickelten Projekte profitieren dank des erweiterten, multidisziplinären Ansatzes von einer **besseren Forschungsqualität**.
- Sie erzielen insgesamt eine **höhere Wirksamkeit**, da bereits von Anfang an die Bedürfnisse von Patient:innen sowie ihr wertvolles Erfahrungswissen berücksichtigt werden.

Im Klartext: Für Forschungsgruppen ist ernsthafte Patient:inneneinbindung das Gebot der Stunde. Dieses Dokument ist eine **praktische Anleitung** dazu. Damit alle Puzzlestücke gemeinsam ein präzises Gesamtbild ergeben.

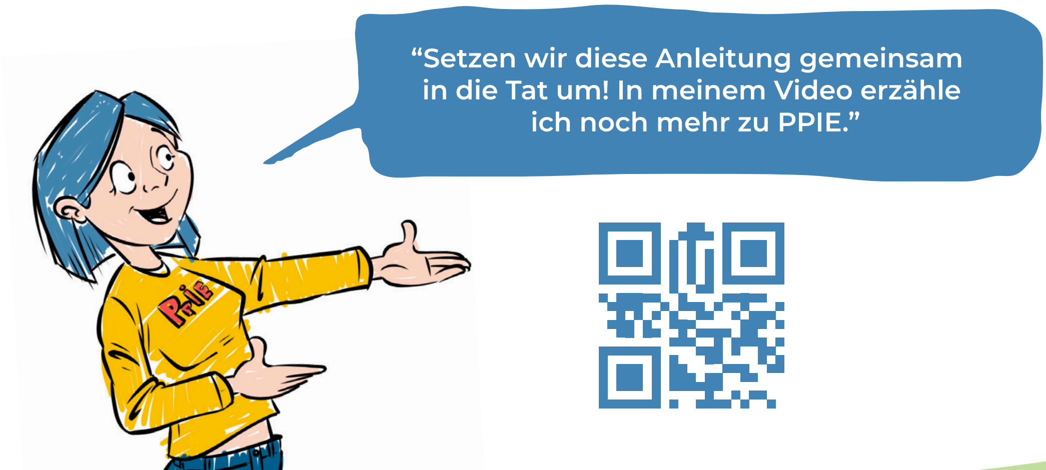

GEDANKENPARKPLATZ

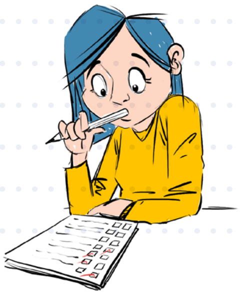

So gelingt die optimale Vorbereitung

Planung ist bekanntlich die halbe Miete – und bringt allen Beteiligten den meisten Spaß bei der Durchführung. Ist erstmal ein Forschungsprojekt gefunden, zu dem Fragen entwickelt werden sollen, geht es an den organisatorischen Klein-(und Groß-) Kram. Passende Co-Moderation und Teilnehmende finden, Methoden und Material vorbereiten, Location für den Workshop sichern, Spielregeln definieren: So gelingt die Vorbereitung.

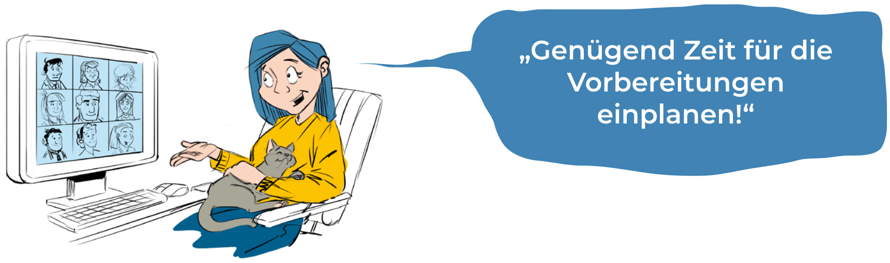

Moderation organisieren

Zu zweit ist besser als allein. Diese Binsenweisheit trifft auch auf diesen Workshop zu:
Eine duale Moderation fördert die Ausgewogenheit in der Gruppe.

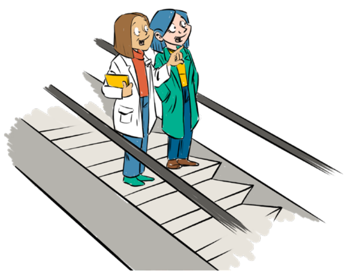

Idealerweise wird der Workshop von einer Person mit der Erfahrung einer eigenen Erkrankung („Patient Expert“) und einer mit Expertise im Gesundheitswesen (“Health Care Professional”) oder mit wissenschaftlichem Hintergrund geleitet. Erfahrung mit der Leitung von Workshops ist empfehlenswert, aber keine Grundvoraussetzung. In diesem Fall spielen die Co-Moderator:innen die Module im Zuge der Vorbereitung gemeinsam durch, bis sich beide in ihrer Rolle sicher und wohl fühlen. Wichtig ist, dass die Co-Moderator:innen in Bezug auf Planung, Durchführung und Evaluierung gemeinsam Entscheidungen treffen

Raum wählen und Logistik prüfen

Kajüte oder Festsaal? Bei der Buchung des Workshop-Raums müssen die **Bedürfnisse der Teilnehmenden** berücksichtigt werden. Der Raum muss gut erreichbar und, wenn notwendig, auch barrierefrei zugänglich sein. Da viel Zeit darin verbracht wird, sollte er über ausreichend Tageslicht und ein gutes Raumklima verfügen.

Logistik vor Ort: Sind genügend Flipcharts vorhanden? Ist ein Beamer da, um eine Präsentation zu zeigen? Sind die richtigen Anschlüsse für meinen Laptop vorhanden, oder ist ein PC vor Ort? Benötige ich Lautsprecher? Da sind einige Fragen, auf die man bei der Raumbuchung achten sollte.

Nicht vergessen: Auch die **Verpflegung** muss vorbereitet werden. Must-have: ausreichend (Mineral-) Wasser für die Teilnehmer:innen. Nice-to-haves: Kaffee, Tee, Milch, Süßstoff, Obst, (gesunde) Snacks etc. Je nach Räumlichkeiten werden die Mahlzeiten vor Ort eingenommen – z.B., wenn man sich in einem Seminarhotel oder einem Restaurant befindet. In diesem Fall müssen die nötigen **Reservierungen** getroffen werden.

Workshop-Vorbereitung

"Schreibmaterial sollte frei zugänglich sein. So können die Teilnehmer:innen jederzeit Notizen machen."

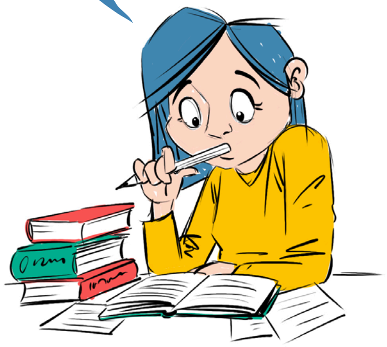

Material & Unterlagen zusammenstellen

Für die gesamte Workshopdauer:

- ☐ Für jede:n Teilnehmer:in ein „Lab-Book"
- ☐ Notizblöcke (mind. A5) & Kugelschreiber für die Teilnehmer:innen
- ☐ Genügend Flipcharts
- ☐ Bunte Stifte (Filzstifte für Flipcharts)
- ☐ Moderationskarten
- ☐ Verschiedenfarbige Haftnotizzettel
- ☐ Klebepunkte und diverse Sticker
- ☐ Klebeband
- ☐ Namensschilder
- ☐ Kleiner Stoffball
- ☐ Optional: Pinnwand & Pins/Reißnägel oder Magnettafel mit Zubehör
- ☐ ____________________
- ☐ ____________________

Tipp: Für Feedbackrunden oder Abstimmungen kann das digitale Tool **Mentimeter** verwendet werden. Dies sollte in der Vorbereitung mitgedacht werden! Website: mentimeter.com.

Für die einzelnen Module:

"Bausteine (z.B. das Set „Lego Classic-Kreativbauset"), Knetmasse, Pfeifenputzer und/oder Bastelmaterial ermöglichen ein bedonderes Workshoperlebnis!"

Modul 1:

- ☐ Flipcharts mit Fragen *(1. Ankommen)*
- ☐ Flipchart mit Agenda & Spielregeln *(3. Agenda & Einführung)*
- ☐ Präsentation PPIE *(3. Agenda & Einführung)*
- ☐ Kärtchen mit Workshop-Rollen *(4. Die Rollen im Workshop)*
- ☐ Avatar-Arbeitsblatt *(5. Avatar erstellen)*

Modul 2:

- ☐ Präsentation Forschungsprozess *(11. Der Forschungsprozess)*
- ☐ Optional: Mini-Präsentation (Richtwert: 2 Slides) zum Forschungsthema *(10. Einführung in das Forschungsthema)*

Modul 3:

- ☐ Erfindungs-Arbeitsblatt *(17. Erfinder:in)*

Modul 4:

- ☐ Präsentation & Arbeitsblätter *(21. Studiendesign-Wegweiser)*
- ☐ ABC-Plakat *(24. Reflexion)*
- ☐ Feedbackbögen oder Online-Survey mit QR-Code *(25. Feedback)*

Zur vollständigen Material- und Unterlagensammlung geht's hier entlang!

Die geeignete Gruppe finden

Um bei der Apfel-Metapher zu bleiben: Das bestmögliche Ergebnis erzielt eine Gruppe, die den Apfel von unterschiedlichsten Perspektiven betrachtet. Ein **ausgeglichenes Verhältnis zwischen Wissenschaft, Medizin bzw. Psychologie und Betroffenen** ist essenziell. Eine ideale Gruppe besteht daher aus Forscher:innen, Ärzt:innen, Psycholog:innen, Pfleger:innen, Patient:innen sowie deren Zugehörigen, die sich im Idealfall nicht kennen. So starten alle Teilnehmer:innen mit der gleichen Basis.

Entgelt

Alle Gruppenmitglieder bringen die gleiche Zeit auf, das gilt auch für Patient:innen. Sie sollen **für ihre Expertise und Erfahrung entlohnt** werden. Der **Fair-Market-Value-Calculator** kann hier Orientierung geben: https://nationalhealthcouncil.org/fair-market-value-calculator/.

Tipp: Die Kosten für den Workshop sollten im Gesamtprojektdesign berücksichtigt und bei Calls entsprechend miteingerechnet und budgetiert werden. Das Einwerben von Fördermitteln ist daher ein relevanter Projektabschnitt.

Verträge

Wer “klinische Forschung” sagt, muss auch “**Einwilligungserklärung**” sagen. Oder „Werkvertrag“ – da es sich auch bei Patient:innen um Expert:innen auf ihrem jeweiligen Gebiet handelt. Jede Einrichtung geht anders mit solchen Verträgen um. Es ist aber unerlässlich, diese vorab aufzusetzen und den rechtlichen Rahmen zu klären, wenn nötig mit der Rechtsabteilung.

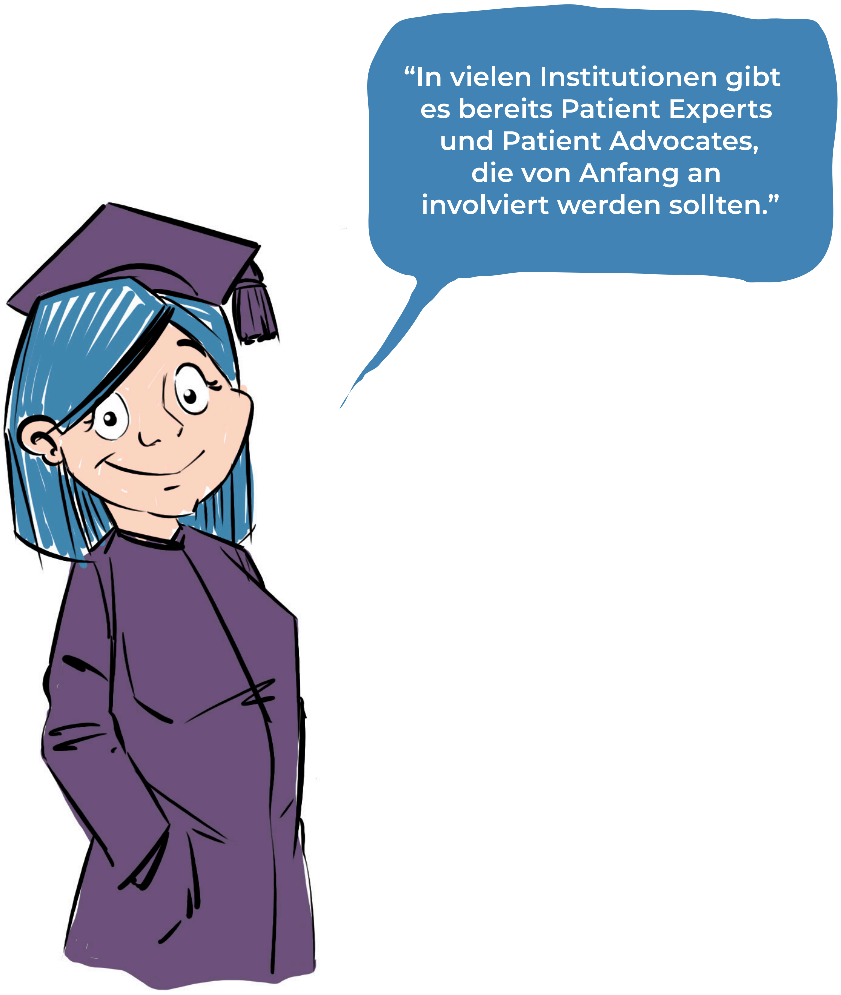

Berücksichtigung besonderer Bedürfnisse

Jedes Teammitglied bringt andere **Perspektiven** und **Expertisen** mit. Dasselbe gilt für die jeweiligen Bedürfnisse. Folgende Überlegungen gehören zu einer **guten Planung**:

- Ist Barrierefreiheit notwendig? Wenn ja: Kann die Workshop-Location barrierefrei erreicht werden? Ebenso die Sanitäreinrichtungen?
- Ist der Raum innerhalb des Gebäudes gut zu finden & ausreichend beschildert?
- Ist die Anwesenheit einer Assistenz erforderlich?
- Kann ausreichend gelüftet werden?
- Welche Lichtverhältnisse herrschen in der Location?
- Wird eine Verpflegung zur Verfügung gestellt (Nüsse, Gebäck, Getränke)?
- Welche Uhrzeiten und Termine sind für alle Beteiligten angenehm?
- Werden Pausen sinnvoll und, wenn notwendig, großzügig eingeplant?

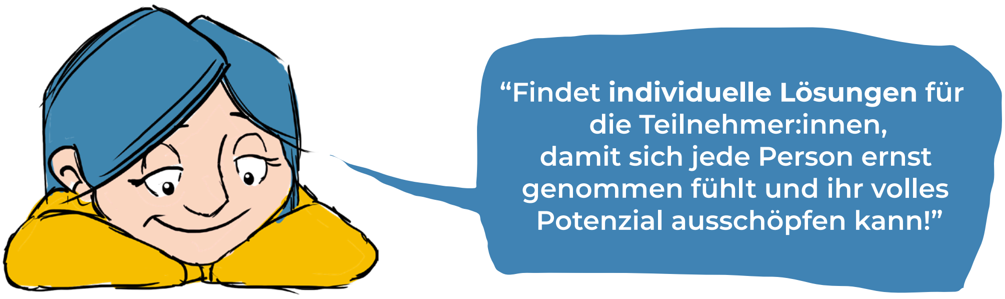

Bei kognitiven Beeinträchtigungen

Erkrankung und Therapie können bei einzelnen Teilnehmer:innen dazu führen, dass es Einschränkungen bei Konzentration, Merkfähigkeit, Ausdauer oder Energiehaushalt kommt. Das darf nicht zum Nachteil werden. Deshalb soll der Workshop darauf abgestimmt sein. Das hilft:

- **Tempo anpassen**: Abläufe verlangsamen und Inhalte regelmäßig zusammenfassen und wiederholen.
- Methoden **mit allen Sinnen** verbinden (Sehen, Hören, Fühlen, Schmecken).
- Das Co-Moderationsteam sollte Teilnehmende regelmäßig dazu animieren, wichtige Inhalte **in ihrem Lab-Book festzuhalten**, sodass sie eigenständig nachgelesen werden können. Das Lab-Book dient als Notiz- und Arbeitsheft und wird am Anfang des Workshops ausgehändigt.

Spielregeln definieren

Gruppe gefunden und Vertragliches geklärt? Sehr gut. Um als Co-Moderationsteam alle Beteiligten gleichermaßen ins Boot zu holen, sollten bereits vor Workshopstart gewisse Spielregeln verinnerlicht werden:

- **Auf Augenhöhe:** Fachbegriffe bewusst nutzen und ggf. erklären. Ein PPIE-Wörterbuch, das die Teilnehmer:innen selbst ergänzen können, bietet sich hier an (siehe Anhang). So können sie ein Gefühl der zusätzlichen Kompetenz und des Selbstvertrauens entwickeln.
- **Per Du:** Für eine gute und wertschätzende Zusammenarbeit bietet sich das Du-Wort und die Ansprache mit dem Vornamen an. Dadurch wird die Atmosphäre gelockert und das Brainstormen und das kreative Arbeiten erleichtert.

10 Gebote für gute Zusammenarbeit

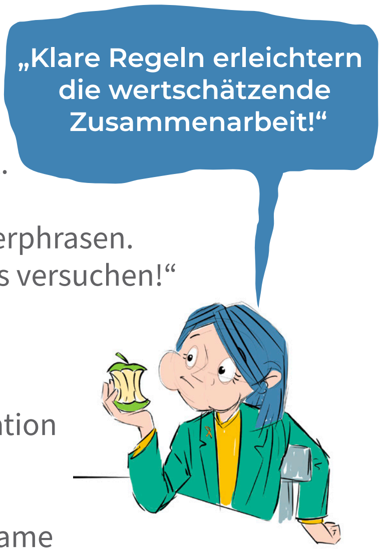

1. „**Lasst euch aufeinander ein**.“ Lasst einander ausreden – jede Stimme soll gehört werden.
2. „**Geht wertschätzend miteinander um**.“ Respektiert einander und schätzt das Wissen, das jede:r miteinbringt und erreicht hat.
3. „**Verwendet eine lösungsorientierte Sprache**.“ Vermeidet Killerphrasen. Statt “Das wird sowieso nicht funktionieren“ lieber „Lasst es uns versuchen!“
4. „**Seid konstruktiv**.“ Bietet Alternativen zu jeder Kritik.
5. „**Ersetzt ‚aber‘ durch ‚und**‘.“ Ein ‚aber‘ wirkt in der Kommunikation negativer als ein “und”; das verändert die Stimmung im Raum.
6. „**Übernehmt Verantwortung**.“ Einigt euch darauf, die gemeinsame Verantwortung für den Erfolg des Workshops zu tragen.
7. „**Seid im Hier und im Jetzt**.“ Schaltet „Ablenker“ wie z.B. Smartphones und Pager auf lautlos.
8. „**Arbeitet ergebnisoffen**.“ PPIE bedeutet, den Mut zu haben, offen zu denken, um Lösungen außerhalb gewohnter Konventionen zu ermöglichen.
9. „**Betrachtet Perspektivenvielfalt als Chance**.“ Der Workshop ist ein „offener Raum“ für Austausch – nutzt diese Chance, redet miteinander, tauscht eure Perspektiven aus und zeigt Bereitschaft, in die jeweils andere Welt einzutauchen.
10. „**Seid zuversichtlich**.“ Der Workshop bedeutet natürlich Zeit und Arbeit. Der PPIE-Prozess ist aber für alle Beteiligten ein echter Gewinn.

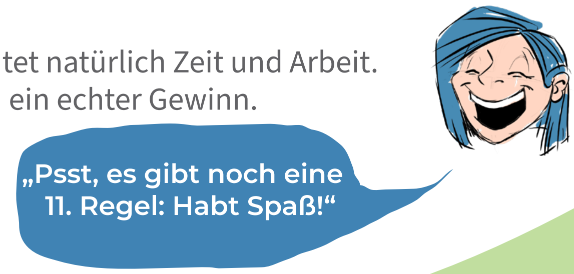

Vorbereitung der Teilnehmer:innen

Je mehr Info, desto besser? Nicht hier. Die **richtige Info** ist das Ziel. Um Teilnehmer:innen vorab nicht mit Informationen zu überladen und trotzdem eine gute gemeinsame Basis zu schaffen, sollte die vorab versendete Info-Mail folgendes enthalten:

- [ ] Zeit und Ort des Workshops
- [ ] Grund des Zusammentreffens (Beschreibung des Forschungsvorhabens)
- [ ] PPIE-Erklärung und Link zum PPIE-Video (Lab-Book – ***siehe Materialsammlung***)
- [ ] Grobe Agenda
- [ ] Abfragen der Bedürfnisse
- [ ] PPIE-Wörterbuch (Lab-Book – ***siehe Materialsammlung***)
- [ ] Infos zu Entgelt und Verträgen

Jede:r Teilnehmer:in erhält im Workshop ein Lab-Book. Es befindet sich in der Materialsammlung und soll vom Co-Moderationsteam ausgedruckt und am Anfang des Workshops verteilt werden. Das Lab-Book kann auch bereits vorab geschickt werden – es enthält neben einer **PPIE-Erklärung** auch das **PPIE-Wörterbuch**, das selbst erweitert werden kann, sowie genügend Platz für **eigene Notizen**.

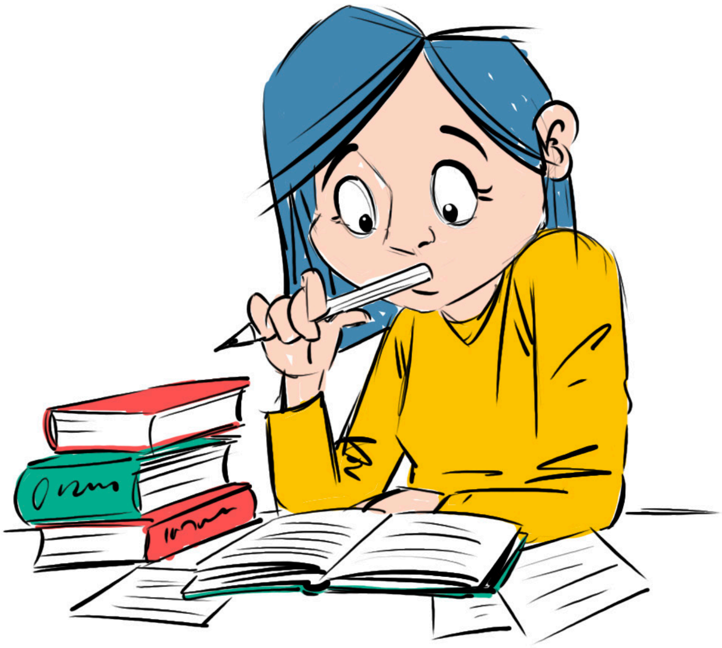

Workshop-Übersicht

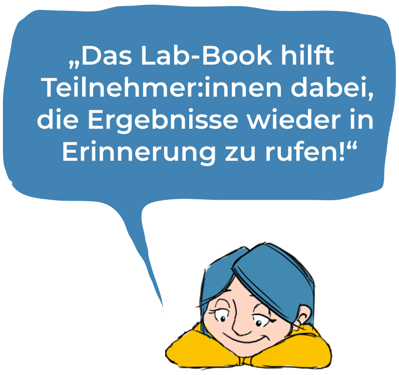

Der Workshop

Roadmap zu den Modulen 1-4

Jetzt, wo alles vorbereitet ist, fängt der beste Teil an: **die Durchführung**. Damit die hier vorgestellten Methoden zum gewünschten Ziel führen, bietet sich ein Workshop-Zeitraum von zweimal eineinhalb Tagen und eine Einteilung in vier Module an. Hier ein Beispiel:

Modul 1: Freitag von 13:00-16:30
Modul 2: Samstag von 09:30-16:00
Modul 3: Freitag von 13:00-17:00
Modul 4: Samstag von 09:30-16:00

Wir empfehlen, jeweils zwei Module auf ein Wochenende zu legen, ideal sind Freitag und Samstag. So kann die Gruppe auch abends sozialen Kontakt pflegen, was den Zusammenhalt stärkt. Es hat sich bewährt, die **Module 3 und 4 zu einem späteren Zeitpunkt** gesondert durchzuführen, z.B. einen Monat später. Aufeinanderfolgende Workshop-Wochenenden machen müde, und neue Ideen brauchen oft ein wenig Zeit, um zu wachsen. Der Zeitraum zwischen den beiden Workshop-Wochenenden sollte allerdings nicht zu lang sein, damit die erarbeiteten Ergebnisse noch halbwegs präsent sind.

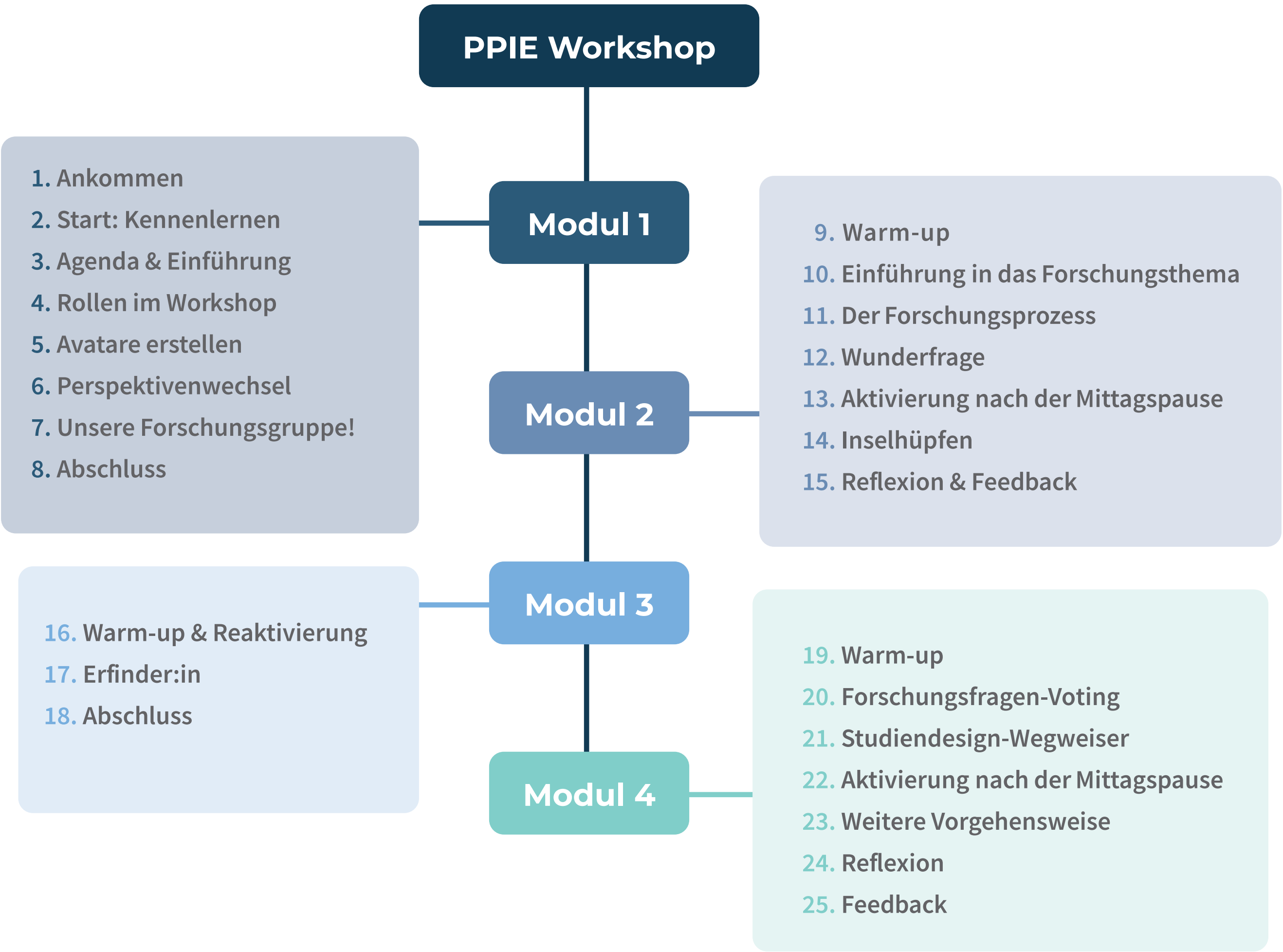

GEDANKENPARKPLATZ

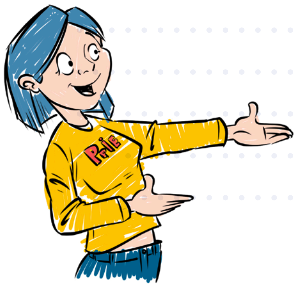

Der Einstieg

Vorbereiten für Modul 1:

- ☐ Flipcharts mit Fragen *(1. Ankommen)*
- ☐ Flipchart mit Agenda & Spielregeln *(3. Agenda & Einführung)*
- ☐ Präsentation PPIE *(3. Agenda & Einführung)* - ***siehe Materialsammlung***
- ☐ Kärtchen mit Workshop-Rollen *(4. Die Rollen im Workshop)*
- ☐ Avatar-Arbeitsblatt *(5. Avatar erstellen)* - ***siehe Materialsammlung***

Ziele des Moduls: Kennenlernen & Gruppenidentität finden

Los geht's! Am Anfang steht das Kennenlernen. Und zwar "auf Augenhöhe": Alle bringen verschiedene Expertisen mit, die für die Forschung und für den Workshop unverzichtbar sind. Die gewählten Methoden stellen sicher, dass jede:r Teilnehmer:in mit den persönlichen Stärken einen Platz in der (Forschungs-)Gruppe findet. So wird die Basis für die Zusammenarbeit gelegt – im Workshop und darüber hinaus.

Dauer: ca. 3,5 Stunden (z.B. Freitag 13:00-16:30)

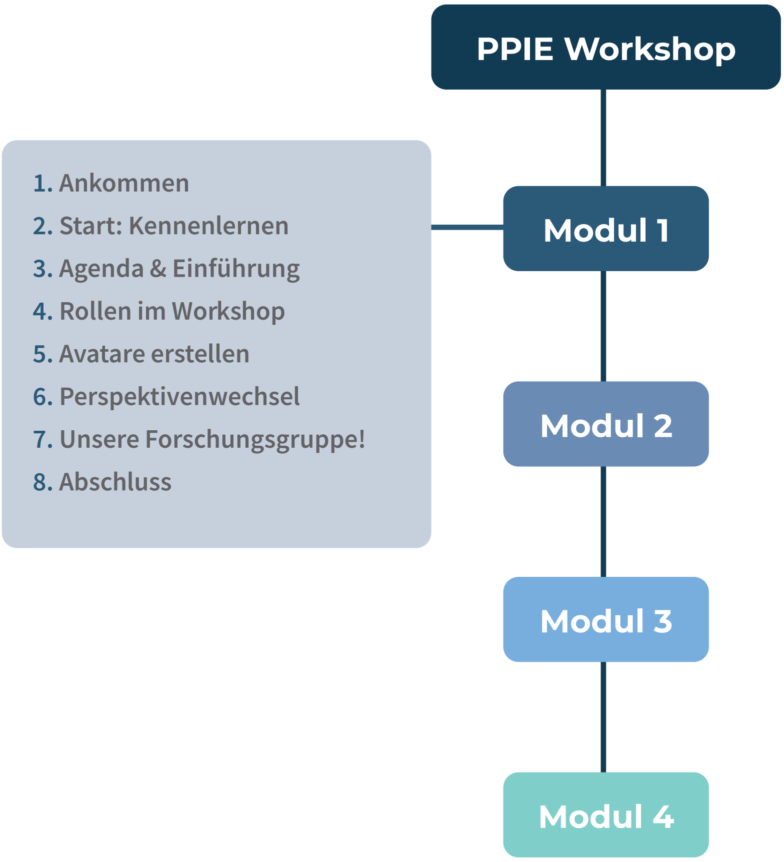

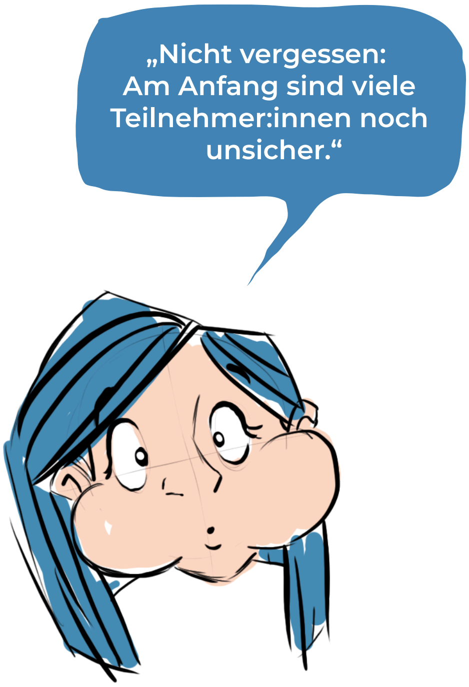

Methode 1: Ankommen
3 Fragen, 3 Wände

Warum? Das Ankommen im Workshop erleichtern + erste inhaltliche Auseinandersetzung mit dem Thema.

Dauer? Ab dem Eintreffen der ersten Teilnehmer:innen bis zum Start (ca. 10 Minuten).

Material:

- 3 Flipcharts mit Fragen
- Stifte
- Klebepunkte

Beschreibung

Verlegene Blicke, nervöses Schweigen, Schweißperlen auf der Stirn: Das Ankommen in einem Workshop ist oft etwas unangenehm. Um den Teilnehmer:innen diese Phase zu erleichtern, werden drei Plakate mit jeweils einer Frage aufgehängt. Das Co-Moderationsteam begrüßt die Eintreffenden persönlich und fordert sie auf, von Plakat zu Plakat zu gehen. Mit Stiften können sie ihre Antworten zu den Fragen schreiben oder eine vorformulierte Antwort mit einem Klebepunkt markieren. Um die Atmosphäre aufzulockern, kann leise Musik gespielt werden. Die Antworten auf den Plakaten können später in den Workshop integriert werden, z.B. durch Bezugnahme bei der Einführung.

Mögliche Fragen für die Flipcharts:

„Hast du schon einmal von “Patient & Public Involvement and Engagement” gehört?”

(Vorformuliert: Ja/Nein)

„Hattest du schon einmal Berührung mit Forschung?”

(Vorformuliert: Ja/Nein)

„Was erwartest du von diesem Workshop?”

(Antworten dazuschreiben lassen)

Methode 2: Der Start: Kennenlernen

Raumaufstellung

Warum? Die Teilnehmer:innen lernen sich kennen.

Dauer? 10 Minuten

Beschreibung

Ein klassischer Workshop-Einstieg, der Spaß macht! Nach einem kurzen "Hallo" geht es gleich los: Die Teilnehmer:innen stellen sich als Antwort auf Fragen des Co-Moderationsteams im Raum auf. Wie wäre es beispielsweise mit einer Aufstellung nach Geburtsort auf einer fiktiven Landkarte? In einer kurzen Auflösungsrunde erzählen die Teilnehmer:innen, warum sie sich so platziert haben. Bereits bei der Suche nach dem eigenen Platz findet eine erste Kommunikation miteinander statt, die das erste Kennenlernen fördert. Der Prozess sollte sich dreimal wiederholen.

Besonders empfehlenswerte Aufgaben:

„Wo wurdest du geboren?“

(Aufstellung auf fiktiver Landkarte)

„Wie intensiv hast du dich bisher mit dem Thema des Workshops beschäftigt?“

(Skalenaufstellung zwischen “sehr” und “gar nicht”)

„Wie viel Zeit hast du in letzter Zeit in medizinischen Einrichtungen verbracht?“

(Skalenaufstellung von “wenig“ bis “viel”)

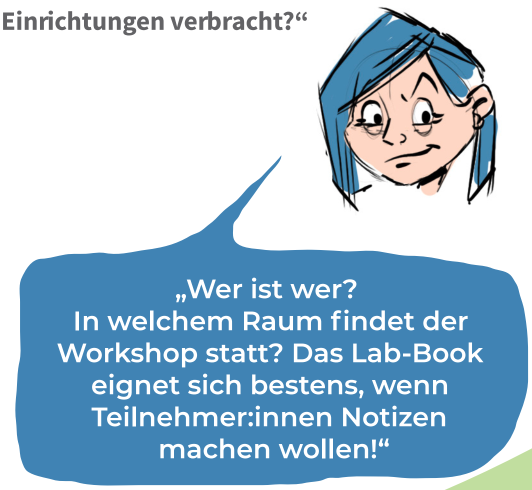

Methode 3: Agenda & Einführung

Vortrag & Diskussion

Warum? Orientierung, Spielregeln, Wissensstand angleichen.

Dauer? 30 Minuten

Material:

- Vorbereitetes Flipchart
- Powerpoint

Beschreibung

1. Willkommen & Agenda:

Jetzt hat die Gruppe sich kurz kennengelernt. Zeit, alle willkommen zu heißen und die Agenda vorzustellen! Zunächst erklärt das Co-Moderationsteam in wenigen Sätzen die **Ziele des Workshops und des Projekts**. Der Ablauf der nächsten eineinhalb Tage wird kurz vorgestellt. Die wichtigsten Punkte stehen auf der Agenda, die zuvor auf einem Flipchart festgehalten wurde. Nicht zu detailliert.

Tipp: Die Einführung kann kreativ gestaltet werden, z.B. anhand eines Beispiels, einer Geschichte oder bezugnehmend auf die Flipcharts, die die Teilnehmer:innen beim Ankommen befüllt haben.
Dauer: ca. 10 Minuten.

2. Spielregeln:

Ablauf klar? Dann geht es jetzt ums Zusammenarbeiten.
Wie, das wird bei der Vorstellung der Spielregeln beantwortet.
Diese werden ebenfalls auf ein Flipchart geschrieben (z.B. am Agenda-Chart).
Dauer: ca. 5 Minuten.

„Auch Teilnehmer:innen zu Wort kommen lassen!“

3. Einführung:

Was ist PPIE? Das Co-Moderationsteam gibt diese Frage gleich an die Teilnehmer:innen weiter – was wissen sie bereits über “Patient & Public Involvement & Engagement”? Kurze Plenumsdiskussion. Dann werden anhand von 3 Powerpoint-Slides kurz und prägnant die wichtigsten Eckpfeiler erklärt und alle Anwesenden auf denselben Wissensstand gebracht.
Dauer: ca. 15 Minuten.

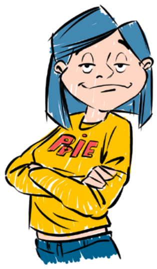

Methode 4: Die Rollen im Workshop

Kurzer Impuls

Warum? Einbindung, Aufgabenverteilung.

Dauer? 10 Minuten

Material:

- Kärtchen oder Haftnotizzettel

Beschreibung

Vor dem inhaltlichen Start des Workshops vergeben die Moderator:innen Rollen an Freiwillige. Warum? Auf diese Weise wird der Workshop zu einem gemeinsamen Vorhaben mit verteilter Verantwortung, was zugleich eine Entlastung für das Co-Moderationsteam bedeutet. Folgende Rollen werden vorab je auf ein Kärtchen geschrieben, anschließend erklärt und vergeben. Die Vergabe sollte nicht todernst sein, sondern kann humorvoll gestaltet werden (z.B. wie auf einem Jahrmarkt!).

Folgende Rollen werden vorab je auf ein Kärtchen geschrieben, anschließend erklärt und vergeben:

Zeitwächter:in

Achtet darauf, dass der Zeitrahmen nicht gesprengt wird.

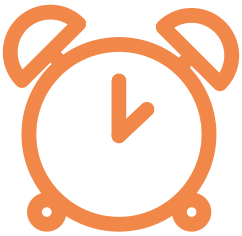

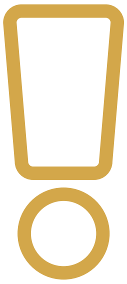

Aber-Wächter:in

Mischt sich ein, wenn jemand in einer Diskussion „aber" sagt und verwandelt dieses in ein "und".

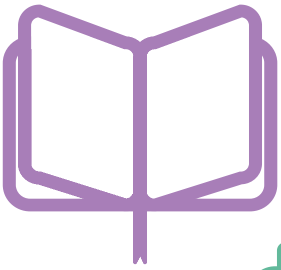

Wandelndes Wörterbuch

Notiert Fachbegriffe und Erklärungen auf einem Chart. Erinnert die Gruppe daran, Fachbegriffe zu erklären, wenn sie es vergessen.

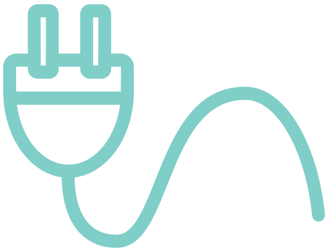

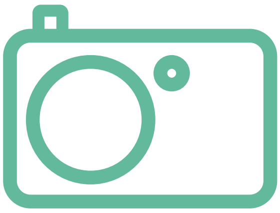

Ladegerät

Achtet auf das Energie-Level im Raum und fordert Pausen ein, wenn es zu niedrig ist.

Fotograf:in

Fotografiert die Ergebnisse, macht Bilder von der Gruppe und übermittelt diese im Nachgang an das Co-Moderationsteam.

Methode 5: Avatare erstellen

Avatare kreieren

Warum? Darstellen von Stärken, Kenntnissen, Expertisen.

Dauer? 1,5 Stunden

Material:

- Bausteine (*z.B. Lego Classic-Set)*
- eventuell Pfeifenputzer
- Knetmasse
- Avatar-Arbeitsblätter *(siehe Materialsammlung)*

Beschreibung

Jetzt ist Handarbeit gefragt! Das Arbeiten mit den Händen löst kreative Prozesse im Kopf aus und ermöglicht freies Denken. Die verschiedenen Kreationen können wunderbar als Metaphern eingesetzt werden. Diese Übung gliedert sich in vier Bereiche:

1. Vorbereiten:
Zu Beginn erhalten alle Teilnehmer:innen Bausteine, Pfeifenputzer oder Knetmasse. Oder all das. Die Materialien liegen auf Tischen im Raum oder werden den Teilnehmenden direkt ausgehändigt. Wichtig: Der Kreativität sind keine Grenzen gesetzt, es gibt kein richtig oder falsch! Das betont das Co-Moderationsteam gleich zu Beginn.
Dauer: 10 Minuten.

2. Bauen:
Mithilfe der Materialien bauen die Teilnehmer:innen nun ihren Avatar: also eine Figur, die sie repräsentiert. Der Avatar soll die eigenen Stärken, Kenntnisse und Expertisen symbolisieren.
Dauer: 15 Minuten.

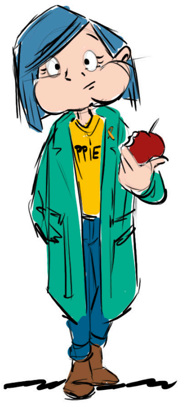

3. Beschreiben:
Die Teilnehmer:innen nehmen nun ihr Avatar-Arbeitsblatt zur Hand.
Darauf beschreiben sie ihren Avatar:

- Welche Stärken hast du?
- Welche Expertisen bringst du mit?
- Welche besonderen Eigenschaften hast du?

Dauer: ca. 20 Minuten.

„Nicht vergessen, die Avatare auf die Arbeitsblätter zu stellen und zu fotografieren! Die Co-Moderator:innen können auch selbst mitbasteln.“

20 Minuten Pause.

4. Teilen:
Nun stellen die Teilnehmer:innen einander ihre Avatare samt Stärken, Kenntnissen und Expertisen vor. Jede:r Teilnehmer:in bekommt dafür die gleiche Zeit!
Dauer: 30 Minuten.

Methode 6: Perspektivenwechsel
Hineinversetzen in Stakeholder-Rollen

Warum? Sichtbarmachen von Kompetenzen.

Dauer? 40 Minuten

Material:

- Flipcharts
- Stifte

Beschreibung

Dank der Übung mit den Avataren kennen nun alle im Raum die persönlichen Stärken der anderen Teilnehmer:innen. Super! Im nächsten Schritt geht es nun darum, sich in die verschiedenen **Rollen der Projektbeteiligten** zu versetzen. Dazu werden an vier Orten im Raum leere Flipcharts aufgestellt, jedes Plakat steht für eine:n Stakeholder:in des Forschungsprojekts. Also eine Personengruppe, die davon in irgendeiner Weise betroffen oder darin involviert ist: Patient:innen, Forschende, Ärzt:innen, Pflegefachkräfte.
Dauer: ca. 10 Minuten.

Die Teilnehmer:innen werden in **vier Gruppen** eingeteilt. Etwa, indem man von 1 bis 4 durchzählen lässt. Jede Zahl bildet eine eigene Kleingruppe (es sollte auf eine gute Durchmischung geachtet werden). Die Kleingruppen verteilen sich auf die Plakate und besprechen die vorgegebenen Fragen und halten die Antworten auf dem Flipchart fest:

- Welche **Kompetenz** bringt die Rolle mit in den Forschungsprozess?
- **Was** muss passieren, damit das Projekt aus ihrer Sicht erfolgreich ist?
- Welchen **Wunsch** hat sie an das Forschungsteam?

Dauer: ca. 15 Minuten.

Abschließend präsentiert jede Gruppe kurz “ihre” Perspektive.
Dauer: ca. 15 Minuten.

Methode 7: Unsere Forschungsgruppe
Plenumsdiskussion

Warum? Gruppenidentität stärken.

Dauer? 20 Minuten

Material:

- Flipchart

Beschreibung

Zeit für eine Zusammenfassung. Das Co-Moderationsteam „präsentiert“ der Gruppe, welche Stärken ihre Mitglieder haben. Diese wurden bereits bei der Avatar-Erstellung sichtbar gemacht. In einer knapp gehaltenen Gruppendiskussion wird nun die **neue Forschungsgruppe** definiert, welche die Teilnehmer:innen fortan gemeinsam bilden. Sie können auch mehrere kleinere Gruppen bilden. Als Orientierung werden folgende Punkte aufgeschrieben, besprochen und schriftlich auf einem Plakat festgehalten:

- **Was ist unser Ziel als Forschungsgruppe?**
- **Welche Stärken** sind vorhanden?
- **Welche Kenntnisse** bringen wir alle mit?
- **Was zeichnet** uns als Gruppe aus?
- **Welchen Namen** geben wir unserer Forschungsgruppe?
- Was ist unser **Logo/Symbol?**

8. Abschluss
Blitzlicht

Warum? Abschluss, Reflexion.

Dauer? 5-10 Minuten

„Notizen zur Forschungsgruppe auch ins Lab-Book eintragen lassen!“

Beschreibung

Geschafft! Das erste Modul ist zu Ende. Die Gruppe hat sich gefunden, alle Stärken liegen auf dem Tisch, das Team hat sogar einen Namen. Bevor der erste Workshoptag beendet wird, sollte kurz die Stimmung der Gruppe eingefangen und die **Möglichkeit für Feedback** gegeben werden. Das Co-Moderationsteam bittet jede:n um einen Satz, wie es der Person nach dem ersten Modul geht. Auf die Wortmeldungen soll nicht geantwortet werden. Auch das Co-Moderationsteam beteiligt sich.

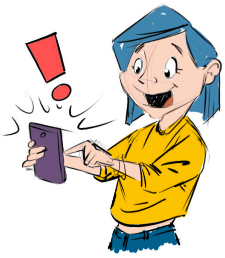

Anschließend Verabschiedung und evtl. **organisatorische Hinweise** zum Thema Abendessen und Unterkunft.

Forschungsfragen finden

Vorbereiten für Modul 2:

- ☐ Präsentation Forschungsprozess *(11. Der Forschungsprozess)* - ***siehe Materialsammlung***

- ☐ Optional: Mini-Präsentation *(Richtwert: 2 Slides)* zum Forschungsthema *(10. Einführung in das Forschungsthema)*

Ziele des Moduls: Forschungsfragen finden

Schönen gemeinsamen Abend verbracht und ausreichend erholt? Gut! Dann kann nun die gemeinsame Arbeit am Forschungsthema beginnen. Die geplanten Methoden ermöglichen ein kreatives Entwickeln von ersten Forschungsfragen.

Dauer: 7,5 Stunden (z.B. Samstag 9:30-16:00)

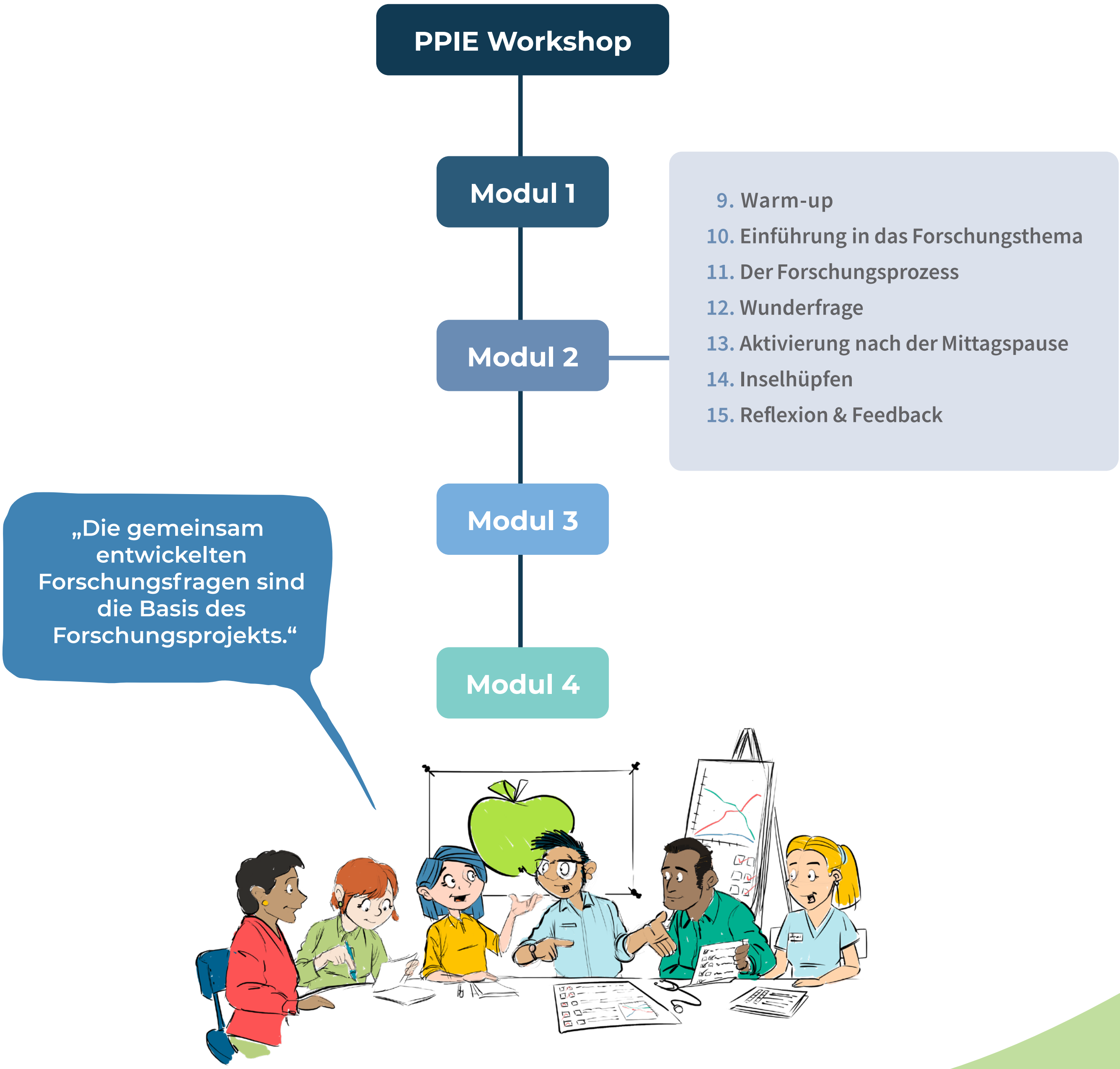

Methode 9: Warm-Up
Zwei Wahrheiten - eine Lüge

Warum? Aktivierung & vertiefendes Kennenlernen.

Dauer? 20 Minuten

Beschreibung

Die Teilnehmer:innen kennen sich nach dem ersten Tag schon ein wenig. Aber wie gut wirklich? Das finden sie bei diesem lustigen Aktivierungsspiel heraus: Jede:r überlegt sich **zwei Wahrheiten und eine Lüge** über sich selbst. Es darf etwas Lustiges oder Privates sein oder einen Bezug zum Thema haben. Die anderen müssen nun erraten, welches Statement die Lüge ist. Um das Tempo zu erhöhen, kann die jeweils zuletzt am Zug gewesene Person die nächste bestimmen.

Methode 10: Einführung ins Forschungsthema
Impulsvortrag des Co-Moderationsteams

Warum? Gleichen Wissensstand zum Forschungsthema herstellen

Dauer? 15 Minuten

Material:

- Mini-Präsentation zum Forschungsthema *(optional)*

Beschreibung

Jetzt geht es inhaltlich los: Bislang ging es vorrangig um das Kennenlernen und das Teambuilding der Forschungsgruppe, nun sollen alle Teilnehmenden auf einen ähnlichen Wissenstand der Grundthematik gebracht werden, die den Grund für das Zusammentreffen darstellt. Diese Fragen sollten dabei beantwortet werden:

- ? Was ist der **Themenbereich** (Psychologie, Medizin, Neuropsychologie, Pharmakologie, soziale Arbeit)?
- ? **Was genau** soll erforscht oder entwickelt werden?
- ? Wie trägt der **Workshop** dazu bei?
- ? Welche Rolle spielt die **Workshopgruppe** im Projekt?

Methode 11: Der Forschungsprozess
Brainstorming, Impuls-Vortrag, freies Assoziieren

Warum? Kennenlernen des Forschungsprozesses und der eigenen Berührungspunkte damit.

Dauer? 1 Stunde

Material:

- Haftnotizzettel
- Flipchart
- Bunte Klebepunkte
- Vorlage Forschungsprozess *(siehe Materialsammlung)*
- *Optional*: Präsentation

Beschreibung

1. Brainstorming:
Die Teilnehmer:innen überlegen, welche Berührungspunkte sie bereits mit Forschung hatten. Jeder ihrer Berührungspunkte soll auf einem Haftnotizzettel aufgeschrieben und gesammelt werden – diese werden in Schritt 3 weiterverwendet.
Dauer: 15 Minuten.

2. Impulsvortrag:
Das Co-Moderationsteam gibt nun einen kurzen Überblick über den Forschungsprozess. Dies kann mithilfe eines Plakats oder einer Präsentation erfolgen. Zur Orientierung dient auch der dargestellte Forschungsprozess (siehe Materialsammlung).
Dauer: 15 Minuten.

3. Freies Assoziieren:
Nachdem die Teilnehmer:innen nun gut über den Ablauf eines Forschungsprozesses informiert sind, bringen sie auf einem oder mehreren Flipcharts ihre zuvor gesammelten Berührungspunkte (Haftnotizzettel) angelehnt an den Forschungsprozess in eine chronologische Reihenfolge. Hier darf und soll miteinander gearbeitet werden! Ergänzungen, die ihnen nach dem Impulsvortrag eingefallen sind, sind erwünscht und sollen ebenso mit einem Haftnotizzettel am Flipchart angebracht werden.
Dauer: 25 Minuten.

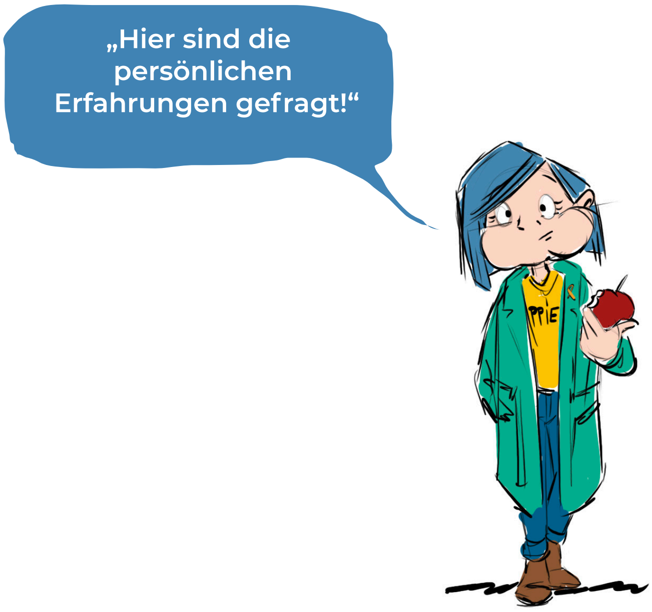

20 Minuten Pause

Methode 12: Die Wunderfrage
Wunderfrage: Was wäre, wenn?

Warum? Kreativität und Optimismus aktivieren.

Dauer? 45 Minuten

Material:

- Kärtchen oder Haftnotizzettel
- Flipchart
- Stifte
- Lab-Book *(optional)*

Beschreibung

Jetzt wird's wieder kreativ! Die Teilnehmer:innen überlegen in einem Brainstorming Lösungsansätze für die grundlegende Forschungsproblematik des Projekts. Dafür stellt das Co-Moderationsteam folgende Frage: "Stell dir vor, du wachst auf und **das Problem ist gelöst.** Woran würdest du das erkennen?"

Um die Frage zu verdeutlichen, können beispielhaft Situationen geschildert werden: Ein:e Patient:in wacht geheilt auf, ein:e Forscherin hat eine knifflige Forschungsfrage gelöst, ein Patient Advocate konnte einen verbesserten Behandlungsansatz im Sinne der Patient:innen erwirken.
Dauer: 5 Minuten.

Jede:r Teilnehmer:in überlegt nun für sich **Antworten** auf folgende drei Fragen und notiert sie auf Kärtchen oder Haftnotizzettel. (Dauer: 10 Minuten):

- **Woran** würdest du bemerken, dass dieses Wunder geschehen ist?
- **Welche** Probleme/Schwierigkeiten wären dadurch verschwunden?
- **Wer** würde sonst noch erkennen, dass dieses Wunder geschehen ist und dass deine Probleme verschwunden sind?

Anschließend werden die Ergebnisse **im Plenum** einzeln vorgestellt und auf ein Flipchart geklebt oder geschrieben. **Die Teilnehmer:innen können ihre Gedanken auch in ihr Lab-Book übertragen.**
Dauer: 30 Minuten.

1 Stunde Mittagpause

Methode 13: Aktivierung nach der Mittagspause

Zip-Zap-Boing

Warum? Aktivieren nach der Mittagspause.

Dauer? 5-10 Minuten

Beschreibung

Gut gegessen? Hoffentlich! Nach dem Mittagsmahl ist es wichtig, das Gehirn wieder kurz zu reaktivieren. Das witzige Spiel “Zip-Zap-Boing“ ist dafür bestens geeignet.

Die Gruppenmitglieder stellen sich in einen Kreis. Es geht ein **Impuls durch die Gruppe**. Er wird nach links weitergegeben, indem man sich nach links wendet, in die Hände klatscht und laut “**Zip**” sagt. Nach rechts gibt man den Impuls weiter, indem man sich nach rechts wendet, in die Hände klatscht und “**Zap**” sagt. Schließlich gibt es noch das “**Boing**”-Kommando: Damit spielt man den Impuls an eine:n beliebige:n Mitspieler:in im Kreis zu (nicht jedoch den direkten Nachbar:innen). Dazu schaut man die Person an, macht mit seinen Händen eine Bewegung, als wolle man einen unsichtbaren Volleyball zu ihr hinüber pritschen, und sagt laut und deutlich “Boing”.

Sobald der Impuls eine:n Mitspieler:in erreicht, kann diese:r sich aussuchen, mit welchem der drei Kommandos sie oder er den Impuls weitergeben möchte. **Ziel des Spiels** ist es, den Impuls so schnell wie möglich umherwandern zu lassen, ohne, dass jemand einen Fehler macht.

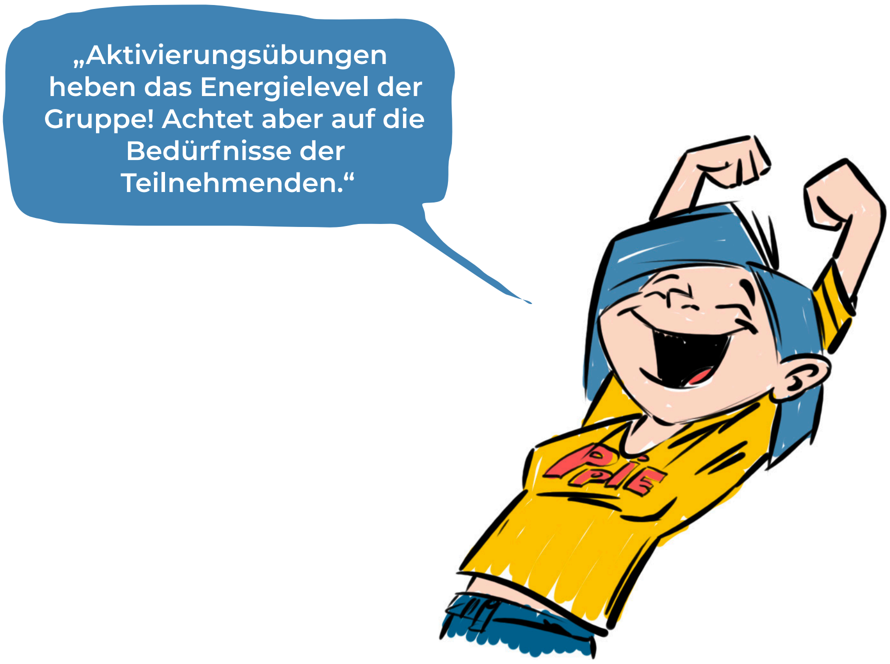

Methode 14: Inselhüpfen
Inselhüpfen bzw. World Café

Warum? Die Teilnehmer:innen formulieren gemeinsam 3 Forschungsfragen.

Dauer? 2,5 Stunden

Material:

- Kärtchen
- Flipcharts
- Stifte
- Pinnwand und Pins *(optional)*

Beschreibung

Jetzt wird es ernst. Man könnte sagen, dass alle bisherigen Schritte die Gruppe auf diese Übung vorbereitet haben: das gemeinsame Ausarbeiten von Forschungsfragen. Deshalb sollte für diese Übung genügend Zeit eingeplant werden.

Schritt 1:

Die Teilnehmer:innen überlegen einzeln, welche konkrete Fragen sie zum Forschungsvorhaben haben und schreiben diese auf Kärtchen. Diese Kärtchen werden im Plenum kurz vorgestellt und dann auf eine Pinnwand gepinnt. Dort findet unter Anleitung des Co-Moderationsteams ein Zusammenfassen in Themen statt (z.B. „Nebenwirkungen", „Lebensqualität", „Diagnosephase", „Therapie", „Reha" oder „Nachsorge" etc.).
Dauer: ca. 40 Minuten.

Schritt 2:

Aus den Themenclustern werden, z.B. per Voting, 3-4 ausgesucht. Dabei muss das Co-Moderationsteam auf Ausgeglichenheit zwischen der Sicht von Health Care Professionals und Patient:innen achten. Die Themen werden auf Plakate geschrieben und im Raum verteilt, wie Inseln im Meer. Pro Plakat wird eine Person ausgesucht, die das Plakat "betreut". Sie sind die Inselbesitzer:innen.
Dauer: ca. 10 Minuten.

Schritt 3:

Die restlichen Teilnehmer:innen werden nun in Kleingruppen aufgeteilt und gehen von Insel zu Insel (pro Plakat ca. 10-15 Minuten). Bei jeder Station formulieren sie Fragen und Überlegungen zum jeweiligen Thema. Die:der Inselbesitzer:in schreibt mit und gibt anschließend auch der nächsten Gruppe eine kurze Zusammenfassung. Achtung: Bei der Gruppeneinteilung ist auf eine gute Mischung der Teilnehmenden zu achten!
Dauer: 50-60 Minuten.

20 Minuten Pause

„Genügend Zeit einplanen und Diskussionen zulassen!"

Schritt 4:

Die Inselbesitzer:innen präsentieren nun ihr jeweiliges Plakat bzw. die Fragen und Anmerkungen zu ihren Themen im Plenum. Unter Anleitung des Co-Moderationsteams votet das Plenum für drei dieser Fragestellungen, die aus ihrer Sicht am relevantesten für das konkrete Forschungsvorhaben sind. Dies sind nun die Forschungsfragen, mit denen weitergearbeitet wird. Die anderen Fragen können auf einem Ideenparkplatz "geparkt" werden. Dafür eignet sich ein neues Flipchart.
Dauer: 30 Minuten.

Methode 15: Reflexion & Feedback
Zugabteil

Warum? Die Teilnehmenden setzen sich aktiv mit dem Erarbeiteten auseinander. Das Verbalisieren dieser Erfahrungen unterstützt den Praxistransfer.

Dauer? 20-30 Minuten

Material:

- 6 Sessel

Beschreibung

Geschafft! Am Ende des zweiten Tages sind die Workshop-Teilnehmenden und das Co-Moderationsteam zufrieden: Sie haben gemeinsam viel geschafft! Bevor alle nach Hause gehen, ist aber noch Zeit für eine Reflexionsrunde.

Das Co-Moderationsteam stellt dafür sechs Sessel **gegenüber voneinander** auf. So, als würden sie sich in einem Zugabteil befinden. Die Teilnehmenden steigen nach Belieben in das „Zugabteil" ein, wenn Plätze frei sind, und unterhalten sich ungezwungen mit den anderen „Reisenden" darüber, wie sie den Workshop bisher fanden, was sie gerne loswerden möchten, was ihnen in besonderer Erinnerung ist etc.

Zuletzt steigen die **Co-Moderator:innen** als Schaffner:innen zu und geben ebenfalls ein kurzes abschließendes Feedback an die Gruppe.

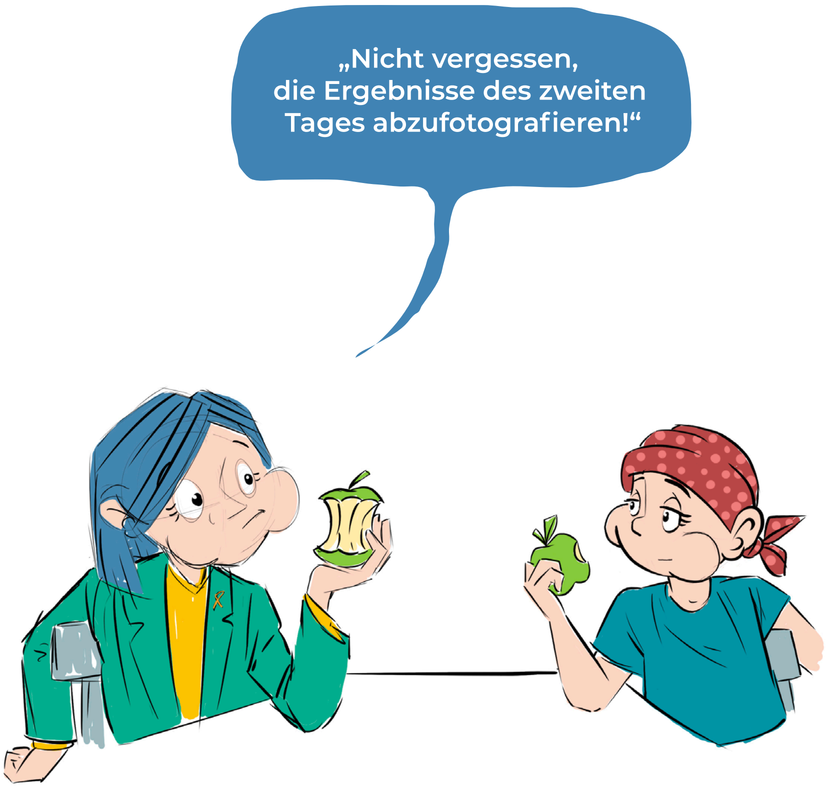

GEDANKENPARKPLATZ

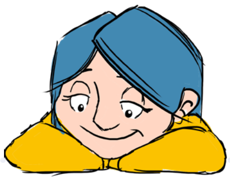

Kreative Lösungen finden

Vorbereiten für Modul 3:

- ☐ Erfindungs-Arbeitsblatt *(17. Erfinder:in)* - ***siehe Materialsammlung***

Ziel des Moduls: Lösungsansätze entwickeln

Endlich geht es weiter! Ein paar Wochen sind vergangen und nach dem positiven Workshoperlebnis während der ersten beiden Module freuen sich die Teilnehmer:innen auf die Fortsetzung. Die Forschungsfragen sind formuliert, jetzt wird die Gruppe kreativ: sie entwickelt „**Problemlöser**“. Um den Wiedereinstieg zu erleichtern, ist eine **vorbereitete Zusammenfassung** der bisherigen Ergebnisse wichtig.

Dauer: Ca. 4 Stunden (z.B. von 13:00-17:00)

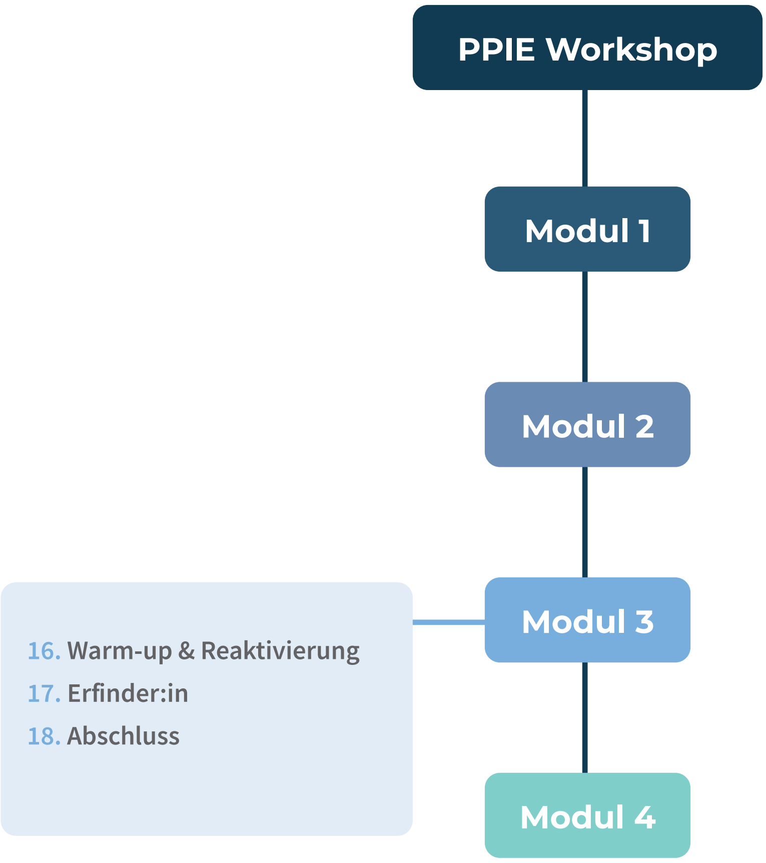

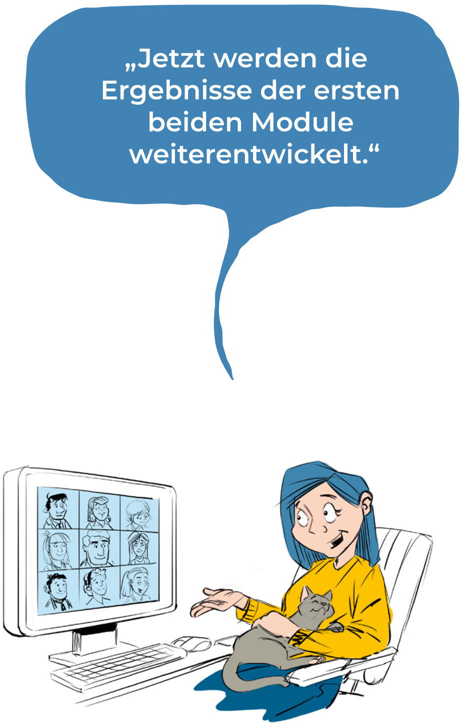

Methode 16: Warm-Up & Reaktivierung
Speed-Dating & Think-Pair-Share

Warum? Reaktivierung der Gruppe, Wiederholung der bisherigen Inhalte.

Dauer? 45 Minuten

Material:

- Kärtchen oder Notizblöcke

Beschreibung

Alle gut angekommen? Dann starten wir. Und zwar mit **einer Wiederholung des bisherigen Workshops.** Die Übung besteht aus drei Schritten:

1. Schritt

Zuerst findet ein kurzes “**Speed-Dating**” statt. Die Teilnehmer:innen werden in Paare aufgeteilt, zum Beispiel, indem man sie durchzählen lässt. Jedes Paar stellt sich je fünf Minuten lang abwechselnd Fragen, um das Wissen und die Erkenntnisse der ersten beiden Module zu besprechen. Mögliche Fragen: „Was waren die wichtigsten Erkenntnisse des ersten Workshopteils für dich?“ oder „Welche Fragen sind noch offen?“
Dauer: 10-15 Minuten.

2. Schritt

Nun wechseln die Paare und der zweite Teil startet, die sogenannte **Think-Pair-Share-Methode**. Die Teilnehmer:innen setzen sich mit einer anderen Person zusammen, um ihre Gedanken und Erkenntnisse auszutauschen und in Form von Notizen festzuhalten. Dabei kann jede:r auf die Erkenntnisse aus der Vorrunde zurückgreifen. Manche Teilnehmer:innen hatten nach dem vorigen Workshop-Modul vielleicht neue Ideen, auch diese können nun festgehalten werden.
Dauer: ca. 10 Minuten.

3. Schritt

Anschließend werden die einzelnen Paare gebeten, ihre wichtigsten Erkenntnisse oder Ideen **mit der gesamten Gruppe** zu teilen. Die neuen Ideen können z.B. auf einem “Ideenparkplatz”-Plakat festgehalten werden. Die Moderator:innen ergänzen Inhalte des letzten Moduls, die von den Kleingruppen womöglich nicht erwähnt werden.
Dauer: ca. 20 Minuten.

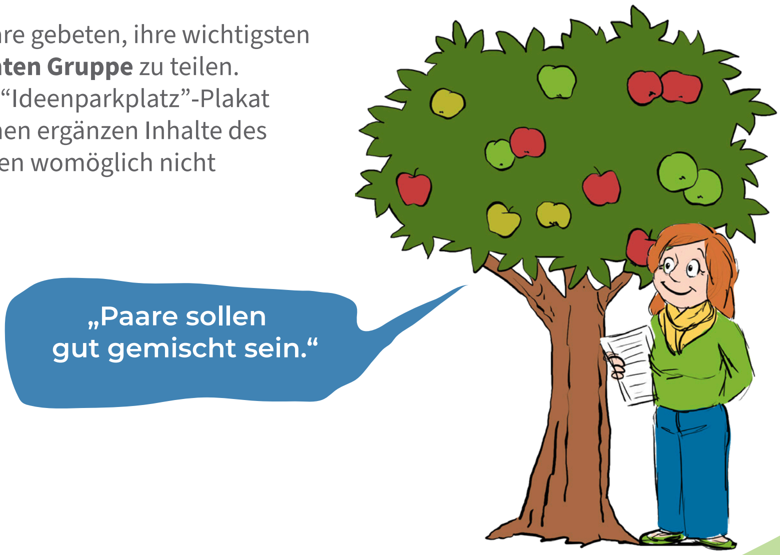

Methode 17: Erfinder:in
Erfinder:in

Warum? Neue Lösungsansätze erkunden.

Dauer? 2,5 Stunden

Material:

- Bausteine *(z.B. Lego Classic Set)*
- Pfeifenputzer, Knetmasse *(optional)*
- Erfindungsarbeitsblatt *(siehe Materialsammlung)*

Beschreibung

Die nächste Übung macht richtig Spaß: Die Teilnehmenden werden zu Erfinder:innen! Dabei wird ihrer **Kreativität** keine Grenzen gesetzt. Durch das Arbeiten mit den Händen werden kreative Prozesse im Kopf ausgelöst und ein freies Denken ermöglicht. Die verschiedenen Bausteine können wunderbar als Metaphern eingesetzt werden. Diese Methode gliedert sich in vier Bereiche:

1. Vorbereiten:
Zu Beginn werden die Teilnehmer:innen in **drei Gruppen** aufgeteilt: pro Forschungsfrage, die in Modul 2 entwickelt wurde, eine Gruppe. Die Gruppen erhalten Bausteine, Pfeifenputzer oder Knetmasse. Oder all das. Die Materialien liegen auf Tischen im Raum oder werden den Teilnehmenden direkt ausgehändigt. Wichtig: Der Kreativität sind **keine Grenzen** gesetzt, es gibt kein richtig oder falsch! Das betont das Co-Moderationsteam gleich zu Beginn.
Dauer: 20 Minuten.

2. Bauen:
Bezugnehmend auf die Forschungsfragen und damit einhergehenden Problemstellungen, die beim Inselhüpfen diskutiert wurden, werden nun Erfindungen, sogenannte „Problemlöser“ entwickelt. Mithilfe der Materialien bauen die Gruppen ihre Erfindung(en). Diese dürfen vollkommen frei erfunden und losgelöst von physikalischen Gesetzen sein.
Dauer: 90 Minuten.

3. Beschreiben:
Die Teilnehmer:innen nehmen nun ihr Erfindungs-Arbeitsblatt zur Hand. Darauf beschreiben sie ihre Erfindungen.

- Welches **Problem** löst deine Erfindung?
- Welche **Eigenschaften** hat deine Erfindung?
- Welchen **Namen** hat deine Erfindung?

Dauer: ca. 10 Minuten.

„Nicht vergessen, die Ergebnisse auf die Arbeitsblätter zu stellen und zu fotografieren! Bei dieser Übung darf ‚out of the box‘ gedacht werden.“

20 Minuten Pause

4. Teilen:
Nun stellen die Gruppen ihre Erfindungen vor. Jede:r Teilnehmer:in soll annähernd die gleiche Zeit bekommen.
Dauer: 30 Minuten.

Methode 18: Abschluss
Blitzlicht

Warum? Abschluss, Reflexion.

Dauer? 5-10 Minuten

Beschreibung

Geschafft! Nach der kreativen Arbeit lassen wir den Workshoptag ausklingen. Vorher sollte kurz die Stimmung der Gruppe eingefangen und die **Möglichkeit für Feedback** gegeben werden.
Das Co-Moderationsteam bittet jede:n um einen Satz, wie es der Person nach dem dritten Modul geht. Auf die Wortmeldungen soll nicht geantwortet werden. Auch das Co-Moderationsteam beteiligt sich.

Anschließend Verabschiedung und evtl. **organisatorische Hinweise** zu den Themen Abendessen und Unterkunft.

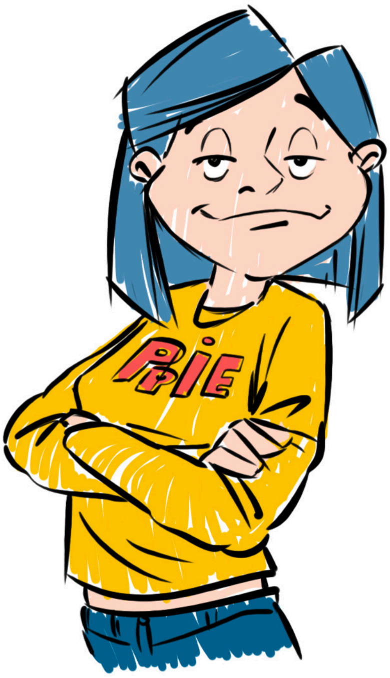

Studiendesign entwickeln

Vorbereiten für Modul 4:

- ☐ Präsentation & Arbeitsblätter *(21. Studiendesign-Wegweiser)*
- ☐ ABC-Plakat *(24. Reflexion)*
- ☐ Feedbackbögen oder Online-Survey mit QR-Code *(25. Feedback)*

Ziel des Moduls: Entwicklung des Studiendesigns

Die Forschungsfragen sind formuliert und verschiedene kreative Lösungsansätze wurden bereits etabliert. Jetzt lernt die Gruppe **das Studiendesign** kennen und wendet es auf die eigenen Ergebnisse an. Zum Schluss gibt das Co-Moderationsteam einen Ausblick auf die Zeit nach dem Workshop.

Dauer: Ca. 7,5 Stunden (z.B. von 9:00-16:30)

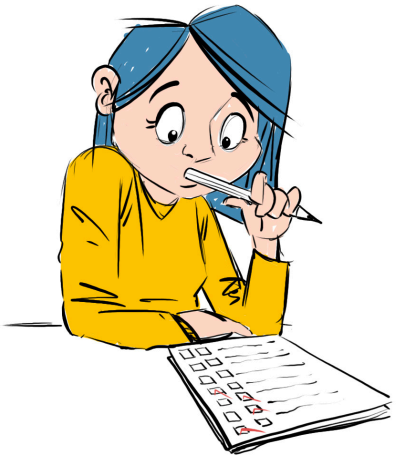

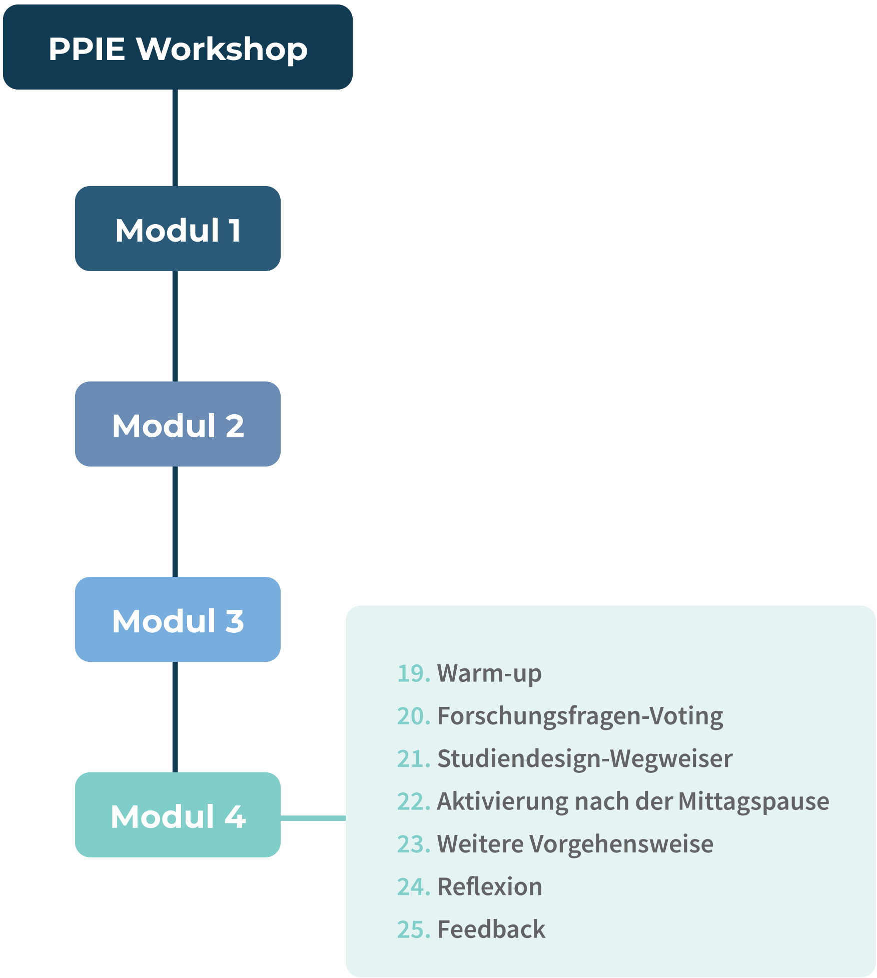

Methode 19: Warm-Up
Best Picture Contest

Warum? Aktivierung, Einstimmung auf das Thema.

Dauer? 30-40 Minuten

Material:

- Mobiltelefone der Teilnehmer:innen

Beschreibung

Der letzte Workshoptag startet kreativ! Die Teilnehmer:innen werden zu Fotograf:innen und haben die Aufgabe, den bisherigen Workshop **in einem Bild** auszudrücken. Es ist egal, ob sie ein Foto aufnehmen oder im Internet recherchieren. Wichtig ist jedoch, dass das Bild wiedergibt, welches Gefühl sie in Bezug auf die bisher erarbeiteten Ergebnisse haben (Forschungsfragen, Erfindungen).
Dauer: Für die Aufnahmen/Recherche reichen etwa **10 Minuten.**

Die Resultate werden anschließend in der Gruppe vorgestellt und gegebenenfalls diskutiert. Jede:r Teilnehmer:in beginnt mit dem Satz „Ich habe dieses Bild gewählt, weil…“.

Methode 20: Forschungsfragen-Voting
Abstimmung

Warum? Reduzieren auf eine Forschungsfrage, in Vorbereitung auf den nächsten Arbeitsschritt (Studiendesign).

Dauer? 10-15 Minuten

Material:

- Entweder Flipcharts & Klebepunkte
- oder Mentimeter-Voting

Beschreibung

Für die Entwicklung des Studiendesigns ist es vorteilhaft, sich auf eine Forschungsfrage zu konzentrieren. Das geschieht in diesem Schritt: Zunächst werden die drei Forschungsfragen, die bereits in Modul 2 erarbeitet wurden, erneut in Erinnerung gerufen, indem die Plakate nebeneinandergehängt werden. Nun beschließt die Gruppe, welche Forschungsfrage **am relevantesten für das Projekt** ist. Dies kann z.B. durch das Vergeben von Klebepunkten geschehen, oder durch eine Abstimmung mit dem digitalen Tool **Mentimeter.**

Sollte die Gruppe beschließen, mit mehreren Forschungsfragen weiterzuarbeiten, kann stattdessen auf dieselbe Weise eine **Priorisierung** durchgeführt werden.

20 Minuten Pause

Methode 21: Studienwegweiser
Placement-Methode

Warum? Erarbeitete Forschungsfragen in ein Studiendesign einbetten.

Dauer? 80-90 Minuten

Material:

- Lab-Book-Arbeitsblatt & Präsentation "Studiendesign-Wegweiser" *(siehe Lab-Book + Materialsammlung)*
- Moderationskärtchen (5 Farben)
- Flipcharts
- Stifte
- Magnete oder Klebeband, um Kärtchen aufzuhängen

Beschreibung

Die Forschungsfrage haben wir, kreative Lösungsansätze auch. Jetzt muss die Gruppe ein Studiendesign dafür entwickeln. Damit jede:r gut beitragen kann, besteht die Einheit aus 5 aufeinander aufbauenden Schritten: Einzelarbeit, Gruppenarbeit, Plenumsdiskussion, Meinungsbild, Dokumentation.

1. Einzelarbeit:
Jede:r Teilnehmer:in hat im Lab-Book ein Arbeitsblatt mit fünf Fragestellungen. Diese können zusätzlich auf die Wand projiziert oder auf ein Flipchart geschrieben werden (Themen: Planung, Methodik, Stichprobe, Erhebung, Offenes – ***siehe Lab-Book und Materialsammlung***). In einer Einzelarbeit versuchen die Teilnehmer:innen, jede Fragestellung nun **in Bezug auf die Forschungsfrage** bestmöglich zu beantworten, und auch Unklarheiten in ihr Lab-Book zu notieren.
Dauer: ca. 45 Minuten.

2. Gruppenarbeit:
Nun werden 3-4 Kleingruppen gebildet. Wichtig: In jeder Gruppe sollen unterschiedliche Expertisen vertreten sein! Hier werden nun die **5 Fragestellungen gemeinsam durchgesprochen**, dabei bringt jede:r die Ergebnisse der jeweiligen Einzelarbeit ein. Das Ziel ist, für jede Fragestellung eine oder mehrere gemeinsame Antworten, Ideen, Bedenken oder Vorschläge zu finden, mit denen alle einverstanden sind. Diese werden auf Moderationskärtchen geschrieben. Wenn gewünscht, kann das Co-Moderationsteam pro Fragestellung eine bestimmte Kärtchenfarbe vorgeben.
Dauer: ca. 60 Minuten.

1,5 Stunden Mittagpause

Methode 22: Aktivierung nach der Mittagspause
Gewitteralarm

Warum? Aktivierung nach der Mittagspause.

Dauer? 5-10 Minuten

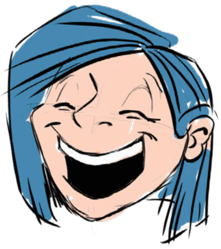

Beschreibung

Wir kennen das bereits aus dem vorigen Modul: Nach dem Mittagessen ist das Energielevel sehr niedrig. Zeit für eine kurze Aktivierungsübung. So geht's: Die Teilnehmer:innen bilden einen Kreis. In der Mitte steht ein:e Moderator:in und leitet wie folgt an:

- **"Sonnenschein"**: freundliches Lächeln
- **„Die Ruhe vor dem Sturm"**: konzentrierte Ruhe und Stille
- **„Die Blätter rauschen im Wind"**: Fingerspitzen aneinander reiben
- **„Der Regen naht"**: Hände aneinander reiben
- **„Die ersten Tropfen fallen"**: mit den Fingern einer Hand langsam schnippen
- **„Der Regen wird stärker"**: mit den Fingern beider Hände fest und schnell schnippen
- **„Der Regen prasselt"**: schnell klatschen
- **„Donner setzt ein"**: zusätzlich stampfen, 1-2 Teilnehmer:innen legen sich auf den Boden
- **„Höhepunkt des Unwetters"**: alle klatschen und stampfen

Danach das Unwetter in umgekehrter Reihenfolge zurückbauen, bis die Sonne wieder scheint!

Methode 21: Studiendesign-Wegweiser (Fortsetzung)

Konsensfindungsprozess

Warum? Erarbeitete Forschungsfragen in ein Studiendesign einbetten.

Dauer? 80-90 Minuten

Material:

- Lab-Book-Arbeitsblatt & Präsentation "Studiendesign-Wegweiser" *(siehe Lab-Book + Materialsammlung)*
- Moderationskärtchen (5 Farben)
- Flipcharts
- Stifte
- Magnete oder Klebeband, um Kärtchen aufzuhängen

Beschreibung

3. Plenumsdiskussion:

Die Gruppen haben sich ausführlich mit dem Studiendesign auseinandergesetzt, während der Mittagspause wurde wahrscheinlich weiterdiskutiert. Jetzt ist es Zeit, die Ergebnisse ins Plenum zu tragen und dort zu diskutieren. **Jede Gruppe präsentiert ihre Ergebnisse** und klebt ihre Kärtchen zur jeweiligen Fragestellung (die zuvor vom Co-Moderationsteam auf Flipcharts oder Pinnwände geschrieben wurden). In einer offenen Diskussion werden die Ergebnisse geclustert und gegebenenfalls priorisiert. So entstehen in gemeinsamer Arbeit fünf Plakate mit den wichtigsten Ideen, Vorschlägen und auch Fragen zum Studiendesign. **Wichtig:** Das Co-Moderationsteam stellt sicher, dass alle Stimmen gehört werden und der Austausch respektvoll ist (Verweis auf die "Spielregeln").
Dauer: ca. 60 Minuten.

4. Meinungsbild:

Nach der Diskussion bittet das Co-Moderationsteam die Teilnehmer:innen darum, für jedes der fünf Plakate ihre Zustimmung oder Ablehnung auszudrücken. Damit kein Gruppenzwang entsteht, empfiehlt es sich, dafür ein digitales Abstimmungstool wie z.B. **Mentimeter** einzusetzen. Alternativ kann durch "**blindes Armheben**" abgestimmt werden: Teilnehmer:innen halten dabei ihre Augen geschlossen. Das Ziel ist es, einen Konsens zu erreichen, mit dem alle Gruppenmitglieder zufrieden sind.
Dauer: 10-20 Minuten.

5. Dokumentation:

Die getroffenen Entscheidungen für das Studiendesign sollten festgehalten werden, um sicherzustellen, dass alle Teilnehmer:innen das gleiche Verständnis haben und die Beschlüsse später nachvollziehen können. Zusätzlich können Teilnehmer:innen die Ergebnisse in ihr Lab-Book eintragen. Dafür muss dann etwas extra Zeit eingeplant werden.

20 Minuten Pause

Methode 23: Weitere Vorgehensweise
Impuls-Vortrag

Warum? Die Teilnehmer:innen werden über die nächsten Schritte informiert.

Dauer? 30 Minuten

Beschreibung

Wie geht es nun weiter? Das hängt vom jeweiligen Projekt ab! Daher muss diese Sequenz von jedem Forschungsprojekt individuell gestaltet werden. In einem kurzen Impuls-Vortrag informiert das Co-Moderationsteam die Teilnehmer:innen darüber, wie es nun weitergehen kann. Anschließend wird anhand der Fragen der Teilnehmer:innen gemeinsam besprochen, was mit den Ergebnissen geschieht. Dabei sollten jedenfalls folgende Fragen beantwortet werden:

- Was geschieht mit den **Ergebnissen** des Workshops?
- Wie wird es nun **weitergehen** mit dem Projekt?
- Bleibt die Gruppe weiterhin **involviert**? Wenn ja: Wie?

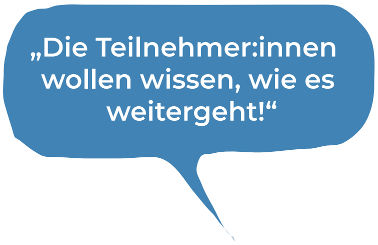

Optional: Falls Teilnehmer:innen in den weiteren Projektverlauf eingebunden werden, sollten sie aktiv überlegen, wie. In einer kurzen Sequenz **(ca. 10 Minuten)** schreiben sie in ihr Lab-Book, welche Vorstellungen sie dazu haben. Danach wird in der Gruppe darüber diskutiert und die nächsten Schritte werden fixiert **(ca. 20 Minuten)**. Es kann auch eine Person stellvertretend für die Gruppe weiter involviert bleiben.

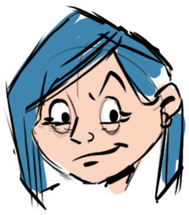

Methode 24: Reflexion
ABC-Methode

Warum? Reflektieren und Verfestigen der Workshop-Inhalte.

Dauer? 15-20 Minuten

Material:

- Vorgefertigtes Material
- Stifte

Beschreibung

Wir nähern uns dem Ende und lassen den Workshop noch einmal Revue passieren. Auf einem vorgefertigten Plakat hat das Co-Moderationsteam (oder ein Helferlein aus der Gruppe) in drei Spalten alle **Buchstaben des Alphabets** aufgeschrieben, mit genügend Abstand dazwischen. Nun schreiben die Teilnehmer:innen aufgefordert zu jedem Buchstaben einen Begriff, der sich auf den Workshop bezieht. Das können sowohl Fachbegriffe als auch atmosphärische Begriffe sein. Ziel ist es, zu jedem Buchstaben mindestens ein Wort zu finden. Es ist ausdrücklich erwünscht, dass bei den einzelnen Buchstaben auch mehrere Begriffe stehen.

Tipp: Im Querformat ist auf dem Plakat mehr Platz!

Methode 25: Feedback
Wetterbericht & Feedbackbögen

Warum? Atmosphärisches und anonymes Feedback.

Dauer? 15 Minuten

Material:

- Vorbereitete Online-Umfrage oder ausgedruckte Feedbackbögen
- Stoffball

Beschreibung

Der Workshop war ein voller Erfolg, alle gehen glücklich nach Hause. Oder? Das sollten die Teilnehmer:innen selbst sagen. Deshalb gibt es zum Schluss eine kurze Feedback-Runde: den **Wetterbericht**. Das Co-Moderationsteam leitet die Aufgabe wie folgt ein: „Stell dir vor, du gibst Feedback zu diesem heutigen Tag. Mache dies in Form eines kurzen Wetterberichts!“ Wer dran war, nominiert die:den nächste:n Feedbackgeber:in (z.B. durch Ball-Zuwurf). Erfahrungsgemäß werden einige Teilnehmer:innen ihrem “Wetterbericht” noch ein paar persönliche Sätze hinzufügen, was ausdrücklich okay ist. Auch das Co-Moderationsteam beteiligt sich.

Anschließend sollten **5 Minuten** für anonymes, schriftliches Feedback eingeplant werden.

Dann: Abschied, Abschluss. Geschafft!

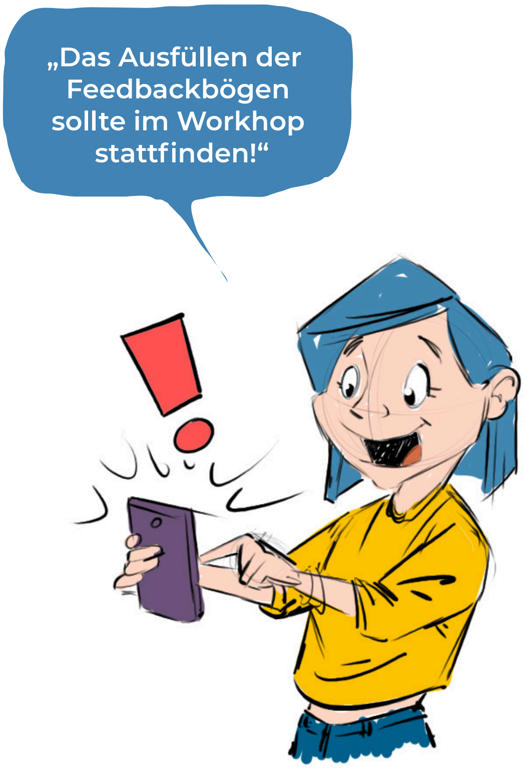

GEDANKENPARKPLATZ

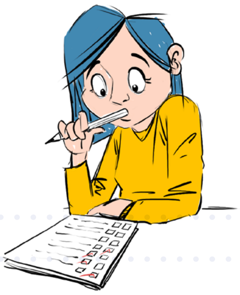

Nach dem Workshop

„Auch ich freue mich über Feedback zum Workshop und zu dieser Anleitung! KONTAKT: office@ccieurope.eu“

Was es nach dem Workshop braucht

Evaluierung durchführen

Nach dem Workshop ist vor dem nächsten Workshop. Um Stärken zu stärken und aus Schwächen zu lernen, empfiehlt sich eine genaue **Analyse des Feedbacks** der Teilnehmer:innen. Wichtig: Kritikpunkte ernst nehmen und objektiv evaluieren, ob diese im Falle weiterer Workshops eine Anpassung erforderlich machen.

Dokumentation abwickeln

Während des Workshops sollten die Ergebnisse **fotografisch dokumentiert** werden. Wird ein:e Teilnehmer:in damit beauftragt, sollte nicht vergessen werden, diese am Ende des Workshops **an das Co-Moderationsteam** zu übermitteln. Die Fotos können systematisch benannt und abgelegt werden, damit sie auch später noch eindeutig zuordenbar sind, wenn die Forschungsgruppe damit arbeitet.

Falls die Dokumentation – oder Teile davon – den Teilnehmer:innen zur Verfügung gestellt wird, können die entsprechenden Fotos z.B. in einer PDF-Datei zusammengestellt und übermittelt werden.

Wie geht es weiter?

Egal, wie es weiter geht: **Die Workshop-Teilnehmer:innen wollen es wissen**. Aus gutem Grund: Sie haben zwei Wochenenden investiert, um das Projekt zu unterstützen. Wie die Workshop-Ergebnisse konkret im weiteren Forschungsprozess umgesetzt werden, kann dabei von Projekt zu Projekt unterschiedlich sein. Wir empfehlen, den Workshop-Teilnehmer:innen einen Ausblick zu geben und folgende Fragen zu beantworten:

- ? Wie geht es mit der **Forschungsgruppe** weiter? Bleibt sie bestehen, wird es weitere Projekte für sie geben? War das eine einmalige Forschungsreise?
- ? Wie wird die Gruppe weiter miteinbezogen, wenn das **Projekt weitergeht**? Wo müssen sie involviert werden?
- ? Gibt es weiterführende Projekte, wo die Gruppe selbst **mitwirken** kann?
- ? Was passiert mit dem **Ergebnis** des Workshops?
- ? Wie wird die Gruppe über die Ergebnisse der Forschung **informiert**?

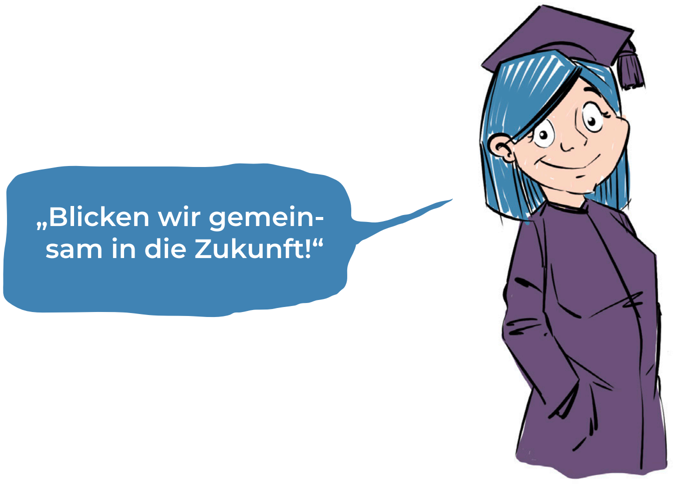

GEDANKENPARKPLATZ

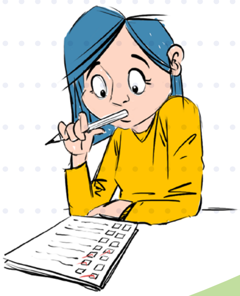

Zum Schluss

Hallo, hier bin ich nochmal.
Hat der Workshop Spaß gemacht?
Ich hoffe es!

Ich gratuliere der gesamten Workshopgruppe. Mit der Durchführung wurde ein Stück **Pionierarbeit** geleistet: Hin zu wahrhaft inklusiver Forschung und Entwicklung. Oder, um beim Bild des Apfels aus der Einleitung zu bleiben: Die Forschungsgruppe kennt jetzt nicht nur den Umfang des Apfels, das Gewicht und andere messbare Daten – sie berücksichtigt nun auch den Geschmack als wesentliches Merkmal. Im Klartext: Die Forschungsgruppe bindet die **Expertise von Patient:innen** von Anfang an in das Projekt ein.

Und das hat viele Vorteile:

- Die Forschungsfragen sind **genauer**.
- Die Forschungsergebnisse sind **hochwertig und relevant**.
- Die Bedürfnisse von Patient:innen stehen ernsthaft **im Mittelpunkt**.
- Forschung und Versorgung werden nachvollziehbarer und **gewinnen an Bedeutung**.

Apfel hin oder her: Durch den Workshop haben alle zum gemeinsamen Ziel beigetragen: **Die Forschung und die Versorgung zu verbessern**. Damit der Apfel weniger sauer ist.

Bis zum nächsten Mal!
Claire, Patient Expert

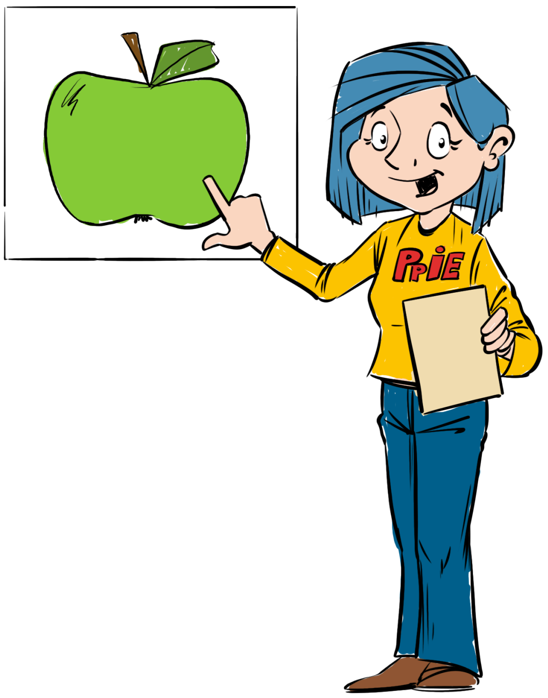

GEDANKENPARKPLATZ

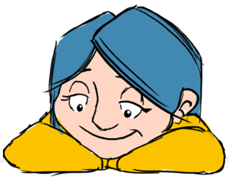

Literatur zum weiterlesen

Zum Weiterlesen

Belbin, R. M. (2010). ***Team Roles at Work.*** Butterworth-Heinemann. https://books.google.at/books?id=hF2yJ-zYfUBAC

Bentley, C., Costa, S., Burgess, M. M., Regier, D., McTaggart-Cowan, H. & Peacock, S. J. (2018). ***Trade-offs, fairness, and funding for cancer drugs: Key findings from a deliberative public engagement event in British Columbia, Canada.*** BMC Health Services Research, 18(1), 1–13. https://doi.org/10.1186/s12913-018-3117-7

Bootsma, T. I., Duijveman, P., Pijpe, A., Scheelings, P. C., Witkamp, A. J. & Bleiker, E. M. A. (2020). ***Unmet information needs of men with breast cancer and health professionals.*** Psycho-Oncology, 29(5), 851–860. https://doi.org/10.1002/pon.5356

Boyer, E. J., Van Slyke, D. M. & Rogers, J. D. (2016). ***An Empirical Examination of Public Involvement in Public-Private Partnerships: Qualifying the Benefits of Public Involvement in PPPs.*** Journal of Public Administration Research and Theory, 26(1), 45–61. https://doi.org/10.1093/jopart/muv008

Burges Watson, D. L., Lewis, S., Bryant, V., Patterson, J., Kelly, C., Edwards-Stuart, R., Murtagh, M. J. & Deary, V. (2018). ***Altered eating: A definition and framework for assessment and intervention.*** BMC Nutrition, 4(1). https://doi.org/10.1186/s40795-018-0221-3

Casanova, T., Black, C., Rafiq, S., Hugill-Jones, J., Read, J. C. A. & Vancleef, K. (2020). ***The impact of active research involvement of young children in the design of a new stereotest.*** Research Involvement and Engagement, 6(1), 1–12. https://doi.org/10.1186/s40900-020-00194-6

COPE Group. (2022). ***Good to know! That's PPIE*** (english). https://www.youtube.com/watch?v=HHITKZ5tZcY&ab_channel=COPEGroup

Costa, S., Bentley, C., Regier, D. A., McTaggart-Cowan, H., Mitton, C., Burgess, M. M. & Peacock, S. J. (2019). ***Public perspectives on disinvestments in drug funding: Results from a Canadian deliberative public engagement event on cancer drugs.*** BMC Public Health, 19(1). https://doi.org/10.1186/s12889-019-7303-2

De Bono, E. (1985). ***Six Thinking Hats.*** Little, Brown. https://books.google.at/books?id=0lNmQgAACAAJ

De Shazer, S. (1988). ***Clues: Investigating solutions in brief therapy.*** W. W. Norton & Co.

Dennison, R. A., Boscott, R. A., Thomas, R., Griffin, S. J., Harrison, H., John, S. D., Moorthie, S. A., Morris, S., Rossi, S. H., Stewart, G. D., Thomas, C. V. & Usher-Smith, J. A. (2022). ***A community jury study exploring the public acceptability of using risk stratification to determine eligibility for cancer screening.*** Under Review, April, 1–18. https://doi.org/10.1111/hex.13522

Gamble, C., Dudley, L., Allam, A., Bell, P., Goodare, H., Hanley, B., Preston, J., Walker, A., Williamson, P. & Young, B. (2014). ***Patient and public involvement in the early stages of clinical trial development: A systematic cohort investigation.*** BMJ Open, 4(7), 1–11. https://doi.org/10.1136/bmjopen-2014-005234

Garland, S. N., Trevino, K., Liou, K. T., Gehrman, P., Spiguel, E., MacLeod, J., Walker, D. A. H., Glosik, B., Seluzicki, C., Barg, F. K., Barg, F. K. & Mao, J. J. (2021). ***Multi-stakeholder perspectives on managing insomnia in cancer survivors: recommendations to reduce barriers and translate patient-centered research into practice.*** Journal of Cancer Survivorship, 15(6), 951–960. https://doi.org/10.1007/s11764-021-01001-1

Gelkopf, M. J., Avramov, I., Baddeliyanage, R., Ristevski, I., Johnson, S. A., Flegg, K. & Dimaras, H. (2020). ***The Canadian retinoblastoma research advisory board: A framework for patient engagement.*** Research Involvement and Engagement, 6(1). https://doi.org/10.1186/s40900-020-0177-8

Gilljam, B. M., Nygren, J. M., Svedberg, P. & Arvidsson, S. (2020). ***Impact of an electronic health service on child participation in pediatric oncology care: Quasiexperimental study.*** Journal of Medical Internet Research, 22(7). https://doi.org/10.2196/17673

Gray, R., Brasier, C., Zirnsak, T. M. & Ng, A. H. (2021). ***Reporting of patient and public involvement and engagement (PPIE) in clinical trials published in nursing science journals: a descriptive study.*** Research Involvement and Engagement, 7(1), 1–7. https://doi.org/10.1186/s40900-021-00331-9

Hayes, H., Buckland, S. & Tarpey, M. (2021). ***Briefing notes for researchers: Public Involvement in NHS, public health and social care research. National Institute for Health Research***. http://www.invo.org.uk/wp-content/uploads/2014/11/9938_INVOLVE_Briefing_Notes_WEB.pdf

Høeg, B. L., Tjørnhøj-Thomsen, T., Skaarup, J. A., Langstrup, H., Zoffmann, V., Saltbaek, L., Karlsen, R. V., Dalton, S. O., Johansen, C. & Bidstrup, P. E. (2019). ***Whose perspective is it anyway? Dilemmas of patient involvement in the development of a randomized clinical trial–a qualitative study.*** Acta Oncologica, 58(5), 634–641. https://doi.org/10.1080/0284186X.2019.1566776

Leahy, A. B., Schwartz, L. A., Li, Y., Reeve, B. B., Bekelman, J. E., Aplenc, R. & Basch, E. M. (2021). ***Electronic symptom monitoring in pediatric patients hospitalized for chemotherapy.*** Cancer, 127(16), 2980–2989. https://doi.org/10.1002/cncr.33617

Literatur zum weiterlesen

Leiss, U., Lamplmaier, D., Fries, J., Schreiber-Gollwitzer, B. M., Grießmeier, B., Hesselbarth, B., Lein-Köhler, I., Nest, A., Schröder, Weiler-Wichtl, L. J. & Schröder, H. M. (2019). ***Bestandsanalyse zur Leitlinie „Psychosoziale Versorgung in der Pädiatrischen Onkologie und Hämatologie“.***

McAlpine, K., Breau, R. H., Stacey, D., Knee, C., Jewett, M. A. S., Cagiannos, I., Morash, C. & Lavallée, L. T. (2019a). ***Development and acceptability testing of a patient decision aid for individuals with localized renal masses considering surgical removal with partial or radical nephrectomy.*** Urologic Oncology: Seminars and Original Investigations, 37(11), 811.e1-811.e7. https://doi.org/10.1016/j.urolonc.2019.08.014

McAlpine, K., Breau, R. H., Stacey, D., Knee, C., Jewett, M. A. S., Cagiannos, I., Morash, C. & Lavallée, L. T. (2019b). ***Development and acceptability testing of a patient decision aid for individuals with localized renal masses considering surgical removal with partial or radical nephrectomy.*** Urologic Oncology: Seminars and Original Investigations, 37(11), 811.e1-811.e7. https://doi.org/10.1016/j.urolonc.2019.08.014

Mentimeter AB (publ). (2023). ***Mentimeter.***

Polanco, A., Al-Saadi, R., Tugnait, S., Scobie, N. & Pritchard-Jones, K. (2021). ***Setting international standards for patient and parent involvement and engagement in childhood, adolescent and young adult cancer research: A report from a European Collaborative Workshop.*** Cancer Reports. https://doi.org/10.1002/cnr2.1523

Price, A., Albarqouni, L., Kirkpatrick, J., Clarke, M., Liew, S. M., Roberts, N. & Burls, A. (2018). ***Patient and public involvement in the design of clinical trials: An overview of systematic reviews.*** Journal of Evaluation in Clinical Practice, 24(1), 240–253. https://doi.org/10.1111/jep.12805

Riter, R. N. & Weiss, R. S. (2019). ***Connecting students with patients and survivors to enhance cancer research training.*** Cancer Research, 79(16), 4011–4014. https://doi.org/10.1158/0008-5472.CAN-19-0603

Rubio, M. A., Guevara-Aladino, P., Urbano, M., Cabas, S., Mejia-Arbelaez, C., Rodriguez Espinosa, P., Rosas, L. G., King, A. C., Chazdon, S. & Sarmiento, O. L. (2022). ***Innovative participatory evaluation methodologies to assess and sustain multilevel impacts of two community-based physical activity programs for women in Colombia.*** BMC Public Health, 22(1). https://doi.org/10.1186/s12889-022-13180-2

Schurman, J. V., Gayes, L. A., Slosky, L., Hunter, M. E. & Pino, F. A. (2015). ***Publishing quality improvement work in clinical practice in pediatric psychology: The “why” and “how To.”*** Clinical Practice in Pediatric Psychology, 3(1), 80–91. https://doi.org/10.1037/cpp0000084

Signorelli, C., Wakefield, C. E., McLoone, J. K., Mateos, M. K., Aaronson, N. K., Lavoipierre, A., Cohn, R. J., Alvaro, F., Corbett, R., Downie, P., Egan, K., Ellis, S., Emery, J., Fardell, J., Foreman, T., Gabriel, M., Girgis, A., Graham, K., Johnston, K., […] Yallop, K. (2021). ***A cost-effective approach to increasing participation in patient-reported outcomes research in cancer: A randomized trial of video invitations.*** International Journal of Cancer, 148(4), 971–980. https://doi.org/10.1002/ijc.33244

Taylor, R. M., Fern, L. A., Barber, J., Alvarez-Galvez, J., Feltbower, R., Morris, S., Hooker, L., McCabe, M. G., Gibson, F., Raine, R., Stark, D. P. & Whelan, J. S. (2019). ***Description of the BRIGHTLIGHT cohort: The evaluation of teenage and young adult cancer services in England.*** BMJ Open, 9(4). https://doi.org/10.1136/bmjopen-2018-027797

Taylor, R. M., Whelan, J. S., Gibson, F., Morgan, S. & Fern, L. A. (2018). ***Involving young people in BRIGHTLIGHT from study inception to secondary data analysis: Insights from 10 years of user involvement.*** Research Involvement and Engagement, 4(1). https://doi.org/10.1186/s40900-018-0135-x

Weiler-Wichtl, L. J., Leiss, U., Gojo, J., Kienesberger, A., Hansl, R., Hopfgartner, M. & Schneider, C. (2023). ***Good to know – This is PPIE! Development of a training tool for Public and Patient Involvement and Engagement in Pediatric Oncological Research.*** Cancer Reports, in review.

Zum Schluss

Impressum

Ein Projekt der Medizinischen Universität Wien mit der COPE Group in Kooperation mit CCI Europe unterstützt von Kurvenkratzer, NF Kinder, EUPATI Austria und Survivors Österreich, finanziert durch das LBG Open Innovation in Science Center.

Medizinische Universität Wien, Universitätsklinik für Kinder- und Jugendheilkunde, Comprehensive Center for Pediatrics, Währinger Gürtel 18-20, 1090 Wien, www.meduniwien.ac.at.
CCI – Childhood Care International Europe, Handelskai 94-96, 1200 Wien, www.ccieurope.eu.

Illustrationen von Bone Buddrus, grafische Gestaltung und Design von Kurvenkratzer.

Projektteam: Gelebtes PPIE bedeutet ein interdisziplinäres und ganzheitliches Denken. Um eine Vielzahl an Perspektiven einzubringen, wurde das Projektteam bewusst aus Personen mit vielfältigen Expertisen zusammengestellt, die jeweils in verschiedenen Projektphasen ihre Beiträge leisteten.

Projektleitung:

Liesa J. Weiler-Wichtl, *Klinische und Gesundheitspsychologin, Medizinische Universität Wien/COPE Group*
Carina Schneider, *Psychologin, Patient Expert, CCI Europe*

Konzeption und Umsetzung:

Ulrike Leiss, *Klinische und Gesundheitspsychologin, Medizinische Universität Wien/COPE Group*
Hannah Gsell, *Psychologin, Patient Expert, CCI Europe, Survivors Österreich*
Anna Maletzky, *Klinische Psychologin, Medizinische Universität Wien*
Johannes Gojo, *Arzt, Medizinische Universität Wien*
Anita Kienesberger, *Patient Advocate, CCI Europe*
Claas Röhl, *Patient Advocate, NF Kinder, EUPATI*
Martina Hagspiel, *Patient Advocate, Kurvenkratzer*
Albina Hyseni-Bocolli, *Projektkoordinatorin, CCI Europe*
Lena Horvath, *Projektkoordinatorin, Kurvenkratzer*
Florian Lems, *Workshopkonzeptentwickler, Kurvenkratzer*
Tanya Kaindlbauer, *Lektorin, Kurvenkratzer*
Lena Kalinka, *Grafikerin, Kurvenkratzer*
Bone Buddrus, *Illustrator*

Involvierte Individual Patients & Carers und weitere Projektmitarbeiter:innen (Public Involvement):

Bettina Baldt, *Psychologin*
Lisa Cate, *Patient Expert*
Monika Czajka, *Patient Expert*
Sabine Karner, *Patient Expert*
Anna Lämmerer, *Patient Expert*
Rita Hansl, *Wissenschaftliche Assistentin*
Maximilian Hopfgartner, *Wissenschaftlicher Assistent*
Niklas Schober, *Individual Patient*
Delphine Heenen, *Patient Advocate*
Alice Gerbaux, *Patient Advocate, inhaltlicher Berater*

Marie Therese Gubi, *Patient Expert*
Maria Willomitzer, *Patient Expert*
Valentin Willomitzer, *Patient Expert*
Anna Zettl, *Patient Expert*
Sybille Zettl, *Patient Advocate*
Stefan Pinczolits, *Patient Expert*
Doris Schober, *Patient Expert*
Florian Müller, *Patient Expert*

Daniela Koczera, *Individual Patient*

Und zahlreiche weitere Individual Patients & Carers.

Gefördert durch:

LUDWIG BOLTZMANN GESELLSCHAFT
Open Innovation in Science Center

Das Projekt „Nothing about us without us“ sowie die „Junior research Academy“ wurden 2021 und 2022 im Rahmen des Calls für Public and Patient Involvement gefördert durch: LBG Open Innovation in Science Center Program Manager Public and Patient Involvement in Research

Nußdorfer Straße 64/2
1090 Vienna, Austria
www.ois.lbg.ac.at

Tel +43 (0) 1 513 27 50-68
M +43 (0) 660 215 57 41
thomas.palfinger@lbg.ac.at

Umgesetzt von:

MEDIZINISCHE UNIVERSITÄT WIEN
COPE GROUP
Childhood Cancer International EUROPE
KURVENKRATZER

In Kooperation mit:

EUPATI
NF Kinder
survivors AUSTRIA
