# Supplementary material for: Asking those who know their needs best: A framework for active engagement and involvement of childhood cancer survivors and parents in the process of psychosocial research—A workshop report
Source: Cancer Rep (Hoboken). 2024 May 20;7(5):e2071. doi: 10.1002/cnr2.2071 (PMC11104286; doi:10.1002/cnr2.2071)
Supplement: Supplementary file 4 — Supplementary Material 2: PPIE Lab Book. [file CNR2-7-e2071-s002.pdf]

ANHANG 1

LAB-BOOK

BRIDGING THE GAP

GEMEINSAM FORSCHEN IN DER JUNIOR RESEARCH ACADEMY

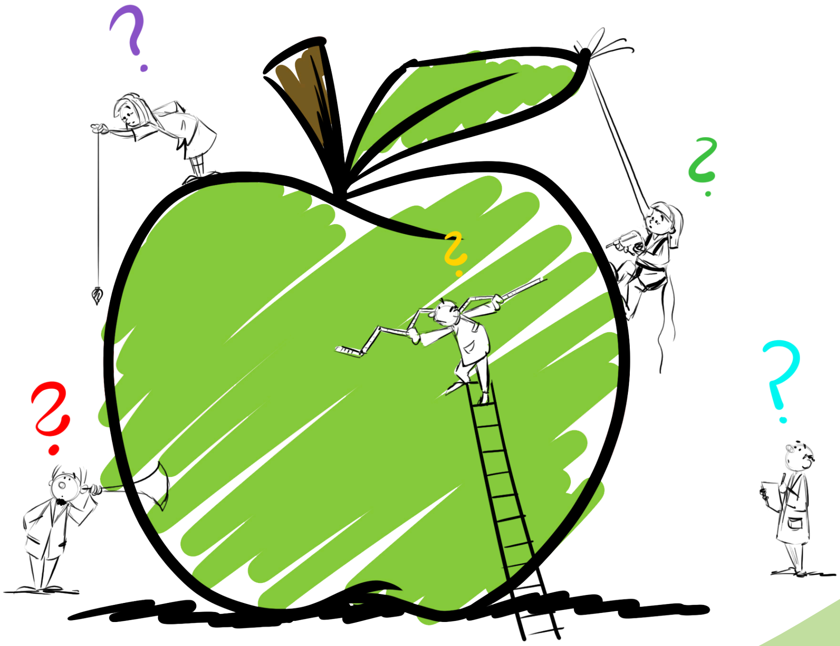

Finanziert durch:

LUDWIG BOLTZMANN GESELLSCHAFT
Open Innovation in Science Center

Umgesetzt von:

MEDIZINISCHE UNIVERSITÄT WIEN
COPE GROUP
Childhood Cancer International EUROPE
KURVENKRATZER

Vorwort

Hallo!

Mein Name ist Claire und ich habe ein Anliegen.
Ich möchte, dass besser verstanden wird, wie es ist, in einen sauren Apfel zu beißen. Jetzt wundern sich wahrscheinlich viele Leser:innen:
Worauf will sie hinaus?

Nun, der Apfel ist meine Metapher für Krebs. Ich habe ihn überlebt.
Und mein Pfad zur Genesung hat mich zu einer echten Expertin gemacht:
Ich weiß alles über meinen besonders sauren Apfel.
Vor allem, wie er schmeckt.

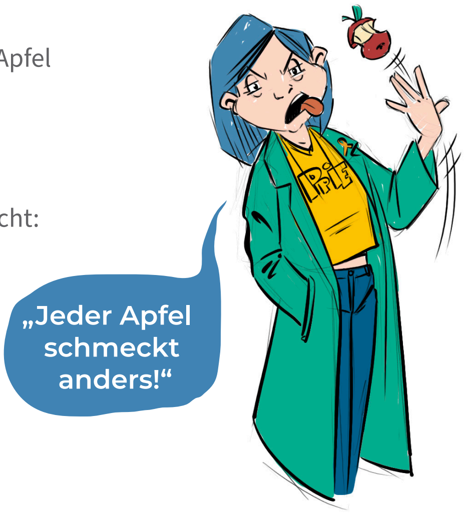

Einigen von euch kommt das wahrscheinlich bekannt vor:
Wenn ihr Patient:innen oder ihre Zugehörigen seid und an diesem Workshop teilnehmt, habt ihr vermutlich in euren eigenen sauren Apfel gebissen.

Das führt mich zur **Vision**, die mein Forschungsteam und ich haben:
Die Öffentlichkeit und Patient:innen in die Forschung einzubinden. Und diese dadurch zu verbessern, dass Forschungsfragen zielgenau gestellt werden. Damit jene Menschen im Mittelpunkt stehen, um die es geht: Die Öffentlichkeit und Patient:innen wie ich. Was das mit dem Apfel zu tun hat? Nun, es gibt ja bereits umfangreiche Methoden, einen Apfel zu vermessen, zu analysieren und zu behandeln. Und es gibt Expert:innen, die mit sehr viel **Know-how und Enthusiasmus** daran arbeiten. Manche ahnen es schon: Gemeint sind Forschende – etwa aus der Medizin, der Psychologie, der Pflege, der sozialen Arbeit und vielen weiteren Feldern.

Jetzt kommen wir zum Kern der Sache (und zum Zweck dieses Dokuments):
Um **wirksame Diagnostikmethoden und Therapien** zu entwickeln, braucht es neben dem Vermessen, Analysieren und Behandeln eben auch den Erfahrungswert, den Patient:innen mitbringen. Denn nur sie können wirklich beschreiben, wie der Apfel schmeckt. Es geht also um die Einbindung von Patient:innen in die Forschung, von Anfang an. Darum dreht sich auch der **Workshop**, zu dem du eingeladen wurdest. Er dient als Werkzeug, das es Patient:innen und Forschungsgruppen ermöglicht, gemeinsam **Forschungsfragen mit hoher Relevanz zu entwickeln**. Wissenschaftliche Fragen, die uns Patient:innen und unsere Zugehörigen von Anfang an einbinden und unsere Expertise und Bedürfnisse ernst nehmen. Und davon ausgehend **Projektpläne**, um die Forschung gemeinsam umzusetzen.

Das gilt für alle Bereiche, in denen Forschung passiert! Wie bei einem Puzzle müssen wir alle Expertisen, die den Körper, die Psyche und das Soziale betreffen, gleichermaßen berücksichtigen, um am Ende ein Gesamtbild zu erhalten und allen Bedürfnissen gerecht zu werden. Eine ganzheitliche, interdisziplinäre Grundhaltung nach dem biopsychosozialen Modell ist daher nötig – auch bei der Auswahl der Expert:innen.

In diesem Workshop behandeln wir das konkrete Thema:

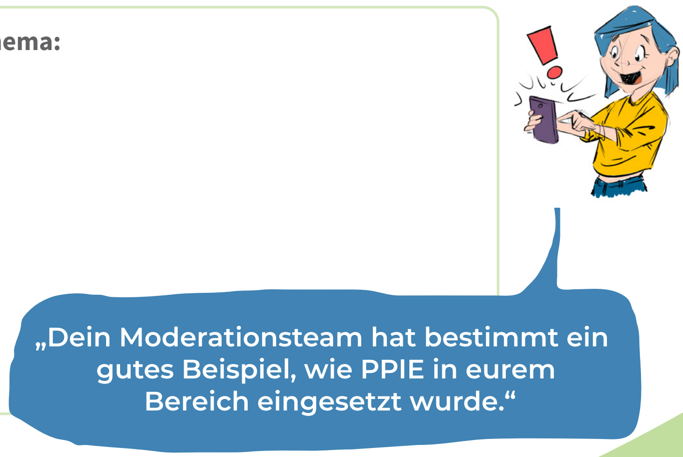

Vorwort

Dieses Dokument erhältst du als Vorbereitung für den Workshop.
Es enthält folgende Infos:

- Was ist “**Patient & Public Involvement and Engagement**”, kurz PPIE?
- Die “**Spielregeln**” für den Workshop
- Erweiterbares **PPIE-Wörterbuch** für den Workshop
- **Gedankenparkplatz** für Notizen und Gedanken

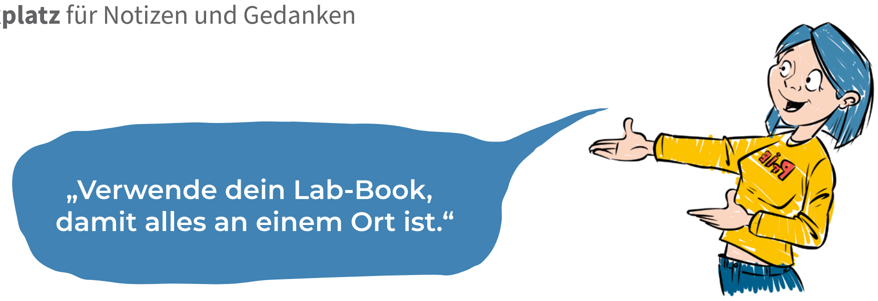

Eine Grundvoraussetzung für eine gute Zusammenarbeit ist, dass alle Teilnehmer:innen trotz ihres diversen Hintergrunds **gleichberechtigt** mitmachen können. Nur so kann das Ziel erreicht werden: Bridging the Gap! Also **eine Brücke schlagen** zwischen Patient:innen und Forschenden. Denn wir arbeiten alle auf dasselbe hin: Wir wollen den Apfel in seinen Facetten verstehen. Eben auch, wie er schmeckt. Wir alle wollen die **Forschung und Versorgung verbessern**.

Viel Spaß beim Workshop!

Claire,
Patient Expert

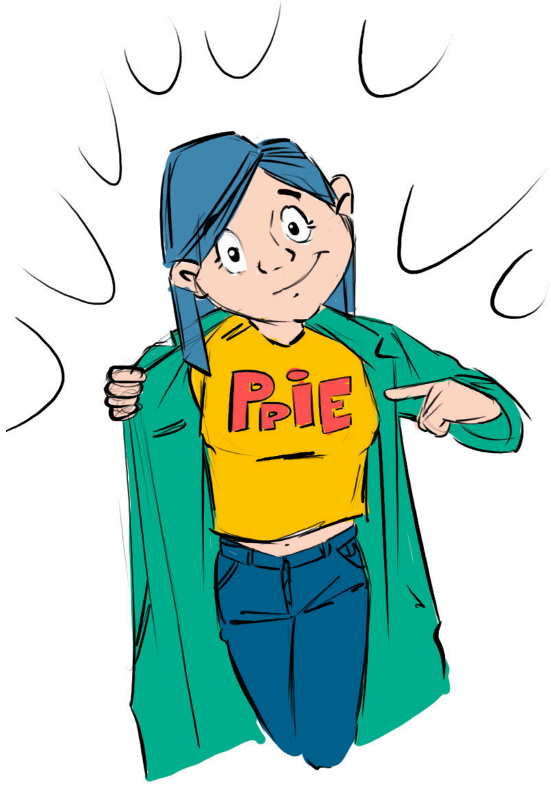

Intro

Warum Patient and Public Involvement & Engagement (PPIE)?

Forschung ohne Patient:innen ist vergleichbar mit einem Puzzle, bei dem ein Teilchen fehlt: Man kann zwar viele Einzelteile betrachten und analysieren, aber ohne das fehlende Stück bleibt das Gesamtbild unvollständig - es fehlt die Gewissheit, ob alle Zusammenhänge verstanden wurden. Es ist daher eine Verbesserung und ermutigend, dass die **Einbindung von Patient:innen** in der klinischen Forschung zunehmend an Bedeutung gewinnt.

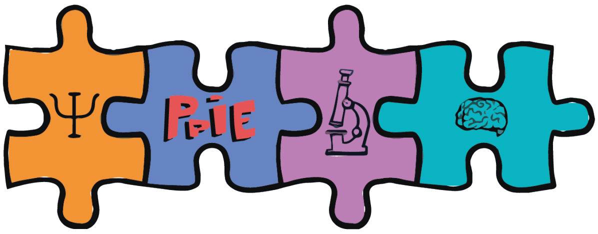

"Wenn du dir bei der Bedeutung mancher Begriffe unsicher bist, lies in deinem PPIE-Wörterbuch auf Seite 7 nach."

Direkt Betroffene bringen einen realen Erfahrungsschatz mit Behandlungsmethoden sowie ihren Aus- und Nebenwirkungen mit. Oder, um bei der Metapher des Apfels zu bleiben: Sie wissen nicht nur, wie der Apfel aussieht, sondern auch, wie er schmeckt. Zu lange wurde dieses Wissen aus der Praxis wenig berücksichtigt, was nicht nur zu Frustration führt, sondern auch die **Aussagekraft von Studien** schmälert. Und damit die Qualität der erforschten Behandlungsansätze.

Die Antwort auf diese Diskrepanz heißt **"Patient and Public Involvement & Engagement"**, kurz PPIE. Klingt sperrig, ist aber eine kleine Revolution: Das bedeutet, dass Forschung und Entwicklung im Gesundheitsbereich gemeinsam MIT oder sogar VON Patient:innen und Vertreter:innen der Öffentlichkeit durchgeführt werden, nicht ÜBER oder FÜR sie. PPIE steht also für eine Mitsprache und aktive Beteiligung in Forschung & Entwicklung.

- **PPIE** geht über die bisher gängigsten Formen der Partizipation, die hauptsächlich auf Fragebögen, Fokusgruppen und Interviews beruhen, hinaus.
- Bei PPIE werden Patient:innen direkt und von Anfang an **aktiv in Forschungsprozesse involviert:** Von der Formulierung der Forschungsfrage über die Antragstellung für die Finanzierung bis hin zum Sammeln und Auswerten der Daten.
- Die Forschung und die entwickelten Projekte profitieren dank des erweiterten, multidisziplinären Ansatzes von einer **besseren Forschungsqualität**.
- Sie erzielen insgesamt eine **höhere Wirksamkeit**, da bereits von Anfang an die Bedürfnisse von Patient:innen sowie ihr wertvolles Erfahrungswissen berücksichtigt werden.

Im Klartext: Für Forschungsgruppen ist ernsthafte Patient:inneneinbindung das Gebot der Stunde. Dieses Dokument ist eine **praktische Anleitung** dazu. Damit alle Puzzlestücke gemeinsam ein präzises Gesamtbild ergeben.

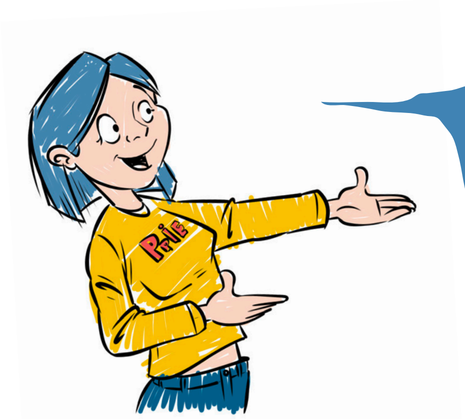

"In meinem Video erzähle ich noch mehr zu PPIE!"

SPIELREGELN

Wie wollen wir zusammenarbeiten?

Du kennst jetzt das Ziel dieses Workshops. Dann wollen wir noch kurz überlegen, wie wir zusammenarbeiten. Damit alle Spaß haben und optimal beitragen können.

- **Auf Augenhöhe:** Nutze Fachbegriffe bewusst und erkläre sie. Das Glossar, das du selbst ergänzen kannst, bietet sich hier an (nächste Seite).

- **Per Du:** Für eine gute und wertschätzende Zusammenarbeit bietet sich das Du-Wort und die Ansprache mit dem Vornamen an. Dadurch wird die Atmosphäre gelockert und ihr könnt leichter gemeinsame Ideen finden und kreativ arbeiten.

10 Gebote für gute Zusammenarbeit

"Dir fehlt eine Regel? Dann teile das gerne der Gruppe mit!"

1. „**Lasst euch aufeinander ein**." Lasst einander ausreden – jede Stimme soll gehört werden.
2. „**Geht wertschätzend miteinander um**." Respektiert einander und schätzt das Wissen, das jede:r miteinbringt und erreicht hat.
3. „**Verwendet eine lösungsorientierte Sprache**." Vermeidet Killerphrasen. Statt "Das wird sowieso nicht funktionieren" lieber „Lasst es uns versuchen!"

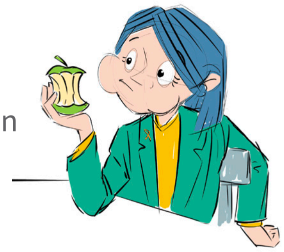

4. „**Seid konstruktiv**." Bietet Alternativen zu jeder Kritik.
5. „**Ersetzt ‚aber' durch ‚und'**." "Ein ‚aber' wirkt in der Kommunikation negativer als ein "und"; das verändert die Stimmung im Raum!
6. „**Übernehmt Verantwortung**." Einigt euch darauf, die gemeinsame Verantwortung für den Erfolg des Workshops zu tragen.
7. „**Seid im Hier und im Jetzt**." Schaltet „Ablenker" wie z.B. Smartphones und Pager auf lautlos.
8. „**Arbeitet ergebnisoffen**." PPIE bedeutet, den Mut zu haben, offen zu denken, um Lösungen außerhalb gewohnter Konventionen zu ermöglichen.
9. „**Betrachtet Perspektivenvielfalt als Chance**." Der Workshop ist ein „offener Raum" für Austausch – nutzt diese Chance, redet miteinander, tauscht eure Perspektiven aus und zeigt Bereitschaft, in die jeweils andere Welt einzutauchen.
10. „**Seid zuversichtlich**." Der Workshop bedeutet natürlich Zeit und Arbeit. Der PPIE-Prozess ist aber für alle Beteiligten ein echter Gewinn.

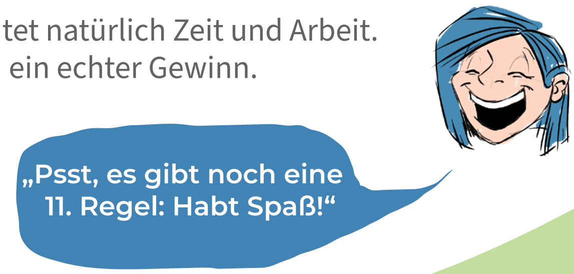

NAMENSSCHILD

Hier kannst du falten:

Platz für deinen Namen:

PPIE-Wörterbuch 01

Fachbereiche brauchen Fachbegriffe. Und die sind meist schnell erklärt – auch, wenn es um Begriffe rund um Diagnostik, Behandlung und Erforschung einer onkologischen Erkrankung geht. Damit wir im Workshop alle dieselbe Sprache sprechen, haben wir ein Glossar vorbereitet. Das Besondere: Du kannst es selbst erweitern.

So gehen wir im Workshop damit um: Wenn ein Fachbegriff vorkommt, soll die Person, die ihn verwendet, ihn auch erklären. Zusätzlich wird ein:e Teilnehmer:in darauf achten, dass dies auch tatsächlich passiert (wer diese Rolle übernimmt, klären wir vor Ort). Wir werden die jeweilige Erklärung dokumentieren, darüber hinaus kannst du sie in **dieses persönliche Glossar** eintragen.

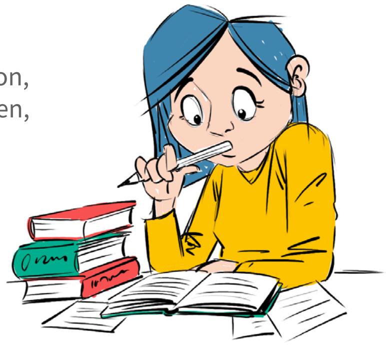

MEIN PPIE-WÖRTERBUCH

PPIE: Steht für "Patient and Public Involvement & Engagement", also für Beteiligung von Patient:innen und der Öffentlichkeit. Klingt sperrig, ist aber eine kleine Revolution: Bedeutet dieser Ansatz doch, dass Forschung und Entwicklung im Gesundheitsbereich **MIT oder sogar VON** Patient:innen und Vertreter:innen der Öffentlichkeit durchgeführt werden, nicht ÜBER oder FÜR sie. PPIE steht also für eine Demokratisierung von Forschung & Entwicklung.

Partizipation: Ein wesentlicher erster Schritt, wenn es um die Patient:inneneinbindung geht: Es handelt sich um eine **teilweise Beteiligung** von Patient:innen an Studien durch Fragebögen, Interviews, Fokusgruppen etc.

Engagement: Hiermit ist einerseits die **Bewusstseinsbildung** für ein Thema gemeint, wie etwa die Verbreitung von Informationen über die Forschung via (soziale) Medien, Wissenschaftsfestivals, Workshops oder Tage der offenen Tür. Andererseits aber auch die aktive Teilnahme von Patient:innen am Forschungsprozess durch die Übernahme von Aufgaben.

Involvement: The real thing! Gemeint ist das aktive, vollwertige Einbeziehen von Patient:innen in Forschungsprojekte. **Das heißt**: Gemeinsames Identifizieren von Forschungsthemen, Formulieren der Forschungsfragen, Stellen von Anträgen. Patient:innen sind Mitglieder in Steuerungsgruppen, helfen bei der Entwicklung von Infomaterialien, beim Durchführen von Interviews etc.

Patient Advocates: Das sind Personen – oft ehemalige Patient:innen – die als **Fürsprecher:innen** für die Belange von Patient:innen eintreten. Gegenüber Institutionen, der Ärzt:innenschaft, der Pharmaindustrie, der Politik, in diversen Gremien sowie in der Öffentlichkeit.

PPIE-Wörterbuch 02

Patient Organisation Representatives: Vertreter:innen von Organisationen, die für die Belange von Patient:innen eintreten. Sie bringen also die Ansichten ihrer jeweiligen Patient:innenorganisation ein.

Patient Experts: Der Name sagt es bereits: Diese Patient:innen sind echte Expert:innen! Es handelt sich um Personen, die einerseits wertvolle Erfahrung als Patient:innen mitbringen. Andererseits haben sie durch entsprechende Weiterbildung Fachexpertise in einem oder mehreren Fachgebieten. Dadurch können sie aktiv an Forschung & Entwicklung, Gesundheitspolitik etc. mitarbeiten.

Individual Patient: Bezieht sich auf den:die einzelne:n Patient:in. Wer selbst in den sauren Apfel beißen musste, hat auch jede Menge Erfahrungen gesammelt. Für viele Bereiche der Forschung ist dieses Wissen ein wertvoller Beitrag.

Carers (oder Caretakers): Der englische Begriff steht für alle Personen, die Patient:innen nahestehen und sie auf ihrem Pfad der Behandlung begleiten. Angehörige, Freund:innen, Vertrauenspersonen.

Weitere Begriffe können nach Belieben ergänzt werden!

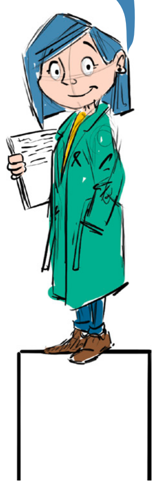

PPIE-Wörterbuch 03

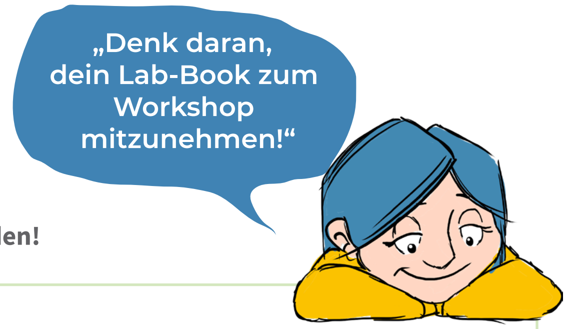

Weitere Begriffe können nach Belieben ergänzt werden!

GEDANKENPARKPLATZ

In diesem Workshop entstehen viele Eindrücke und neue Gedanken, die in diesem Gedanken-parkplatz bestens aufgehoben sind. Viel Spaß beim Befüllen!

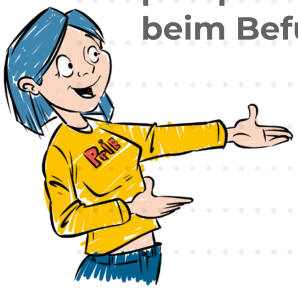

Mein Team 01

Titel des Workshops:

Hier treffen wir uns: Adresse/Institut eintragen

“Tauscht eure Kontaktdaten aus, um miteinander in Kontakt zu bleiben.”

Welche Rolle übernehmen deine Teamkolleg:innen und du?

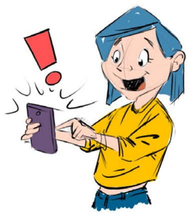

Zeitwächter:in – achtet darauf, dass der Zeitrahmen nicht gesprengt wird.

Fotograf:in – fotografiert die Ergebnisse, macht Bilder von der Gruppe und übermittelt diese im Nachgang an das Co-Moderationsteam.

Wandelndes Wörterbuch – notiert Fachbegriffe und Erklärungen auf einem Chart. Erinnert die Gruppe daran, Fachbegriffe zu erklären, wenn sie es vergessen.

Aber-Wächter:in – mischt sich ein, wenn jemand in einer Diskussion „aber” sagt und verwandelt dieses in ein “und”.

Ladegerät – achtet auf das Energie-Level im Raum und fordert Pausen ein, wenn es zu niedrig ist.

“Notiere dir gern, welche Rollen deine Teamkolleg:innen übernehmen.”

Das Moderationsteam und seine Unterstützer:innen

NAME/ROLLE	KONTAKTDATEN

Mein Team 02

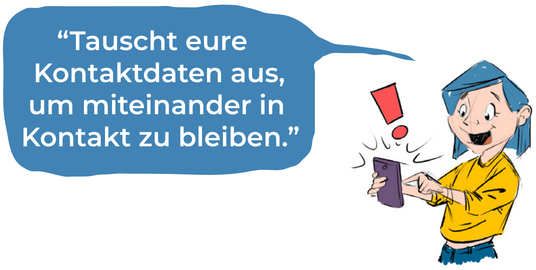

Die Workshopteilnehmer:innen

NAME / ROLLE	KONTAKTDATEN

Mein Team 03

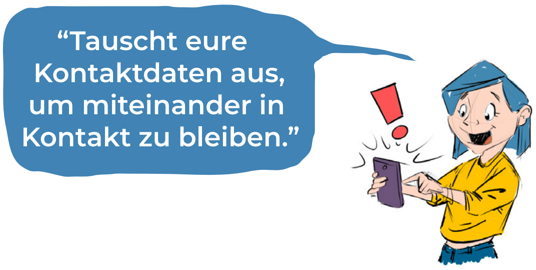

Die Workshopteilnehmer:innen

NAME / ROLLE	KONTAKTDATEN

GEDANKENPARKPLATZ

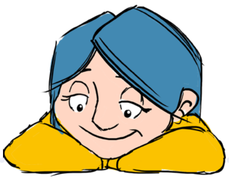

Avatar-Arbeitsblatt

BAUE DEINEN AVATAR

1. Bauen

Platz für deinen Avatar

2. Beschreiben

1. Welche Stärken hast du?

2. Welche Expertisen bringst du mit?

3. Welche besonderen Eigenschaften hast du?

4. Welche 5 wichtigen Meilensteine hast du bisher erreicht/erlebt?

3. Teile deinen Avatar mit der Gruppe

GEDANKENPARKPLATZ

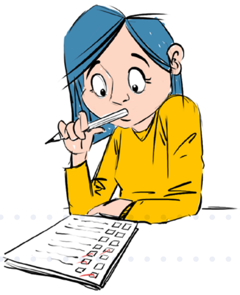

Meine Forschungsgruppe

Name eintragen:

Platz für Foto

So könnte unser Logo aussehen:

Das ist unser Ziel:

Dieses Forschungsvorhaben verfolgen wir:

Diese Stärken und dieses Expert:innenwissen bringen wir mit:

GEDANKENPARKPLATZ

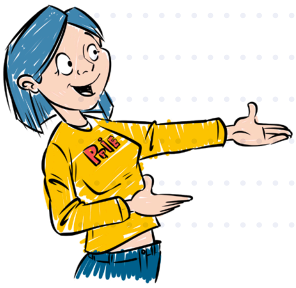

Die Wunderfrage

Stell dir vor, du wachst auf und das Problem

_______________ **ist gelöst.**

Woran würdest du bemerken, dass dieses Wunder geschehen ist?

Welche Probleme/Schwierigkeiten wären dadurch verschwunden?

Wer würde sonst noch erkennen, dass dieses Wunder geschehen ist und dass deine Probleme verschwunden sind?

GEDANKENPARKPLATZ

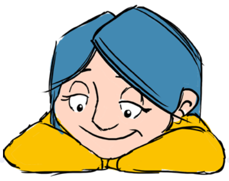

Erfindungs-Arbeitsblatt

BAUE DEINE ERFINDUNG

1. Bauen

Platz für
deine Erfindung

2. Beschreiben

1. Welches Problem löst deine Erfindung?

2. Welche Eigenschaften hat deine Erfindung?

3. Welchen Namen trägt deine Erfindung?

3. Teile deine Erfindung mit der Gruppe

GEDANKENPARKPLATZ

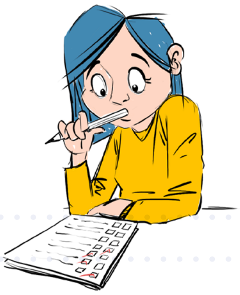

Wie läuft Forschung eigentlich ab?

1. Ausgangspunkt, Idee, Ziel

Welche Probleme lassen sich beobachten? Welche Mängel bestehen? Welche Fragestellungen ergeben sich? Wie sind Bedürfnisse beschaffen?

2. Literaturrecherche, Kooperationen

Was wissen wir bereits? Wer sind die Expert:innen?

3. Wissenschaftliche Fragestellung, Hypothesenbildung

Welche Frage können wir genau untersuchen?

4. Operationalisierung

Wie mache ich meine Frage messbar? Wie erkenne ich Erfolg/Misserfolg/eine Veränderung?

5. Untersuchungsplanung

Wer kann teilnehmen/wer nicht? Wie viele Personen sollen teilnehmen? Wann/Wo/Wie lange soll untersucht werden? Was ist zumutbar? Welche Methoden sollen angewandt werden? Sind sie fair/wiederholbar? Messen sie wirklich das, was sie zu messen vorgeben? Gibt es Störfaktoren? Gibt es eine Kontrollgruppe? Gibt es eine externe Kontrollinstanz (Ethikkommission)?

6. Datenerhebung

Welche Instrumente werden verwendet? Gibt es Standards oder Normen?

7. Datenauswertung, Interpretation

Sind die Daten geschützt (keine personenbezogenen Daten!)? Werden die Daten kontrolliert? Gibt es „Verzerrungen" (systematische Fehler) in den Daten? Welche statistischen Auswertungsmethoden sind anzuwenden?

8. Veröffentlichung, Verbreitung, Implementierung, Umsetzung

Wo sollen die Ergebnisse veröffentlicht werden? In welchem Medium und mit welchen Qualitätskriterien (Peer Review)? Sind die Ergebnisse zugänglich (Open Access)? Sind die Ergebnisse in der Praxis umsetzbar?

GEDANKENPARKPLATZ

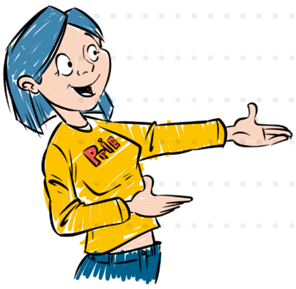

Der Studiendesign-Wegweiser 01

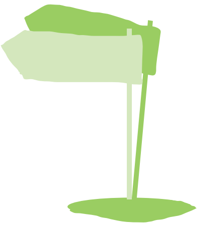

Die Forschungsfrage haben wir, kreative Lösungsansätze auch. Jetzt entwickeln wir ein **Studiendesign.** Versuche nun, jede Fragestellung **in Bezug auf die Forschungsfrage** bestmöglich zu beantworten und Unklarheiten zu notieren.

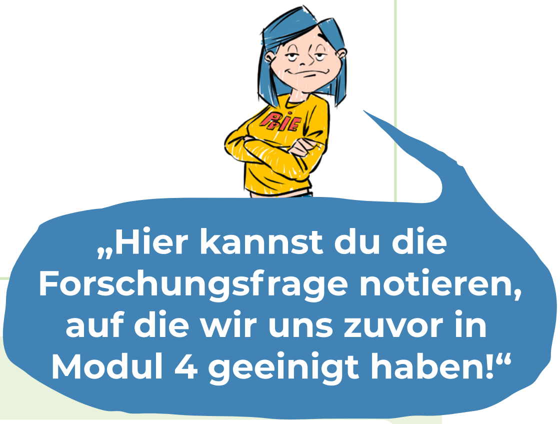

Planung

1. Welche Informationen brauche ich, um die Frage beantworten zu können?

Methodik

2. Welche Methoden/Strategien zur Infobeschaffung kenne ich? Welche Vor- und Nachteile gibt es?

Stichprobe

3. Wer in der Bevölkerung hat die Infos, die ich zur Beantwortung meiner Frage brauche? Sind es mehrere Personen? Wie viele?

Der Studiendesign-Wegweiser 02

Erhebung

4. Wann ist der geeignete Zeitpunkt, diese Infos einzuholen? Soll ich sie nur einmal oder öfter einholen? Soll ich sie über einen längeren Zeitraum sammeln?

Offenes

5. Was ist nach der Berücksichtigung dieser Punkte noch offen? Mit wem muss ich sprechen, wen fragen, einbeziehen etc., damit meine Frage beantwortet wird? Welche Risiken könnten auftreten?

Platz für Notizen

So geht‘s weiter

In unserem gemeinsamen Workshop haben wir **wertvolles Wissen** gesammelt und großartige Inhalte miteinander erarbeitet. Damit leisten wir als Team bereits einen **wichtigen Beitrag zu PPIE**.

„Mich interessiert jetzt aber:

Was wünschst du dir für den weiteren Verlauf dieses Projekts?“

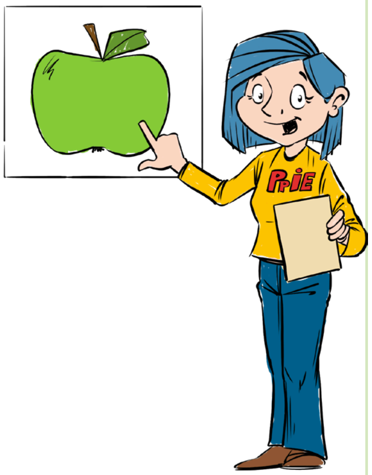

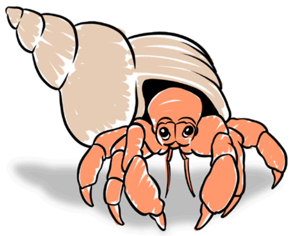

Hier kannst du fragen, wie es weitergeht und ob du über den weiteren Projektverlauf automatisch informiert wirst.

GEDANKENPARKPLATZ

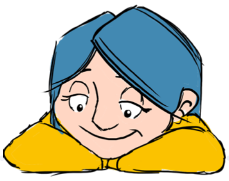

GEDANKENPARKPLATZ

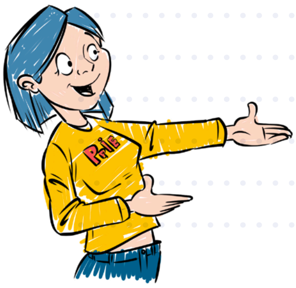

NOTIZEN

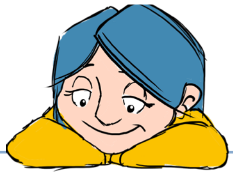

Zu dieser Methode mache ich mir Notizen:

Das ist die Kernbotschaft, die ich mitnehme:

NOTIZEN

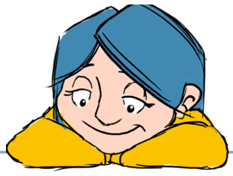

Zu dieser Methode mache ich mir Notizen:

Das ist die Kernbotschaft, die ich mitnehme:

NOTIZEN

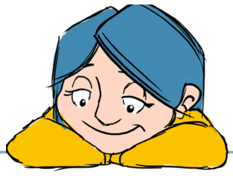

Zu dieser Methode mache ich mir Notizen:

Das ist die Kernbotschaft, die ich mitnehme:

NOTIZEN

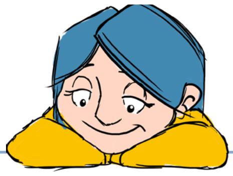

Zu dieser Methode mache ich mir Notizen:

Das ist die Kernbotschaft, die ich mitnehme:

NOTIZEN

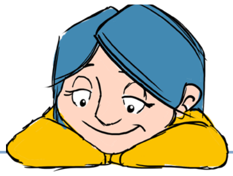

Zu dieser Methode mache ich mir Notizen:

Das ist die Kernbotschaft, die ich mitnehme:

NOTIZEN

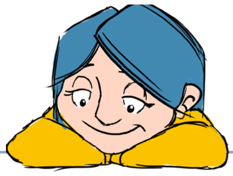

Zu dieser Methode mache ich mir Notizen:

Das ist die Kernbotschaft, die ich mitnehme:

NOTIZEN

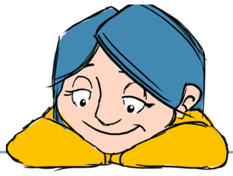

Zu dieser Methode mache ich mir Notizen:

Das ist die Kernbotschaft, die ich mitnehme:

NOTIZEN

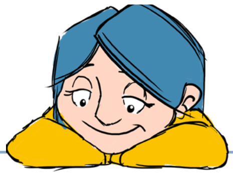

Zu dieser Methode mache ich mir Notizen:

Das ist die Kernbotschaft, die ich mitnehme:

Zum Schluss

Impressum

Ein Projekt der Medizinischen Universität Wien mit der COPE Group in Kooperation mit CCI Europe unterstützt von Kurvenkratzer, NF Kinder, EUPATI Austria und Survivors Österreich, finanziert durch das LBG Open Innovation in Science Center.

Medizinische Universität Wien, Universitätsklinik für Kinder- und Jugendheilkunde, Comprehensive Center for Pediatrics, Währinger Gürtel 18-20, 1090 Wien, www.meduniwien.ac.at.
CCI – Childhood Care International Europe, Handelskai 94-96, 1200 Wien, www.ccieurope.eu.

Illustrationen von Bone Buddrus, grafische Gestaltung und Design von Kurvenkratzer.

Projektteam: Gelebtes PPIE bedeutet ein interdisziplinäres und ganzheitliches Denken. Um eine Vielzahl an Perspektiven einzubringen, wurde das Projektteam bewusst aus Personen mit vielfältigen Expertisen zusammengestellt, die jeweils in verschiedenen Projektphasen ihre Beiträge leisteten.

Projektleitung:

Liesa J. Weiler-Wichtl, *Klinische und Gesundheitspsychologin, Medizinische Universität Wien/COPE Group*
Carina Schneider, *Psychologin, Patient Expert, CCI Europe*

Konzeption und Umsetzung:

Ulrike Leiss, *Klinische und Gesundheitspsychologin, Medizinische Universität Wien/COPE Group*
Hannah Gsell, *Psychologin, Patient Expert, CCI Europe, Survivors Österreich*
Anna Maletzky, *Klinische Psychologin, Medizinische Universität Wien*
Johannes Gojo, *Arzt, Medizinische Universität Wien*
Anita Kienesberger, *Patient Advocate, CCI Europe*
Claas Röhl, *Patient Advocate, NF Kinder, EUPATI*
Martina Hagspiel, *Patient Advocate, Kurvenkratzer*
Albina Hyseni-Bocolli, *Projektkoordinatorin, CCI Europe*
Lena Horvath, *Projektkoordinatorin, Kurvenkratzer*
Florian Lems, *Workshopkonzeptentwickler, Kurvenkratzer*
Tanya Kaindlbauer, *Lektorin, Kurvenkratzer*
Lena Kalinka, *Grafikerin, Kurvenkratzer*
Bone Buddrus, *Illustrator*

Involvierte Individual Patients & Carers und weitere Projektmitarbeiter:innen (Public Involvement):

Bettina Baldt, *Psychologin*
Lisa Cate, *Patient Expert*
Monika Czajka, *Patient Expert*
Sabine Karner, *Patient Expert*
Anna Lämmerer, *Patient Expert*
Rita Hansl, *Wissenschaftliche Assistentin*
Maximilian Hopfgartner, *Wissenschaftlicher Assistent*
Niklas Schober, *Individual Patient*
Delphine Heenen, *Patient Advocate*
Alice Gerbaux, *Patient Advocate, inhaltlicher Berater*

Marie Therese Gubi, *Patient Expert*
Maria Willomitzer, *Patient Expert*
Valentin Willomitzer, *Patient Expert*
Anna Zettl, *Patient Expert*
Sybille Zettl, *Patient Advocate*
Stefan Pinczolits, *Patient Expert*
Doris Schober, *Patient Expert*
Florian Müller, *Patient Expert*

Daniela Koczera, *Individual Patient*

Und zahlreiche weitere Individual Patients & Carers.

Gefördert durch:

LUDWIG BOLTZMANN GESELLSCHAFT
Open Innovation in Science Center

Das Projekt „Nothing about us without us“ sowie die „Junior research Academy“ wurden 2021 und 2022 im Rahmen des Calls für Public and Patient Involvement gefördert durch: LBG Open Innovation in Science Center Program Manager Public and Patient Involvement in Research

Nußdorfer Straße 64/2
1090 Vienna, Austria
www.ois.lbg.ac.at

Tel +43 (0) 1 513 27 50-68
M +43 (0) 660 215 57 41
thomas.palfinger@lbg.ac.at

Umgesetzt von:

MEDIZINISCHE UNIVERSITÄT WIEN
COPE GROUP
Childhood Cancer International EUROPE
KURVENKRATZER

In Kooperation mit:

EUPATI
NF Kinder
survivors AUSTRIA
